# Tropistry: A registry-based modular platform to improve care of neglected tropical diseases in nonendemic settings—Study protocol with two targeted conditions: acute schistosomiasis and cutaneous/mucocutaneous leishmaniasis

**DOI:** 10.1371/journal.pone.0335032

**Published:** 2025-10-30

**Authors:** Carlos Bea-Serrano, Sami Alcedo, Nicole Berens-Riha, Saskia van Henten, Johan van Griensven, Steven Van Den Broucke, Patrick Soentjens, Federico Gobbi, Emmanuel Bottieau

**Affiliations:** 1 Infectious Disease Unit, Department of Internal Medicine, Clinic University Hospital of Valencia, INCLIVA Biomedical Research Institute, Valencia, Spain; 2 Department of Clinical Sciences, Institute of Tropical Medicine, Antwerp, Belgium; 3 Department of Infectious-Tropical Diseases and Microbiology, IRCCS Sacro Cuore Don Calabria Hospital, Negrar (Verona), Italy; 4 Department of Experimental and Clinical Sciences, University of Brescia, Brescia, Italy; University of Nairobi Faculty of Health Sciences, KENYA

## Abstract

**Background:**

Neglected tropical diseases (NTDs) are increasingly encountered in nonendemic settings due to migration, international travel, and global mobility. Their clinical management is often challenging: evidence from endemic regions is limited or not directly applicable, and healthcare providers are frequently unfamiliar with their diagnosis and treatment. Consequently, there is substantial heterogeneity in diagnostic workup, therapeutic choices, and follow-up strategies, which may affect patient outcomes and complicate guideline development. Given the sporadic presentation of these diseases across individual centers and the overall low caseload in nonendemic settings, conducting randomized controlled trials to define optimal strategies is largely unfeasible. To address these gaps, we designed Tropistry, a modular, multicenter registry that integrates harmonized data collection with embedded expert-informed clinical guidance.

**Methods:**

Tropistry is an ambispective, multicenter registry planned for implementation across Belgian and European sites. Its modular structure will allow the sequential integration of disease-specific components, initially focusing on acute schistosomiasis and cutaneous/mucocutaneous leishmaniasis (CL/MCL), selected for their sporadic occurrence, clinical complexity, and lack of standardized management. Data will be collected through REDCap-based electronic case report forms aligned with up-to-date, evidence-based recommendations to ensure harmonized and comparable data collection. Embedded expert-informed guidance will support clinicians unfamiliar with these diseases, complemented by expedited access to ITM experts via TROPmail and aggregated feedback through an interactive R Shiny-based dashboard. A structured evaluation framework will assess feasibility, usability, data quality, user engagement, and treatment adherence to ensure the platform’s utility and validity.

**Expected impact:**

By centralizing harmonized data on diagnostic and therapeutic practices, Tropistry aims to address critical knowledge gaps in managing NTDs in nonendemic settings. For the initial targeted conditions, it will help clarify optimal strategies, such as the combination of corticosteroids and praziquantel for acute schistosomiasis and best practices for therapy and follow-up in CL/MCL. Beyond data collection, Tropistry will provide embedded expert-informed guidance, interactive dashboards, and direct access to ITM experts to support clinicians and promote standardized care. Its modular design will enable expansion to other challenging NTDs, while the built-in evaluation framework ensures continuous assessment and optimization of feasibility, usability, and clinical utility. Ultimately, Tropistry seeks to strengthen collaboration between reference centers and non-specialized facilities and generate robust real-world evidence to inform clinical practice, guideline development, and future trials.

## Introduction

Neglected tropical diseases (NTDs) represent a substantial global health burden, particularly in low-resource tropical regions [1]. However, globalization has recently blurred traditional geographic boundaries, and NTDs are now increasingly reported in nonendemic countries as a result of the growing trend in travel and migration as well as climate change [2–4]. In 2024, international travel exceeded 1.4 billion trips, nearly returning to pre-pandemic levels [5], while 4.3 million new migrants entered Europe in 2023 [6]. In parallel with evolving ecology, vector-borne NTDs are progressively spreading to previously unaffected temperate areas, including parts of central Europe [7]. In Southern Europe, leishmaniasis, historically endemic, has shown notable epidemiological shifts, including urban outbreaks and the emergence of new animal reservoirs [8]. West Nile virus disease, though a more recent arrival, has become established with recurrent seasonal transmission in countries such as Spain and Italy [9]. In contrast, diseases like dengue and schistosomiasis—previously unseen in Europe as locally transmitted infections—have begun to emerge through sporadic autochthonous outbreaks, typically following imported cases and transient environmental conditions conducive to transmission [10,11]. Consequently, clinicians in nonendemic settings are increasingly confronted with conditions they are not familiar with, and for which standardized management protocols are often lacking [12,13].

The clinical management of NTDs in nonendemic regions implies multiple challenges. Cases typically arise sporadically—either isolated or in small, unpredictable clusters [14]. Patients—often travelers or recently arrived migrants—may present with atypical or non-classical clinical features [15]. Care is frequently fragmented, as most first-line providers lack training in these diseases, leading to delayed referrals to specialized clinics. Moreover, there is substantial heterogeneity in diagnostic and treatment practices not only between these referral institutions [12,16,17] but also among providers within the same center [18]. In the absence of widely available or disseminated guidelines, clinical decisions are often guided by individual physician experience, local preferences or site-specific treatment availability [12,16]. While European healthcare systems may have access to advanced diagnostics and supportive therapies, they often lack clinical protocols tailored to nonendemic facilities or in-country approved drugs for NTDs [12,15,16,18–20], and may also be inaccessible to marginalized populations, including undocumented migrants.

The lack of context-specific evidence remains a key limitation for optimal NTD care in nonendemic areas [12,15,20]. Conducting high-quality research in resource-limited endemic settings is extremely challenging [21,22], and data generated may not be fully applicable to other environments [15]. In nonendemic areas, the low and scattered caseload makes randomized controlled trials (RCTs) largely impractical when there is no pre-established scientific collaboration. Small sample sizes across multiple centers and countries reduce statistical power and increase susceptibility to bias [23]. Additionally, heterogeneity in case definitions, therapeutic protocols, and outcome measurements may lead to major inconsistencies and complicate data analysis and knowledge synthesis. Table 1 summarizes the key barriers to generating high-quality clinical evidence on NTDs in nonendemic settings.

**Table 1:** Key barriers to evidence generation for NTDs in nonendemic settings.

Barrier	Description	Impact	Refs
**Sporadic distribution**	Rare, dispersed cases across many centers	Small sample sizes; randomized trials hardly feasible	[14,23,24]
**Clinical heterogeneity**	Variable species, lesions, host factors	Limits standardization	[16,24,25]
**Atypical presentation**	Non-classical features in travelers	Delayed inclusion; outcome bias	[12,15,18]
**Lack of harmonized guidelines**	Few protocols in nonendemic settings	Fragmented management	[12,16,24]
**Regulatory variability**	Divergent ethics and consent norms	Hinders multicenter studies	[21,26]
**Inconsistent drug availability**	Key treatments unavailable or not reimbursed	Affects comparability	[17,27,28]
**Follow-up challenges**	Mobile patients; fragmented care	Incomplete follow-up and outcomes	[12,18,24]
**Poor data structure**	Lack of standardized records	Missed research opportunities	[20,29]

Furthermore, the absence of comprehensive clinical datasets—including treatment outcomes, safety profiles, and long-term follow-up—can hinder regulatory approval and reimbursement for essential therapies for NTDs within European health systems [17,27,28]. This contributes to persistent inequities in access to adequate care for the affected populations.

In this complex and evolving context, observational registries may offer a viable alternative to, or a preliminary step before, randomized trials for generating sufficient evidence on treatment effectiveness, safety, and practice variability regarding NTDs. Building high-quality datasets through collaborative networks implies some minimal harmonization of case definitions, clinical practices, and outcome measures across sites, laying the groundwork for future trials whenever necessary and feasible. With these considerations, a panel of experts at the Institute of Tropical Medicine (ITM), Antwerp, the national reference center for tropical diseases in Belgium, has developed a treatment registry, called Tropistry, aimed at addressing critical knowledge gaps in the clinical management of NTDs in Belgium and other nonendemic countries in Europe. It is designed as a flexible, modular and bidirectional platform that, on the one hand, allows physicians confronted with selected NTDs collecting systematic and structured clinical data to be further analyzed by ITM experts. On the other hand, provides them embedded state-of-the-art guidance and expedited additional support through email/phone if necessary.

Several regional and international initiatives already exist with similar purposes. Networks of reference travel clinics and tropical medicine institutes have substantially contributed to syndromic monitoring, geographical mapping and epidemiological trends of tropical diseases imported to Europe and other nonendemic regions [14,17,25,30]. As the primary focus is on epidemiological surveillance and outbreak detection, there is no collection of detailed clinical data on treatment regimens and outcomes that could support harmonized clinical decision-making. GeoSentinel is the most established global network that specifically monitors travel-related infections, through enhanced diagnostic reporting [14,31,32]. However, it has limited collection of clinical data and lacks information on treatment and outcome. TropNet is a European network that also contributes to surveillance and sharing of expertise on tropical diseases but does not currently collect systematic data on treatment practices and longitudinal outcomes [17]. The LeishMan network focuses on epidemiological surveillance and expert diagnosis of leishmaniasis across Europe [16], but the description of the clinical cases does not provide in-depth information that could immediately impact its optimal management in terms of treatment and follow-up. The European Register of Cystic Echinococcosis (ERCE) aims at collecting data on management according disease staging, but limitations in completeness and consistency have restricted its ability to inform clinical practice or support comparative analyses [30]. Finally, the Chagas nonendemic cohort (ChaNoE) project was recently launched in Spain to harmonize diagnostic and follow-up protocols for Chagas disease and support biobanking and multicenter collaboration [19].

Despite these valuable efforts, there remains a need for a unified and harmonized platform at national and European level that can capture robust real-life data and support non-expert clinicians in the management of NTDs. Here we describe the objectives and methodology of such an initiative, called Tropistry. We provide the protocols for two NTDs we plan to target in the registry initially, i.e., acute schistosomiasis and cutaneous/mucocutaneous leishmaniasis (CL/MCL).

### Generic objectives of Tropistry

The primary objectives of Tropistry are threefold: (1) to establish a harmonized modular registry that contributes generating high-quality real-world evidence about treatment practices and outcomes of several relevant NTDs in Belgium; (2) to provide state-of-the-art and individualized guidance for clinicians less familiar with these conditions; and (3) to evaluate Tropistry’s implementation, including its feasibility in routine practice, usability for clinicians, data quality, and influence on treatment adherence across participating sites. The general purpose of this project is to optimize clinical care and inform guideline development and future trials.

The secondary objectives include:

- Description of variability in diagnostic, therapeutic and follow-up practices in Belgian and other European health facilities
- Evaluation of treatment effectiveness and monitoring of adverse events in routine care
- Provision and regular update of expert-informed guidance embedded within data collection tool
- Identification of deviations from recommended treatment and analysis of underlying reasons
- Identification of factors linked to favorable outcomes, relapse, or complications for the different NTDs under study
- Generation of evidence for regulatory bodies when randomized trials are not feasible

Tropistry aims to address most of the current gaps of similar registries, by bridging more directly empirical care and structured evidence generation. Its dual role as both a research platform and a clinical decision-support tool distinguishes it from existing initiatives. While it was initially purposed for NTD care in Belgium, further expansion to, and collaboration with, other similar European facilities is being considered.

## Materials and methods

### Design and implementation of Tropistry

**Fig 1:**
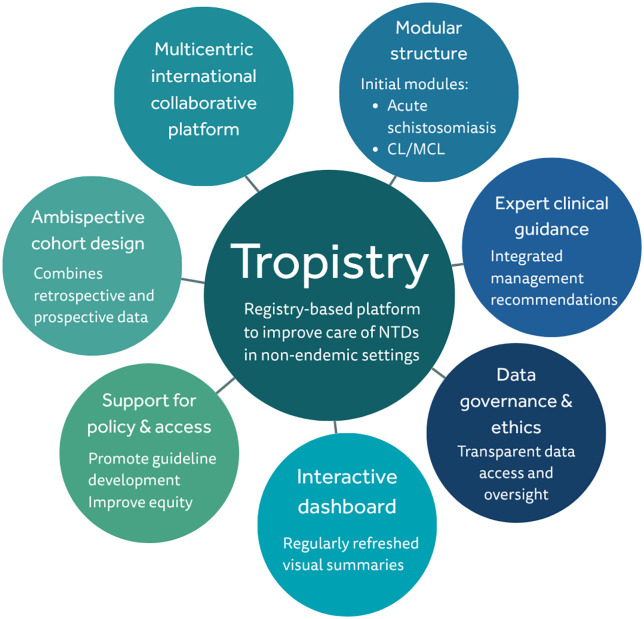
Conceptual framework of Tropistry platform.

Tropistry is a pragmatic, flexible, modular, ambispective, bidirectional multicenter registry designed to capture real-world data and to provide expert guidance on NTD care in nonendemic settings. Both retrospective and prospective cases may be included, with eligibility starting from January 2020 to capture recent case management, especially following the post-pandemic resurgence of travel and migration. Sites may adopt retrospective, prospective, or hybrid enrollment, depending on local capacity and regulation. The conceptual framework is presented in Fig 1, where the initial two disease modules are mentioned (acute schistosomiasis and CL/MCL; see below).

As mentioned, Tropistry is currently hosted at ITM, Antwerp, Belgium, as part of ITM’s national reference duties. Belgian academic hospitals and referral centers, as well as other European centers, are invited to join to both provide field data and benefit from updated guidance. Standardized General Data Protection Regulation (GDPR)-compliant REDCap-based case report forms (CRFs) are designed for each targeted disease within specific modules, and generic expert guidance is embedded in the data collection tool. A R Shiny-based dashboard aggregating relevant epidemiological and clinical data from all participating institutions is accessible to all partners in this project. No study-specific interventions are required: clinical decisions remain entirely at the discretion of treating physicians, who are free to follow the guidance or request additional support.

### Study population and enrollment

Eligible participants include patients of any age diagnosed with one of the target diseases, based on predefined case definitions and managed at a participating site. Depending on the disease under study only confirmed and probable cases will be included. Patients participating in other studies may also be enrolled. While no formal sample size has been calculated due to the exploratory nature of this study, a target of at least 100 patients per disease module is set before meaningful preliminary analysis is undertaken. Exclusion criteria are minimal: patients without sufficient diagnostic information or consent (written, oral, or opt-out per local policy) are excluded. Sites operating under opt-out consent policies must ensure proper notification. Each site designates a trained clinician and a backup coinvestigator responsible for enrollment and REDCap data entry. Patients can be included at any point in their clinical course, though early inclusion is encouraged to facilitate follow-up and benefit from embedded tools. Patients will be managed according to the local routine care or the suggested guidance, and no financial compensation is considered.

### Data collection and core variables

Data is collected using REDCap-based disease-specific CRFs. Centers enter pseudonymized information at baseline and, when feasible, during follow-up or at discharge. The core dataset includes:

- Demographics (e.g., age group, sex, comorbidities)
- Epidemiologic history (e.g., travel, migration, exposure)
- Clinical data (e.g., symptoms, physical findings, laboratory results such as eosinophil cell count or C-Reactive Protein values)
- Specific and supporting diagnostics (e.g., PCR, microscopy, serology, imaging)
- Treatment details (e.g., drug names, doses, durations, sequences)
- Outcomes (e.g., clinical resolution, lesion healing, microbiological clearance)
- Adverse events and complications as documented in the medical record

While follow-up intervals may vary, standardized timepoints and outcome definitions are recommended to support comparability. Regular internal quality checks by the REDCap software and query resolution procedures via the coordinating group at ITM will help ensure data reliability.

### Statistical analysis

Descriptive statistics will summarize baseline characteristics, diagnostic methods, treatments, and outcomes. Categorical variables will be reported as frequencies and percentages; continuous variables as means with standard deviations or medians with interquartile ranges, as appropriate. Time-to-event outcomes (cure, relapse, death, adverse event) will be analyzed using Kaplan–Meier and Cox models. Associations between treatments and outcomes will be assessed using univariate and multivariable regression, adjusting for potential confounders such as age, immune status, or disease severity. Analyses will be performed using validated statistical software and will be supported by a statistician to ensure methodological rigor.

### Evaluation of utility and validity

The implementation of Tropistry will be evaluated across several dimensions to ensure its utility and validity. We will monitor the number of participating centers, patient recruitment rates, and the integration of the registry into routine workflows to assess feasibility. Usability will be explored through periodic surveys and interviews with clinicians, focusing on data entry processes, clarity of embedded guidance, and practicality during patient care. Data quality will be maintained using REDCap’s validation rules, audit logs, and regular data audits, complemented by targeted manual reviews from the coordinating team. User engagement will be tracked via metrics such as data entry frequency, completeness of follow-up records, and interaction with support tools like TROPmail and the interactive dashboard. Finally, treatment practices across sites will be compared with Tropistry’s expert-informed recommendations, and feedback will be provided to participating centers to promote harmonization and continuous quality improvement.

### Ethical considerations and governance

Tropistry was approved by the ITM Institutional Review Board (Ref: 1704/24; 18 November 2024), with local approval required at each participating site. The registry adheres to the Declaration of Helsinki, Good Clinical Practice, and relevant data protection regulations. Informed consent is obtained per local policy. As approved by the ITM IRB, inclusion criteria include oral consent to participate (in sites with an institutional opt-out strategy, with notification in the medical file), or a signed informed consent (possibly provided by a legal representative) in other sites (see Supporting Information S1 Protocol). All data are pseudonymized before REDCap entry, with identifiers stored securely and locally. REDCap provides secure access, audit trails, and regular backups to ensure data integrity. Requests for secondary data use must be submitted to the Tropistry Scientific Committee and are reviewed based on scientific merit and alignment with registry goals. The Scientific Committee is initially composed of ITM experts and will be progressively expanded to include representatives from other participating institutions as the initiative grows. In case of scientific publications, all contributors will be appropriately recognized using a group authorship model (e.g., “and the Tropistry Group”). At least one representative from each main contributing center will be included as a named author. The definition of a main contributing center will vary by disease module, based on the actual number of cases contributed in relation to the overall dataset. Aggregate, de-identified findings will be shared via an interactive dashboard based on R Shiny App to promote transparency and provide timely feedback to participating centers.

### Inclusivity in global research

Additional information regarding the ethical, cultural, and scientific considerations specific to inclusivity in global research is included in the Supporting Information (S1 Checklist)

### The case for two challenging NTDs: Acute schistosomiasis and cutaneous/mucocutaneous leishmaniasis

**Fig 2:**
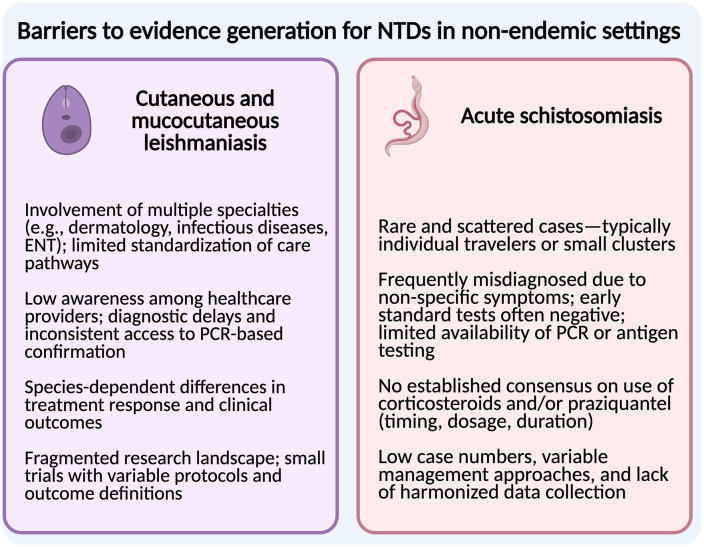
Key barriers to evidence generation for acute schistosomiasis and CL/MCL. Created in BioRender. Bea, C. (2025) https://BioRender.com/oucx7kk.

The registry initially focuses on two NTDs that have very distinct epidemiology and clinical presentation but share therapeutic challenges and key barriers to generating clinical evidence in nonendemic settings, as summarized in Fig 2. The full protocol including both initial conditions has been approved in 2024 by the ITM Institutional Review Board and is available as S1 Protocol. The full list of variables included in the REDCap-based CRFs for both modules is provided as Supporting Information (S1 and S2 Files).

### Acute schistosomiasis

#### Background and rationale.

Acute schistosomiasis (historically called Katayama syndrome when symptomatic) is a rare, often unrecognized hypersensitivity reaction that typically develops 3–12 weeks after freshwater exposure to *Schistosoma* cercariae in endemic areas, predominantly in sub-Saharan Africa. It frequently affects young travelers or family groups in clusters [18,33]. Clinical presentation—fever, cough, myalgia, headache—with eosinophilia is nonspecific and may mimic other tropical infections, often delaying diagnosis. Standard diagnostic tools such as microscopy and serology have poor sensitivity in the early phase with positivity rates below 70% even when combined [34]. Although promising, molecular (e.g., polymerase chain reaction [PCR]) and antigen-based assays (e.g., circulating anodic antigen [CAA]) remain largely unavailable outside reference centers [35,36]. Treatment practices are heterogeneous and not standardized. Praziquantel is commonly used but has limited efficacy against juvenile worms [37]. Corticosteroids are frequently added to modulate inflammation, but optimal timing, dosage, and duration remain uncertain, with no evidence-based consensus across expert networks [12]. This diagnostic and therapeutic ambiguity underscores the pressing need for innovative ways to generate evidence.

#### Methods and guidance.

Composite case definitions for Tropistry rely on recent consensus criteria (Table 2) [33]. Cases are classified as confirmed, probable, or possible based on exposure history, clinical presentation, and diagnostics (e.g., serology, microscopy, antigen detection, or PCR). Only confirmed and probable cases are to be included. Data collection follows the generic flow described above, with recommended reassessments taking place, ideally, at 6–12 weeks post-treatment and, when feasible, again at one year. Standardized outcome definitions (Table 3) enable cross-site comparison. As already highlighted, brief embedded guidance supports clinical decisions. In Tropistry, the recommended treatment is based on a recent publication of a prospective clinical study that favored short-course therapy for acute schistosomiasis, in terms of safety and effectiveness [38]. The guidance will be updated as new evidence emerges.

**Table 2:** Acute schistosomiasis case definition [33].

Case definition	Epidemiological criteria^a^	Microbiological criteria	Clinical criteria
**Confirmed acute schistosomiasis**	Yes	Eggs, CAA, PCR, or seroconversion	Optional^b^
**Probable acute schistosomiasis**	Yes	Serology or CCA	Optional^b^
**Possible acute schistosomiasis**	Yes	No	Yes
**High epidemiological suspicion**	Yes	No	No

CAA=circulating anodic antigen. CCA=circulating cathodic antigen.

^a^Epidemiological criteria include travelers to an endemic area with no previous history of infection and possible infective contact from 3 weeks to 3 months before.

^b^Clinical criteria can be absent if there is no evidence of previous exposure.

**Table 3:** Primary and secondary outcomes for acute schistosomiasis in Tropistry.

Primary outcome	Secondary outcomes
Resolution of clinical symptoms within 2 weeks after treatment initiation	• Normalization of eosinophil count (<0.5 × 10⁹/L) • No new organ involvement during follow-up • Treatment-related adverse events (graded by severity) • Retreatment or additional corticosteroid requirement

### Cutaneous and mucocutaneous leishmaniasis

#### Background and rationale.

CL/MCL has become one of the most frequently reported travel-acquired infections. Hundreds of cases have been documented in returning travelers through GeoSentinel [14], and the disease is now increasingly diagnosed in nonendemic countries like Belgium [39]. In some regions such as Southern Europe, the epidemiological picture is even blurred by autochthonous cases [40], and there is even a theoretical risk of secondary local transmission where competent vectors are present [7]. In addition, conflict-driven migration, such as that caused by the Syrian civil war, has triggered a major CL outbreak, prompting global concern [41]. Clinical management of CL/MCL is hindered by significant heterogeneity in *Leishmania* species, clinical presentations, treatment options, and outcomes [16]. Leishmaniasis is traditionally divided into Old World and New World forms based on species and geographic distribution. Old World species (e.g., *L. infantum, L. major, L. tropica*) are found in Southern Europe, Asia, and Africa, causing substantial skin morbidity. New World species—mainly from the *Viannia* subgenus, including *L. (V.) braziliensis* and *L. (V.) guyanensis*—are endemic to Central and South America and more often associated with mucosal complications. Both CL and MCL can result in disfiguring scars and even mutilation, particularly when facial areas are affected, leading to significant psychosocial consequences [42]. Treatment decisions are often empirical and based on limited evidence. Therapies may be topical, systemic, or combined, with choices influenced by species, lesion severity, anatomical location, and host factors [25,43,44]. However, no universally accepted treatment algorithm exists, and therapeutic responses may be variable [39]. Systematic reviews consistently report wide variability in therapeutic criteria, outcome definitions, and study designs, with most trials being small, fragmented, and methodologically limited [29]. While harmonized trial methodologies have been proposed and collaborative groups have made progress in standardizing eligibility and outcome criteria [45–47], high-quality, generalizable evidence remains lacking. Repeated calls have emphasized the need for a coordinated, multicenter platform trial to evaluate CL/MCL treatments, particularly among international travelers [24].

#### Methods and guidance.

CL/MCL is defined by compatible skin or mucosal lesions with parasitological confirmation. Cases with characteristic features and relevant exposure but no laboratory confirmation may be enrolled as “clinically compatible” and flagged accordingly. The CRF records in detail lesion characteristics (number, size, location), species identification (if available), host immune status, and mucosal involvement (baseline and follow-up). To address classification inconsistencies across guidelines, Tropistry uses in Belgium a harmonized “complex CL” definition proposed by Vandeputte et al. [39], that incorporates IDSA and LeishMan criteria to make them congruent (Table 4). This does not prevent, however, the inherent heterogeneity of the current criteria of complex CL (from small lesion with esthetical impact to underlying immunosuppression or relapse/non-response). Treatment fields capture drug regimen, route, dosage, and duration. Outcome assessment is recommended at standardized timepoints at Day 42, 90, and 180 [45,47]. Long-term sequelae (e.g., scarring, pigmentary changes) are assessed using LeishCOM_LCL domains [46] (Table 5). When feasible, clinical photographs are uploaded via a secure REDCap module, with proper consent. Embedded guidance in this module focuses on species-specific therapeutic decisions.

**Table 4:** Definition of complex cutaneous leishmaniasis according to IDSA, the LeishMan group and Vandeputte et al.

Criteria	IDSA [48]	*LeishMan* group [44]	Vandeputte et al. [39]
Size	>4 cm	>3 cm	>4 cm
Number of lesions	>4	>3 or>1^b^	>4
Location	Face, ears, eyelids, lips, fingers, toes, joints, genitalia	“Delicate location”/ ‘cosmetically disfiguring’	Ears, eyelids, nose, lips, joints, fingers, toes
Mucosal involvement	Yes	Yes	Yes
Immunosuppression	Yes	Yes	Yes
Failure of local therapy	Yes	Yes	Yes
DCL/LR	Yes	Unclear	Yes
Subcutaneous nodules	Yes	Unclear	Yes
Lymphatic spread	Yes	Yes	Yes
Infection in Bolivia	Unclear	Yes	Unclear

DCL: diffuse cutaneous leishmaniasis, IDSA: Infectious Disease Society of America, LR: Leishmania recidivans

a For the IDSA guidelines there are cases where the simple and complex is not mutually exclusive; e.g., 1–4 cm size, and 2–4 lesions.

b A distinction is made between Old-World species (with the addition of *L. mexicana* from the New-World)*,* for which more than 3 lesions are necessary to qualify for complex CL, while for New-World species (except for *L. mexicana*) having more than one lesion is sufficient.

**Table 5:** Primary and secondary outcomes for cutaneous/mucocutaneous leishmaniasis in Tropistry.

Primary outcome	Secondary outcomes
Final cure: complete cure (re-epithelialization for ulcers or flattening for non-ulcerated lesions) at 6 months for CL and 12 months for MCL	• Treatment failure: < 50% re-epithelialization or flattening by day 42, and <100% by day 90 • Recurrence of lesions within 12 months • Severity and type of scar formation at 6 months • Treatment-related adverse events (graded by severity)

CL: cutaneous leishmaniasis, MCL: mucocutaneous leishmaniasis.

#### Study status and timeline.

Following ethical approval within our institution in 2024, the REDCap CRF for the acute schistosomiasis module have been finalized in May 2025. Subsequently, 15 retrospective cases from the ITM travel clinic have been included in June 2025. Several Belgian academic hospitals have expressed interest in participating in this module. There is also an ongoing dialogue about this initiative with the TropNet consortium, which welcomed this project and considers to expand inclusions in several of its member tropical/travel institutions as well. Inclusions in the CL/MCL module have started at ITM, at the time of writing, but will be initially restricted to Belgian centers as the numbers are much larger than for the first module, before consideration any expansion in complementarity to the Leishman consortium. Continuous enrollment will follow, without a fixed end date. Data analysis will proceed on a rolling basis. Key findings on treatment practices and outcomes will be summarized periodically and shared through the interactive web dashboard, conference presentations, and peer-reviewed publications to inform guidelines and support patient care.

## Discussion

In nonendemic settings, the low incidence and clinical heterogeneity of NTDs limit the feasibility of RCTs to inform treatment decision, limiting the generation of robust, practice-oriented evidence. Tropistry was developed as a pragmatic response to this gap, initially for improving care in Belgium. By combining expert-informed guidance with standardized definitions and harmonized follow-up protocols, the registry enables consistent and detailed observational documentation of treatment practices and outcomes, which can reach satisfactory evidence. Its modular and adaptable design supports real-world evidence generation across diverse clinical settings and evolving healthcare needs. This section highlights key features of the platform and its potential contributions to clinical research, care harmonization, and health policy.

### Modular design and scalable expansion

Tropistry is constructed around modules that each correspond to target diseases. This allows data collection tailored to the scientific needs, as knowledge gaps may be very different according to the disease under consideration (diagnosis, staging, treatment, follow-up). It is also designed for scalable expansion. New modules—for example, for neurocysticercosis or disseminated strongyloidiasis—could be proposed by participating sites and reviewed by the Scientific Committee. All modules follow a consistent structure: harmonized case definitions, tailored CRFs, and context-specific guidance. Participating centers are supported with onboarding materials, standard case examples, and access to technical and clinical coordination.

### Evaluation of platform utility and validity

To ensure that Tropistry fulfills its intended role, we have embedded a formal evaluation component within the study design. This approach allows us to measure the platform’s feasibility, user experience, data quality, and impact on clinical practice in real time, using the results to refine the registry as needed. Including these evaluation measures is expected to strengthen the protocol by providing accountability and opportunities for continuous improvement. Similar registry-based initiatives have highlighted the importance of such evaluation; for example, the recent ChaNoE cohort protocol explicitly aimed to standardize and validate follow-up and treatment criteria to facilitate consistent, high-quality data collection [19]. By integrating utility and validity assessments into Tropistry, we align with these best practices and add an extra layer of rigor to our platform’s implementation.

### Regulatory alignment and design rationale

Tropistry aligns with the European Medicines Agency (EMA) Guideline on Registry-Based Studies [26], incorporating core elements such as predefined objectives, disease-specific core datasets, and quality assurance through REDCap’s audit-enabled infrastructure. Its ambispective, modular structure supports long-term follow-up and pharmacovigilance, including documentation of serious adverse events. Although not currently intended for regulatory submission, it is designed with the methodological rigor required for future engagement with regulatory and health technology assessment (HTA) bodies—particularly for NTDs lacking formal trial data. Its design ensures utility for research, clinical care, and policy dialogue, and may trigger innovative research questions based on unusual observations for example.

### Embedded clinical guidance and real-time expert support

Tropistry integrates clinical algorithms and decision-support prompts directly within the CRF, delivers individualized treatment recommendations as data is entered, and offers real-time expert consultation. To our knowledge, this is the first initiative to combine these capabilities: clinicians entering case data receive tailored, up-to-date guidance within the CRF, and can immediately contact ITM experts via TROPmail or phone for case-specific advice. This combined model transforms the registry from a passive data collection tool into an active clinical support system, enhancing quality of care, promoting standardization, and facilitating continuous learning within and across centers.

### Interactive dashboard for dissemination and engagement

A key innovation is Tropistry’s interactive web-based dashboard, which provides participating sites with regularly updated summaries of epidemiological trends, treatment patterns, outcomes, and adverse events. These updates —generated from secure data exports—support transparency, enable clinical benchmarking, and promote feedback loops across sites. By offering aggregate visualizations without individual identifiers, the dashboard enhances accessibility and aligns with best practices in registry-based research.

### Care harmonization and equitable drug access

Tropistry may help reduce disparities in NTD management by documenting treatment practices across varied European settings. Access to essential therapies such as miltefosine for CL/MCL or praziquantel for schistosomiasis remains uneven due to national differences in reimbursement, availability, and importation procedures [27]. These barriers can result in delayed or unaffordable care, particularly outside specialized centers. As recently highlighted in a call for urgent action on the praziquantel shortage in Europe, access to essential NTD treatments cannot be taken for granted [49]. Initiatives like Tropistry are needed to generate evidence that supports harmonized strategies, informs regulatory and reimbursement decisions, and enables professional societies to advocate effectively for sustainable access to key therapies. In some systems, such data already influence reimbursement decisions for rare diseases [50,51].

### Strengths and limitations

Tropistry has several design strengths supporting its scalability and relevance: a modular structure for adding new disease-specific components; a clinical focus on diagnosis, treatment, and outcomes; an embedded expert guidance to promote standardized care; an interactive dashboard for timely data visualization; broad inclusion criteria that reflect real-world scenarios; consensus-based definitions for case classification and outcomes; and flexibility to accommodate local practices without compromising standardization; and incorporation of a built-in evaluation framework to monitor its feasibility, user engagement, data quality, and impact on clinical practice, enabling iterative improvements. By aligning with EMA-endorsed principles [26], Tropistry offers a pragmatic and adaptable platform with utility for clinical, regulatory, and public health applications. Compared to RCTs, it immediately captures broader variability in real-world care and treatment response, helping identify unmet needs and inform new research priorities [52].

Nonetheless, the platform faces limitations common to observational studies. Causal inference is not possible, and residual confounding must be considered. Data completeness may vary—especially for retrospective cases—due to site-specific workflows and diagnostic variation. In conditions with prolonged or stepwise recovery, outcome assessment may be delayed or incomplete, particularly where follow-up capacity is limited. More importantly, data quality largely depends on sustained clinician engagement, which may fluctuate over time without dedicated support. As it is about NTDs, no strong financial incentives can be expected for participating institutions. The major drivers would be scientific curiosity and the willingness to contribute to evidence-based clinical decision-making, while the main reward would be shared authorship in quality manuscript and policy briefs. Finally, selection bias is also likely in early phases, as initial participation will involve Belgian referral centers and travel/tropical clinics affiliated to TropNet. Although broader expansion is planned, early data may overrepresent specialized care settings. Widening participation will be key to enhancing representativeness and impact.

## Conclusion

Tropistry is a pragmatic, expert-driven registry designed to address persistent and apparently insolvable evidence gaps in the management of NTDs in nonendemic settings. Through standardized data collection, embedded clinical guidance, and robust governance, it provides a scalable framework to improve patient care, inform policy, and generate real-world evidence for rare and complex tropical infections. Continued expansion across diverse healthcare settings will be essential to realizing its full potential.

## Supporting information

S1 Protocol Tropistry Protocol v1.2. (PDF)

S1 File Acute Schistosomiasis module REDCap-based CRF. (PDF)

S2 File Cutaneous and mucocutaneous leishmaniasis module REDCap-based CRF. (PDF)

S1 Checklist Inclusivity in global research. (PDF)
