# Supplementary material for: Tropistry: A registry-based modular platform to improve care of neglected tropical diseases in nonendemic settings—Study protocol with two targeted conditions: acute schistosomiasis and cutaneous/mucocutaneous leishmaniasis
Source: PLoS One. 2025 Oct 30;20(10):e0335032. doi: 10.1371/journal.pone.0335032 (PMC12574823; doi:10.1371/journal.pone.0335032)
Supplement: S1 Protocol — (PDF) [file pone.0335032.s001.pdf]

INSTITUTE OF TROPICAL MEDICINE ANTWERP

Tropistry

Treatment Registry of Tropical Diseases in non-endemic settings

An international patient register to assess safety and clinical effectiveness of current treatments for selected tropical diseases in non-endemic settings

PROTOCOL v1.2

1. TABLE OF CONTENTS

1. Table of Contents 2
2. Protocol Summary 3
3. Background / rationale 5
4. Objectives 6
   4.1. Primary Objective 6
   4.2. Secondary Objectives 6
5. Methodology 7
   5.1. REGISTER Design 7
   5.2. study Period 7
   5.3. REGISTER Population 7
   5.4. Data collection AND DATA WORKFLOW 8
   5.5. Variables and Epidemiological Measurements 9
   5.6. Statistical Analysis 10
6. Budgetary and organizational Information 11
7. Ethical and Regulatory Considerations 12
   7.1. Privacy considerations 12
   7.2. PUBLICATIONS 13
8. THE CASE OF ACUTE SCHISTOSOMIASIS 14
   8.1. References for core text and acute schistosomiasis module 17
9. THE CASE OF CUTANEOUS AND MUCOCUTANEOUS LEISHMANIASIS 19
   9.1. References for CL/MCL module 23

2. PROTOCOL SUMMARY

Title	**Tropistry** - Treatment Registry of Tropical Diseases in the non-endemic setting
Primary Objective	To assess the safety and clinical effectiveness of current treatments for selected tropical diseases in the non-endemic setting
Register Design	Tropistry is an international, pseudonymized patient register designed to observe/document adverse events and clinical outcome related to current and future treatments of a set of tropical diseases, that are infrequently diagnosed, may cause severe complications, and for which optimal therapy is poorly established in the non-endemic setting.<br><br>This protocol describes the study procedure in Belgian (and other European) medical institutions where such patients could be diagnosed. Collected data will be centralized and analyzed at the Institute of Tropical Medicine (ITM), Antwerp, Belgium within its reference scientific duties.<br><br>For each selected tropical disease (see below), a specific case report form (CRF) will be designed with the REDCap software to capture relevant baseline, follow-up and outcome information regarding diagnosis and treatment. Participating centers will be invited to fill retrospectively the online CRF with pseudonymized data. During this process, state-of-the-art recommendations will be provided to non-specialized clinicians via messaging through the REDCap software and/or by contacting the ITM staff if felt necessary, with regular updates following new scientific knowledge.<br><br>Regular analysis of the data collected via Tropistry will provide harmonized key information that will serve as scientific background to further develop and refine evidence-based therapeutic recommendations on a set of tropical diseases in the non-endemic setting.
Selected tropical diseases	The following selected diseases will be studied in a phased approach. The selection is based on several considerations, including the rarity of these tropical diseases in the non-endemic setting (in migrants and/or travelers), on the severe morbidity they could cause, and on the paucity of robust data to guide decision to date.<br><br>Beside the generic information on the study, this protocol is limited to the first disease of the list here under, and amendments will be progressively submitted to include the additional ones, once the Tropistry will be more firmly established with a network of active reporting clinicians.<br><br>**1. Acute schistosomiasis**<br>2. Neurocysticercosis

	3. Cystic echinococcosis<br>4. Chagas disease<br>5. Cutaneous leishmaniasis<br><br>This list is not exhaustive and may evolve according to new emerging diseases or scientific advances. Whenever possible, collaboration with other existing or future similar initiatives will be explored at the Belgian or European level, to avoid any duplication of efforts.
Register Population	All patients diagnosed with one of the selected tropical diseases in any Belgian or European medical institution are eligible for inclusion. As this study is purely observational (no intervention beyond optimal standard of care), no written inform consent will be requested in institutions where an optout strategy is put in place (for epidemiological surveillance and secondary use of clinical data). In other institutions, a written informed consent will need to be obtained. In both situations' scenarios, participation to Tropistry will be notified in the medical file at inclusion (usually during one of the follow-up visits).<br><br>There is no prespecified sample size calculation, since most diseases are rare, and coverage is still uncertain. However, it is expected to reach at least 100 inclusions per disease.
Register Duration	Registry start date: 10/2024<br><br>Registry end date: none
Statistical Methods	The REDCap database will be analyzed on annual basis to statistically describe different variables as patient demographics, baseline clinical presentation, methods of diagnosis, staging of disease, (recommended) treatments, frequency and pattern of adverse events, complications and clinical outcome. Deviations with suggested treatments will also be captured (as well as reasons for alternative decisions). Association between specific treatment strategies and patient safety/clinical effectiveness will be analyzed using analysis of variance or Student's t-test for the continuous variables (or equivalent non-parametric tests for non-normally distributed variables Mann–Whitney U test or Kruskal-Wallis test) and the chi-square tests or Fisher's exact test for the categorical variables as well as multivariable regressions. Time to event analysis, by using Kaplan-Meier and Cox proportional hazard models will be performed to examine factors associated with specific outcomes.

3. BACKGROUND / RATIONALE

Over the past two decades, international travel has exponentially grown to reach almost 1.3 billion trips in 2023, back to the pre-pandemic levels (UN World Tourism Barometer, 2024). In parallel, migration from outside Europe also tends to re-increase after the pandemic, with an estimated 2.3 million arrivals in 2021, leading to more than 40 million non-European migrants living in Europe (World Migration Report, 2024). These unprecedented movements of population are associated with importation of pathogens from everywhere in the world, mainly originating from tropical low-resource countries. **Consequently, health professionals and health systems in Europe (and other Western countries) are increasingly confronted with non-endemic infectious diseases, for which they are not fully prepared**[1].

The clinical presentation and management of tropical diseases in non-endemic settings presents several specific challenges. First, there is a lack of epidemiological data and precise information on the burden. Second, clinical manifestations may be very different in the heterogeneous population of non-immune travelers and migrants compared to what is usually described in conventional textbooks on tropical/geographic medicine. Third, management may be negatively impacted by administrative, legal, financial or cultural hurdles limiting access to care for migrants, while on the other hand better diagnostics and treatments, when made available, can provide additional benefits compared to that in endemic low-resource settings. Finally, as mentioned, first-line clinicians are usually unfamiliar with tropical conditions, often leading to diagnostic delays, inadequate treatments and complex trajectories before specialized management can be offered[2]. **Consequently, management of rare tropical conditions in non-endemic settings is far from being optimal, and specific guidelines, when they exist, are little evidence-based and therefore poorly harmonized**[3].

All in all, there is pressing need to generate stronger evidence on the optimal management of such tropical diseases in travelers and migrants, for equity and cost saving purposes. However, as they are only sporadically seen, usually encountered in very different clinical practices, and managed within various disciplines, conducting randomized controlled trials is hardly feasible. **Documenting their presentation, evolution and outcome in a standardized way and under expert supervision is likely the best strategy to generate grade B evidence, the most reasonable level at reach for these diseases**[4]**.**

Accurate observational data on (1) the safety and (2) clinical effectiveness of the different treatments currently recommended, albeit often with very low/low level of evidence, will help identify the optimal practices, circumscribe the persisting knowledge gaps to be further researched, and elaborate more robust guidance for non-specialists from various disciplines[5]. **This is the very purpose of the ITM-initiated Tropistry project, based on an international patient register designed for selected tropical diseases with puzzling management in the non-endemic setting**.

4. OBJECTIVES

4.1. PRIMARY OBJECTIVE

To document the **safety** (frequency and pattern of adverse events) and **clinical effectiveness** (cure or stabilization rate at relevant timepoint) of current treatments for patients diagnosed with any of the selected tropical diseases (listed below*) in Belgian and European hospitals and travel clinics.

1. Acute schistosomiasis
2. Neurocysticercosis
3. Chagas disease
4. Cystic echinococcosis
5. Cutaneous leishmaniasis

*This selection is indicative and limited to parasitic diseases, based on the considerations described in the text, and may evolve according to the needs. Also, the extension of the registry to each disease will be progressively phased, following the proposed order.

4.2. SECONDARY OBJECTIVES

A. Assessment of epidemiological background

- To assess the sociodemographic characteristics of participants
- To assess the countries/regions of exposure and other epidemiological risk factors
- To assess the health seeking trajectory before definitive diagnosis

B. Assessment of quality of diagnosis

- To assess the way diagnosis was made, and the disease stagging if relevant
- To determine the proportion of direct microbiological diagnosis, compared to other indirect methods (serology, imaging…).

C. Documentation of clinical course and outcome

- To document the (successive) antimicrobial and adjunctive treatments administered and reasons for any interruption or change
- To observe whether the treatments are administered according to the supervised ITM's state-of-the-art and document the reasons for deviation
- To document the follow-up strategy in the real-life (frequency of visits, monitoring…)
- To determine the proportion of documented microbiological cure, in addition to the defined clinical cure (or stabilization/non-progression)

5. METHODOLOGY

5.1. REGISTER DESIGN

For this national and international multicentric effort, REDCap (Research Electronic Data Capture) will be used to offer harmonized disease-specific case report form (CRF), as well as state-of-the-art recommendations. REDCap is a GCP-compliant web-based metadata open-source software program available to academic institutional partners of Vanderbilt University. It protects data with secure web authentication, data logging and Secure Sockets Layer (SSL) encryption, and has features specifically designed for registry data management, including (i) customizable data collection forms, (ii) data validation and quality control checks, (iii) advanced reporting and export capabilities and iv) audit trails for tracking changes to data.

The Tropistry REDCap-based registers (one for each selected tropical disease) are being constructed to collect relevant demographic, diagnostic and clinical data using a predefined core dataset (Section 6.5 for full details). It is designed as a registry to harmonize as much as possible the selection of treatments (based on state-of-the-art review and expert advice) and suggested follow-up. Recruitment will include all patients diagnosed with one of the target diseases (see case definitions for each of them), either a treatment has to be initiated or not. Data will be entered directly into REDCap by an investigator from each participating site. Data will be stored pseudonymously at ITM.

As this study is purposed to capture as many cases as possible in all type of clinical settings, and to provide expert advice whenever necessary to colleagues of any discipline (not specialized in tropical diseases), barriers for inclusion will be minimized. Data collection will therefore be retrospective (provided therapy has been initiated since 2022, arbitrarily considered as post-pandemic period). In medical institutions where an opt-out strategy is in place for surveillance or scientific purposes, no formal written inform consent will be required, but inclusion in Tropistry will be presented communicated to the patient, with a notification in the patient file. Data will not be analyzed only if the eligible patient specifically does not consent. In other medical institutions, a written inform consent will need to be signed at whatever moment of the follow-up (with notification in medical file) for the data to be analyzed. Importantly, for every data transfer the entire data file is exported, so that follow-up data of patients can be each time easily incorporated.

5.2. STUDY PERIOD

Start date: September 2024 (for the first targeted disease)

End date: Not applicable as the protocol is designed as a continuous patient registry.

5.3. REGISTER POPULATION

For this register, there is no defined upper limit to the number of patients to be recruited. It is expected that at least 100 inclusions should be obtained per disease to allow some robust analysis leading to clinical recommendations.

Inclusion criteria

- Patients of any age
- Patients diagnosed with one of the target diseases according to pre-specified case definition
- Patients treated in a European / Belgian hospital or travel clinic
- Patients participating in a clinical trial (observational or interventional) can also be included.
- Patients orally consenting to participate (in sites with institutional opt-out strategy, with notification in file, or after a signed informed consent (possibly by a legal representative) for other sites)

Exclusion criteria

- Patients without basic medical information about diagnosis
- Patients not willing / not able (language barriers, …) to provide his/her oral or written consent

5.4. DATA COLLECTION AND DATA WORKFLOW

Tropistry is initially aimed at recruiting patients in Belgium, in any type of medical institutions, as part of the reference duties for which ITM is subsidized. It will be rapidly expanded to other European institutions, especially through the TropNet network, which has repeatedly expressed its interest in such an initiative.

At least one dedicated medical doctor per participating institution (as most cases will be complex), authorized via authentication in the hospital/travel clinic, enters the clinical data of each patient meeting the inclusion criteria.

Data entry will be done locally in the participating center within a self-explanatory REDCap-based CRF. As pseudonymized data are still considered personal data, the local investigator will be responsible for the protection of the data using their own instrument of security. It is recommended to enter the data after the initial and each follow-up visit, but this could be done also once the case is completed (at the end of the follow-up). As most target diseases require months or even years of follow-up, inclusion will be possible at any moment of the active follow-up period, although "early" inclusion will be encouraged to get the highest benefit of the accompanying expert guidance.

The administration and analysis of the Tropistry data at ITM will be limited to selected and named administrators/investigators who receive comprehensive training in the system before access is granted. Users need to log into the system with username and a secure password, including letters, numbers, and symbols, that will need to be changed regularly. All users will have a specific role which has predefined restrictions on what is allowed on the server (for example the user can only view and modify their own contributions).

Regular data-backup, hierarchic management of rights and authentication protocols of Tropistry protect data from unauthorized access and loss. Any data manipulation by users and administrators is logged in an audit trail, which allows complete data reconstruction. Server administration is performed at ITM by an expert data manager and includes regular updates of the servers, rigid security system

configuration, current virus and threat detection, and daily backups (on-site and off-site with secure storage).

5.5. VARIABLES AND EPIDEMIOLOGICAL MEASUREMENTS

Documentation of data at ITM will be pseudonymized, so that missing information could be retrieved by request to the treating physician.

The core “generic” dataset for all selected diseases includes baseline patient characteristics, details on care-seeking trajectories, clinical findings at presentation, diagnostic methods and ascertainment, information on stagging, and recommended / administered treatments normally during the whole follow-up period and final clinical/microbiological outcome whenever possible. CRF will be tailored to each specific disease, as the diagnosis and treatment may differ a lot. Due to potential evolving scientific research questions, adjustments of the register/template/dataset might be necessary throughout the study period, in particular if new target disease is identified or new treatments enter clinical practice. Minor adjustments will be collected and notified to the ethics committees; substantial changes will be submitted via an amendment.

The collected data will generically include the following core parameters after anonymization. A disease-specific CRF will be however each time elaborated as all target diseases have important clinical, diagnostic, and therapeutic specificities.

- **General Setup**
    - Epidemiological data on the case: principal investigators name, country, institution, level of care of the institution (e.g., primary care, secondary care)
    - Details on the entered case: Documentation in any other registry (yes/no, if yes DOI), case already published (yes/no)

- **Patient demographics and clinical features at time of diagnosis and initial treatment**
    - Age group, gender, height, weight, associated infections, relevant comorbidities, using Charlson Comorbidity Index (CCI) and in particular immunosuppressive conditions and/or treatments
    - Pre-diagnosis trajectory
    - Clinical signs and symptoms of infection leading to diagnosis and treatment
    - Relevant laboratory parameters (CRP, eosinophilia…)

- **Details on the diagnostic method / case ascertainment**
    - Correspondence to pre-specified case definitions (as composite references standards are often needed for parasitic diseases
    - Information on disease stagging when relevant
    - Details on direct microbiological diagnosis (whenever available)

- **Initial and second line (if any) treatment and outcome**
    - Recommended treatment (in the state-of-the-art section or national guidelines
    - Administered antiparasitic and adjunctive treatment (in case different of recommended, request to explain the reason for deviation)
    - Description and timing of drug-related adverse event if any

- Need of treatment interruption and reason (availability, adverse event, disease progression…)
    - Laboratory changes (Electrolytes, Creatinine, liver function panel, Platelet count, red blood cell count, hemoglobin, white blood cell count, neutrophils, leukocytes, eosinophil count)
    - Frequency and timing of follow-up visits with specific findings, if any
    - Treatment response regarding clinical features (disease progression vs. stabilization vs. cure), with day of assessment
    - Treatment response regarding microbiological outcome: eradication, reduction of the parasitic load, with day of assessment
    - If inpatient stay: days on normal ward, intermediate care, intensive care unit total, as a marker of morbidity
    - In case of death, cause of death
- Additional information on the case can be entered as a narrative

5.6. STATISTICAL ANALYSIS

The international register will be analyzed annually to produce descriptive statistics on different variables including patient safety, patient demographics, details on diagnosis and administered treatments. Clinical outcomes and the follow-up of patients will also be assessed. Association between specific treatment strategies and patient safety/patient-related parameters will be analyzed using analysis of variance or Student's t-test for the continuous variables (or equivalent non-parametric tests for non-normally distributed variables Mann–Whitney U test or Kruskal-Wallis test) and the chi-square tests or Fisher's exact test for the categorical variables as well as multivariable regressions. Time to event analysis, by using Kaplan-Meier and Cox proportional hazard models will be performed to examine factors associated with the clinical outcomes. All statistical analyses will be performed using STATA, Python, and/or R statistic software and will be performed by trained personnel (epidemiologist, statistician) using current methods of analysis.

6. BUDGETARY AND ORGANIZATIONAL INFORMATION

Tropistry has been created by the Department of Clinical Sciences of ITM, within the convention with the National Insurance System (NIS) that ITM must serve as national reference center for care of tropical diseases in travelers and migrants in Belgium. The project will be coordinated by ITM, and a scientific board with travel/migration experts of other Belgian/European institutions will be created to monitor and orientate this initiative.

The REDCap design and maintenance will be covered by the NISs convention, as well as all ITM expert advice which will be provided for the state-of-the-art supervision of included cases. There is no dedicated funding for the inclusion and documentation of the cases (no case fees) in Belgium or elsewhere. Each participating site will have to identify one or two contact persons who will be responsible for data entry in the common e-CRF.

Expansion to other European institutions will be offered for free to the (TropNet) partners, who will also have to activate and maintain Tropistry at their own cost. Several centers have already expressed their interest to join the project, at least for the first targeted disease (acute schistosomiasis).

Once a year, an activity report (newsletter) will be provided to all participating centers and regular scientific online meetings and/or presentations will be organized to provide feedback and discuss corrections or reorientations if necessary.

7. ETHICAL AND REGULATORY CONSIDERATIONS

All register procedures are liable to Good Epidemiological Practice requirements as well as Belgian and European legislation. Where applicable, the investigators will uphold and guarantee the standards of Good Clinical Practice in their conduct. No interventions will take place throughout this register. All participants (and/or parents) will be either recruited through institutional optout umbrella for surveillance and secondary use and clinical data, in centers where such a strategy is in place, or via formal written informed consent form obtained during follow-up period before any data can be entered.

The principal coordinating investigator and scientific board state to have no applicable conflict of interest with any of the interventions, devices, or drugs that may or may not be documented by participating investigators in their patient care.

7.1. PRIVACY CONSIDERATIONS

The following measures will be taken to rule out risk of re-identification for patients registered in Tropistry :

1. The dataset of the international register will not contain any directly identifying information, i.e., no name, birth date, place of residence (just country of residence), insurance company or any identifiers linked to the patient record at the participating center.

2. No specific dates will be encoded. The day of initial diagnosis will remain unknown to the register, only the year will be specified. All major events will be recorded as days from diagnostic ascertainment (day 0)

3. Almost all data will be collected in categories, e.g. age groups, lab values etc., making it almost impossible to link the data with other sources, e.g., by performing a reverse database search in the hospital information system.

4. All text items will be screened for potentially identifying information. Accidental breaches of anonymity will be reported to the responsible investigator and permanently deleted from the database. Relevant and shareable information will be moved into a structured data item.

Taking these considerations into account, the risk of a full data breach can be considered very low. Tropistry will be maintained in the ITM REDCap server. For data clearance and optimization pipelines as well as complete analyses of the uncensored dataset, the full dataset may be moved to certified secure network areas. Such processing and analysis will be performed by a small group of authorized persons supervised by the principal coordinating investigator. All data will be stored for at least 10 years following the end of the registry (in case of termination).

7.2. PUBLICATIONS

It is intended to publish the results of study analyses in peer-reviewed journals and to present them at scientific conferences. All publications will be performed under the name of the Tropistry Study Groups (composed of ITM study staff + at least one representative of each site which would have included at least one case), which may be different for each target disease.

8. THE CASE OF ACUTE SCHISTOSOMIASIS

Background and rationale for selecting this condition

Schistosomiasis is a tropical parasitic disease caused by blood-dwelling fluke worms of the genus *Schistosoma* (Phylum Platyhelminthes, Class Trematoda), characterized by a complex life cycle[6]. The infection is amplified by freshwater mollusks acting as intermediate hosts and acquired through infective cercariae penetrating the skin. Competent vectors are found mainly in Africa, but also in the eastern part of South America, and to a lesser extent in Arabia, China, and Southeast Asia. In endemic countries, transmission occurs via recreational or occupational contacts with surface water, and repeated exposure is usually necessary to acquire a significant parasite burden, responsible for substantial chronic morbidity if left untreated. After a migration and maturation phase in tissues, the adult human schistosomes, finally localized in specific venous plexus, start laying eggs that may be trapped in neighboring tissues. This phenomenon produces essentially hepato-intestinal disease (*Schistosoma mansoni, Schistosoma japonicum, Schistosoma intercalatum, Schistosoma mekongi*) and urogenital disease *(Schistosoma haematobium*), initially characterized by chronic granulomatous inflammation, followed by fibrotic scarring usually causing irreversible damages.

Schistosomiasis is among the most frequent parasitic diseases diagnosed in European travel clinics, after malaria[1]. It is also among the top three neglected tropical diseases diagnosed in migrants from endemic countries[7], affecting up to 25% of migrants originating from sub-Saharan Africa (as assessed by serology[8]). In the non-endemic setting, schistosomiasis may present with acute or chronic manifestations, defined according to a somehow arbitrary time span of three months after infection[9].

Chronic schistosomiasis (defined as infection lasting for more than 3 months) is mainly seen in migrants from endemic areas, and to a lesser in travelers and expatriates. It can remain fully asymptomatic for a long period, but may also cause at any moment of the evolution a wide variety of early or late hepato-intestinal, urogenital and - rarely -ectopic complications, ranging from abdominal pain, chronic diarrhea, hematochezia, hematuria, genital pain, (…) to portal hypertension, obstructive uropathy, bladder carcinoma or neurological compression, (…). Chronic schistosomiasis is diagnosed in more than 20% of the migrants born and raised in Africa.

Acute schistosomiasis (the first condition we want to study in Tropistry) refers to a systemic hypersensitivity reaction directed against the maturing schistosomulae, migrating juvenile and adult worms and deposited eggs[10]. This syndrome has been first recognized in Japan in the beginning of last century and is also called “Katayama fever” (or better “Katayama syndrome”, as fever is not always present) once symptoms become clinically apparent (this is not always the case). Symptoms have only been described in people infected for the first time with schistosomiasis, i.e. in nonimmune travelers. They may occur from three weeks to three months after infection with one of the human schistosome species, and include low to medium grade fever, a non-productive cough sometimes with dyspnea, abdominal pain, and/or diarrhea. Patchy nodular infiltrates are occasionally seen on chest X-ray. An urticarial rash and/or angio-oedema may precede fever, which is almost always associated with eosinophilia (eosinophil count > 500/µL). The underlying pathophysiological mechanism remains unclear. Most patients however recover spontaneously after 2 to 10 weeks, and only exceptionally require hospitalization. An important proportion of them will however become chronically infected, with a sizable risk of delayed complications whenever the egg burden is sufficient.

The risk of developing Katayama syndrome after exposure is only mildly correlated with the intensity of infection, and probably depends on host related immune reactions. Katayama syndrome is a frequent diagnosis in travelers and expatriates from nonendemic countries recently exposed to cercariae in fresh water. Travelers contract schistosomiasis almost exclusively in sub-Saharan Africa (up to 2% of all fever cases after a stay in this region[11]), often in families or group clusters, mainly after water contact for recreational purposes (bathing, scuba diving, water skiing, sailing, and rafting). In that population, it is almost exclusively associated with *S. mansoni* and *S. haematobium* infection, and only exceptionally with other species. After exposure, the schistosomiasis infection rate may reach nearly 100% in nonimmune travelers and the risk of developing Katayama syndrome after primary infection may exceed 50%[12]. The diagnosis has to be strongly suspected when eosinophilia is found during the workup for an exposed traveler with protracted fever and other nonspecific symptoms (cough, abdominal pain…) of unclear origin[13]. Exceptionally more severe complications such as myocarditis, cerebral vasculitis and ischemic colitis are seen during the acute phase of the disease[14].

Correctly diagnosing Katayama syndrome in an early stage is very challenging[10]. At the start of the feverish episode, a raised eosinophilia is almost always present but may be overlooked. At that moment it is difficult to confirm the diagnosis, as it takes time (4 to 12 weeks after cercarial penetration) for antibody tests (based on adult worm antigen or egg antigen) to become positive or for eggs to appear in feces or urine. Ova deposition and excretion in feces or urine happen at earliest from the 6th week (35 days) after infection. From large series in imported pathology, it has been observed that a possible or proven contact with fresh water in sub-Saharan Africa a few weeks prior to the onset of fever, combined with a raised eosinophilia is already specific enough to strongly suspect the diagnosis and to initiate empiric treatment. In practice, the syndrome is seldom recognized by primary health care providers who are not familiar with tropical pathology. By the time patients are referred to a travel clinic, evidence of schistosomiasis is found in the majority, mainly by serum antibody detection. Two novel diagnostics, based either on molecular techniques (PCR) or on antigenic detection (circulating anionic antigen [CAA], become positive much earlier than the conventional methods (urine/feces microscopy and antibody-based serology), but remain hardly available in clinical practice due to their cost and sophistication[15].

The treatment of acute schistosomiasis, either symptomatic (Katayama syndrome) or not, is even more challenging than its diagnosis. As the symptoms are mainly driven by the hypersensitivity reaction, corticosteroids need to be administered in diseased patients, but the optimal timing, dosage and duration are poorly established in the absence of randomized trials. It makes sense to associate the (currently unique) antischistosomal drug, praziquantel, to kill off the already matured schistosomes, but this treatment is largely ineffective against juvenile worms, partly responsible of the symptoms. Therefore, most of the time experts do not recommend praziquantel in the early phase of the acute infection, arbitrarily established within 6 weeks after infection, while it should/could be administered later on. But here again and for the same reason, the optimal timing and dosage of praziquantel to completely clear the infection are unknown, and many different regimens are in place according to local experience and expert-based guidelines. In addition, the interactions between corticosteroids and praziquantel are poorly understood but it appears that the blood concentration of the latter drug substantially decreases when co-administered with corticosteroids. Finally, the administration of praziquantel in asymptomatic, or mildly symptomatic, infected individuals may provoke in more than 50% of cases a serious clinical exacerbation (by antigenic release). Overall, there is no evidence regarding the optimal therapy of acute schistosomiasis[5], and a wide variety of regimens are proposed

even in specialized setting, as highlighted in 2019 by a survey of the European network of travel clinics, TropNet (unpublished data).

Specific aspects of the treatment registry on acute schistosomiasis

For this condition, participation to the registry will be proposed by the ITM team in priority to the main established university-based travel clinics of Belgium, as suspected cases of acute schistosomiasis are soon or late referred to them for the specialized diagnostic workup and complex therapeutic decisions. As only a few cases are diagnosed in specialized settings each year (about 5 in average at ITM), other European travel clinics linked to TropNet will be approached in a second step to allow rapidly increasing the number of inclusions, in other to obtain a robust sample size within a reasonable period (of several years). Most Belgian travel clinics and several European TropNet partners have already expressed interest to join this scientific effort, including the clinical sites of ISGlobal in Barcelona, of Sacro-Cuore Don Calabria Ospedale in Negrar or the University of Bordeaux.

Like for the other tropical conditions which will be targeted by Tropistry, the REDCap-based CRF for acute schistosomiasis will be tailored to capture the specificities of diagnosis and management of this very condition. Case definition for inclusion in the register will be based on recently released recommendations of an international panel of experts for disease categorization and staging, to allow harmonized research[16]. Thus, cases will be classified into confirmed, probable, or possible acute schistosomiasis according to epidemiological, microbiological and clinical data (see Annex 1 for complete case definitions). Sections will be specifically designed to assess the epidemiological risks, quality of diagnosis and robustness of monitoring. The list of data which will be collected during the study is available in Annex 2.

As previously explained, Tropistry will not only be a data collection tool, but also a platform to promote the best clinical practice, based on the limited available evidence or local experience. Instructions for management will be suggested by the ITM team and other experts, through short texts appearing at the beginning of each section (for diagnosis, treatment, and follow-up). Also, a panel of ITM experts could be reached at any moment via secure mail (TROPmail@itg.be), to discuss any difficult decision.

These "recommendations" will of course evolve with the scientific advances and new knowledge (included that generated by Tropistry). It is important to highlight that each center may propose different therapeutic regimens, according to local/national guidelines. As of June 2024, based on the experience at ITM (current state-of-the-art at ITM; manuscript about a recent cluster being submitted), we will recommend in Tropistry the following therapeutic approach:

- In case of symptomatic acute schistosomiasis,
  - If diagnosis earlier than 6 weeks after exposure/infection: only methylprednisolone 0.5 mg/kg/day for 3 days, to be prolonged/repeated if unsatisfactory control of inflammatory symptoms
  - If diagnosis made later than 6 weeks after infection: praziquantel 40 mg/kg single dose, followed after 2-4 hours by methylprednisolone 0.5 mg/kg/day for 3 days, to be prolonged/repeated if unsatisfactory control of inflammatory symptoms (note that this interval allows the concentration peak of praziquantel within 1-2 hours after administration, with no drug interaction)

- In case of asymptomatic acute schistosomiasis (for example within a cluster of cases)
    - Diagnosis earlier than 6 weeks after infection: abstention
    - Diagnosis later than 6 weeks after infection: praziquantel 40 mg/kg single dose followed after 2-4 hours by methylprednisolone (to prevent any clinical exacerbation)

All patients with acute schistosomiasis (either symptomatic or not) will receive a second 40 mg/kg single-dose course of praziquantel at week 12 after infection (to clear all juvenile worms which have meanwhile become adults). Here there is a large consensus between experts.

As already highlighted, the purpose is to make Tropistry as accessible as possible for busy clinicians confronted with any of the target diseases. For pragmatic reasons, inclusion and data entry will be retrospective (with reasonable delay of 6 months after initial diagnosis), and considered as secondary use of clinical data for institutions where an optout strategy is in place. This will be the case with most partners for this first phase of the project focusing on acute schistosomiasis. In other settings, a formal written inform consent will be requested for each included patient.

8.1. References for core text and acute schistosomiasis module

1. Grobusch MP, Weld L, Goorhuis A, et al. Travel-related infections presenting in Europe: A 20-year analysis of EuroTravNet surveillance data. The Lancet Regional Health - Europe 2021;1.

2. Comelli A, Riccardi N, Canetti D, et al. Delay in schistosomiasis diagnosis and treatment: A multicenter cohort study in Italy. J Travel Med 2020;27(1).

3. Cucchetto G, Buonfrate D, Marchese V, et al. High-dose or multi-day praziquantel for imported schistosomiasis? A systematic review. J Travel Med. 2019;26(7).

4. Baynam G, Hartman AL, Letinturier MC V., et al. Global health for rare diseases through primary care. Lancet Glob Health. 2024;12(7):e1192–9.

5. Comelli A, Genovese C, Gobbi F. Schistosomiasis in non-endemic areas: Italian consensus recommendations for screening, diagnosis and management by the Italian Society of Tropical Medicine and Global Health (SIMET). Infection 2023;

6. Colley DG, Bustinduy AL, Secor WE, King CH. Human schistosomiasis. In: The Lancet. Elsevier B.V.; 2014. p. 2253–64.

7. Zammarchi L, Gobbi F, Angheben A, et al. Schistosomiasis, strongyloidiasis and Chagas disease: The leading imported neglected tropical diseases in Italy. J Travel Med 2020;27(1).

8. Greenaway C, Asundi A, Beliavsky A, et al. Prevalence of strongyloidiasis and schistosomiasis among migrants: a systematic review and meta-analysis [Internet]. 2019. Available from: www.thelancet.com/lancetgh

9. Gobbi F, Tamarozzi F, Buonfrate D, van Lieshout L, Bisoffi Z, Bottieau E. New Insights on Acute and Chronic Schistosomiasis: Do We Need a Redefinition? Trends Parasitol. 2020;36(8):660–7.

10. Jauréguiberry S, Paris L, Caumes E. Acute schistosomiasis, a diagnostic and therapeutic challenge. Clinical Microbiology and Infection. 2010;16(3):225–31.

11. Bottieau E, Clerinx J, Schrooten W, et al. Etiology and Outcome of Fever After a Stay in the Tropics [Internet]. Available from: www.archinternmed.com

12. Clerinx J, Van Gompel A. Schistosomiasis in travellers and migrants. Travel Med Infect Dis 2011;9(1):6–24.

13. Bottieau E, Clerinx J, de Vega MR, et al. Imported Katayama fever: Clinical and biological features at presentation and during treatment. Journal of Infection 2006;52(5):339–45.

14. Bonnefond S, Cnops L, Duvignaud A, et al. Early complicated schistosomiasis in a returning traveller: Key contribution of new molecular diagnostic methods. International Journal of Infectious Diseases 2019;79:72–4.

15. Cnops L, Huyse T, Maniewski U, et al. Acute Schistosomiasis with a Schistosoma mattheei × Schistosoma haematobium Hybrid Species in a Cluster of 34 Travelers Infected in South Africa. Clinical Infectious Diseases 2021;72(10):1693–8.

16. Tamarozzi F, Mazzi C, Antinori S, et al. Consensus definitions in imported human schistosomiasis: a GeoSentinel and TropNet Delphi study. Lancet Infect Dis. 2024 Oct;24(10):e627-e637.

9. THE CASE OF CUTANEOUS AND MUCOCUTANEOUS LEISHMANIASIS

Background and rationale for selecting this condition

Cutaneous leishmaniasis (CL) and mucocutaneous leishmaniasis (MCL) are forms of leishmaniasis, a vector-borne parasitic disease caused by protozoan parasites of the genus *Leishmania.* CL is the most frequent disease presentation worldwide, with approximately 1–2 million new cases occurring annually[1.] CL is endemic in nearly 100 countries, and most cases occur in tropical and subtropical regions (over 90% are reported from countries such as Afghanistan, Brazil, Iran, Peru, Saudi Arabia, Algeria, and Syria)[2]. Mucocutaneous leishmaniasis (MCL), a less common form involving mucosal tissue, is nonetheless a serious and disfiguring condition with significant morbidity. Leishmaniasis is classically divided into Old World and New World forms, according to the species causing the disease and their geographical distribution. Old World species, such as *L. infantum*, *L. major*, and *L. tropica*, are found in Europe, Asia, and Africa, whereas New World species—mainly from the *Viannia* subgenus like *L. braziliensis*, *L. guyanensis*, and *L. panamensis*—are endemic to Central and South America and are more frequently associated with mucosal complications. Large areas of Southern Europe are endemic for leishmaniasis, but the CL and MCL caseload increases in all European countries following international travel and migration. A 20-year surveillance from the GeoSentinel network recorded 955 patients with travel-acquired CL/MCL[333] highlighting that this disease complex represents an important health issue in returning travelers and migrants, with some theoretical risk of secondary spread in European regions were competent vectors (i.e. Phlebotomus, or sand fly's) are present. Cutaneous leishmaniasis is regularly seen in European tropical clinics, ranking among the most frequent travel-related skin infections. Supporting this, in a large European cohort study by the *LeishMan* network, 459 patients with CL acquired in 47 different countries were analyzed over a 13-year period. Among them, 60% had acquired the infection in Old World regions (notably Spain, Syria, and Morocco), and 40% in the New World (mainly French Guiana, Peru, and Costa Rica)[4].

CL is characterized by skin lesions that can range from small, localized sores to large nodules or plaques. These lesions typically develop on exposed areas of the skin within weeks to months after infection. The sores can evolve from erythematous papules or nodular plaques to open ulcers with raised borders and central craters, often covered with crust or scales. While these lesions are usually painless, they can become painful if secondarily infected by bacteria. Without treatment, most sores eventually heal but can last for months or years and typically result in scarring. MCL occurs when the parasites spread by contiguity from the skin to mucosal surfaces, primarily affecting the nose, mouth, and throat, or through infectious metastases that may reactivate years after the primary cutaneous lesions have spontaneously healed. Symptoms include nasal congestion, epistaxis, and chronic mucosal inflammation or ulceration. In advanced cases, it can lead to severe destruction of the nasal septum, larynx, and pharynx. MCL is most often due to the latter metastatic mechanism, usually associated with the *Viannia* subgenus, which includes *L. braziliensis* and *L. guyanensis* complexes [5–7], and is exclusively endemic in Central and South America. MCL is exceptionally reported in Southern Europe, and East Africa, or in immunocompromised patients.

Diagnosis relies on parasitological confirmation through microscopic identification of amastigotes on Giemsa-stained smears, culture of lesion material, or molecular testing. PCR, particularly targeting ITS1

or HSP70 genes, is the most sensitive method and is routinely used at reference centers, often allowing species identification. Although rarely specific, histopathology may support diagnosis and rule out differential diagnoses such as subcutaneous mycoses or infection with atypical mycobacteria. Note that species identification has gained considerable importance, as treatment choice and prognosis differ significantly between species (see paragraph below).

Cutaneous leishmaniasis can be managed with either local or systemic therapy, depending on the clinical presentation and the infecting species. Local therapies used for simple CL may consist of intralesional pentavalent antimonials, cryotherapy, topical paromomycin and local heat therapy. For simple CL due to species like *L. major* or *L. mexicana*, local therapy is often sufficient. Patients should be monitored for response; if lesions do not show healing within a few months, escalation to systemic therapy might be needed. The main systemic therapies used for complex or refractory CL, and for MCL usually consist of pentavalent antimonials (meglumine antimoniate or sodium stibogluconate), oral miltefosine and liposomal amphotericin B. Other systemic options are: pentamidine isethionate, azole antifungals (see Annex 3 for detailed general and specific treatment by etiological species based on the *LeishMan* group guidelines). However, treatment of CL/MCL remains controversial and heterogeneous. Over the past decades, several national and international expert groups (e.g. *LeishMan* consortium in Europe, IDSA/ASTMH in North America) have published therapeutic guidelines for leishmaniasis in travelers [7,8]. While these guidelines are aligned on many points, they also show discrepancies because evidence for optimal treatment is limited – many recommendations are based on small case series, often in endemic areas with immune populations, or expert opinion rather than robust clinical trials[1.] As a result, clinical practice varies greatly between centers and countries. For instance, whether to treat certain Old World CL cases with local therapy or systemic therapy can differ by guideline, and approaches to New World CL to prevent (late) mucosal spread are not uniform. This is further complexified by the increasing recognition that species matters in terms of treatment response. This paucity of data and heterogeneous use of species diagnosis lead to uncertainty about which treatments achieve the best outcomes for the large variety of clinical scenarios. In addition, some therapies (e.g. antimonials) may have significant toxicity while the success rate of newer or repurposed compounds appears highly variable and setting-dependent. For example, a recent European retrospective analysis found that pentavalent antimonial therapy yielded about a 76% cure rate overall (with higher success when given intralesionally), whereas liposomal amphotericin B (the emerging standard of care because of its safety) had only ~44% cure rate in *L. tropica* infections [9,] highlighting the need to better understand which treatments work for which species.

Specific aspects of the treatment registry

Participation to this treatment registry of CL/MCL will be proposed by the ITM team in priority to the main established university-based travel clinics and dermatology practices in Belgium, as suspected cases are soon or late referred to these facilities for the specialized diagnostic workup and therapeutic decisions. However, the number of cases seen in Belgium remains low.

According to a retrospective study conducted at the ITM in Antwerp between 2010 and 2018, only 147 cases of CL/MCL were identified, averaging fewer than 20 cases per year[10]. Despite a modest increase in diagnoses over time[10], to ensure a sufficient and representative inclusion of cases across different *Leishmania* species, the registry will be progressively opened to other European travel clinics affiliated

with TropNet or the *LeishMan* network to allow rapidly increasing the number of inclusions and obtain some conclusive sample size for the different species.

Like for the other tropical conditions which will be targeted by Tropistry, the REDCap-based CRF for CL and MCL will be tailored to capture the many and rather complex specificities of management. Sections will be specifically designed to assess the epidemiological risks, quality of diagnosis and robustness of monitoring as well as to appropriately evaluate the final clinical outcome. The list of data which will be collected during the study is available in Annex 4.

As previously explained, Tropistry will not only be a unidirectional data collection tool, but also a platform to promote in return the best clinical practice, based on the limited available evidence or local experience. Instructions for management will be suggested by the ITM team and other experts, through short texts appearing at the beginning of each section (for diagnosis, treatment, and follow-up). Also, a panel of ITM experts could be reached at any moment via secure mail (TROPmail@itg.be), to discuss any difficult decision.

These "recommendations" will of course evolve with the scientific advances and new knowledge (included that generated by Tropistry). It is important to highlight that each center may propose different therapeutic regimens, according to local/national guidelines. The decision for the type of treatment of CL should mainly rely on (1) the *Leishmania* species, (2) the clinical aspects of the lesions, (3) the host immunity and local drug availability and experience. A new classification of CL is emerging in international literature, with the distinction between "simple CL" and "complex CL", although there are still some slight discrepancies between groups of experts[7,8.] To address these discrepancies, we follow the approach used by Vandeputte et al. in a recent retrospective study about leishmaniasis management in Belgium[10] adapting the classifications proposed by the IDSA and the *LeishMan* group to ensure that all cases could be consistently categorized as either simple or complex. As noted in that study, the two guidelines present certain gaps and are not fully congruent; therefore, for study purposes, an adapted classification was applied to overcome these inconsistencies and allow for standardized case assignment (see next table).

Definition of complex cutaneous leishmaniasis according to IDSA, the LeishMan group and the one proposed by Vandeputte et al.

Criteria	IDSA[7a]	*LeishMan* group[8]	Vandeputte et al.[10]
**Size**	>4 cm	>3 cm	>4 cm
**Number of lesions**	>4	>3 or >1[b]	>4
**Location**	Face, ears, eyelids, lips, fingers, toes, joints, genitalia	"Delicate location"/ 'cosmetically disfiguring'	Ears, eyelids, nose, lips, joints, fingers, toes
**Mucosal involvement**	Yes	Yes	Yes
**Immunosuppression**	Yes	Yes	Yes
**Failure to local treatment**	Yes	Yes	Yes
**DCL/LR**	Yes	Unclear	Yes

**Subcutaneous nodules**	Yes	Unclear	Yes
**Lymphatic spread**	Yes	Yes	Yes
**Infection in Bolivia**	Unclear	Yes	Unclear

DCL: diffuse cutaneous leishmaniasis, LR: Leishmania recidivans

[a]For the IDSA guidelines there are cases where the simple and complex is not mutually exclusive; e.g. 1-4 cm size, and 2-4 lesions

[b]A distinction is made between Old-World species (with the addition of *L. mexicana* from the New-World)*,* for which more than 3 lesions are considered to be complex, while for New-World species (with the exception of *L. mexicana*) having multiple lesions is sufficient to qualify as complex cutaneous leishmaniasis.

In general, for simple CL, observation/simple wound care or local treatment are sufficient, while systemic therapy should be considered for complex CL[4,7]. Of note there are still slight differences between guidelines about some criteria (especially regarding the size and number of lesions) but treatment decisions based on ”common sense“ will be suggested whenever discrepancies occur. In all cases, confirmation of the diagnosis is important, and whenever feasible, PCR-based species identification will be recommended because it increasingly guides therapy choices[10,11.] However, species typing might not be available everywhere; in such cases, clinical judgment and knowledge of the probable region of infection will guide management.

Follow-up, treatment failure, and scarring assessment have been standardized in the registry following recent international recommendations[12]. Clinical response is now evaluated at predefined time points: Day 42 and Day 90 (initial cure assessments), 6 months (final cure), and optionally at 9–12 months to detect relapse (see Figure). Treatment failure is defined as <50% re-epithelialization or flattening by Day 42, or <100% re-epithelialization or flattening by Day 90, in line with the consensus reached in harmonized clinical trial methodologies for cutaneous leishmaniasis. In addition, scar outcomes are systematically assessed using a standardized grading (from no visible scar to severe disfigurement or functional limitation) and classified by type (e.g. flat, atrophic, hypertrophic, keloid, pigmentary alteration). This approach enhances comparability across sites and aligns with WHO/TDR and DNDi guidance for clinical evaluation of CL.

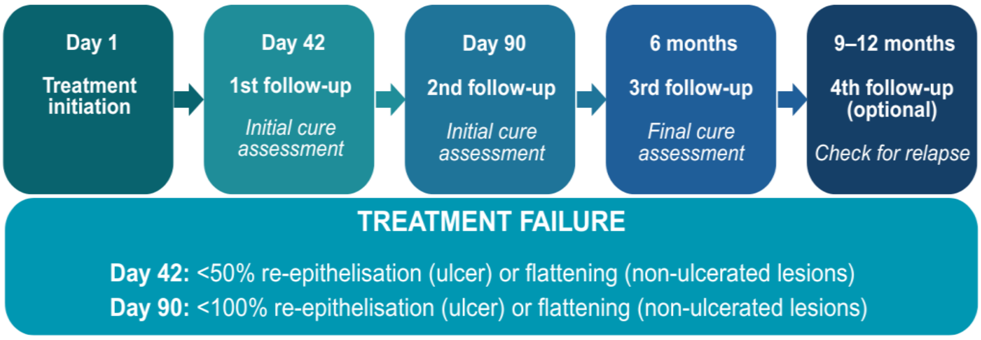

Based on *Olliaro P et al. Harmonized clinical trial ethodologies for localized cutaneous leishmaniasis and potential for extensive network with capacities for clinical evaluation. PLoS Negl Trop Dis 2018 12(1): e0006141.*

As already highlighted, the purpose is to make Tropistry as accessible as possible for busy clinicians confronted with any of the target diseases. For pragmatic reasons, inclusion and data entry will be retrospective (with reasonable delay of maximum 6 months after initial diagnosis), and considered as secondary use of clinical data for institutions where an optout strategy is in place. In other settings, a formal written inform consent will be requested for each included patient.

9.1. References for CL/MCL module

1. de Vries, H. J. C. & Schallig, H. D. Cutaneous Leishmaniasis: A 2022 Updated Narrative Review into Diagnosis and Management Developments. *Am J Clin Dermatol* **23**, 823 (2022).
2. Kurt, Ö., Özbilgin, A., Petersen, E. & Ergönül, Ö. An Update on the Imported Cutaneous Leishmaniasis in Europe. *Infectious Diseases & Clinical Microbiology* **5**, 59 (2023).
3. Boggild, A. K. *et al.* Cutaneous and mucocutaneous leishmaniasis in travellers and migrants: a 20-year GeoSentinel Surveillance Network analysis. *J Travel Med* **26**, taz055 (2019).
4. Guery, R. *et al.* Clinical diversity and treatment results in Tegumentary Leishmaniasis: A European clinical report in 459 patients. *PLoS Negl Trop Dis* **15**, e0009863 (2021).
5. Leishmaniasis, Cutaneous | CDC Yellow Book 2024. https://wwwnc.cdc.gov/travel/yellowbook/2024/infections-diseases/leishmaniasis-cutaneous.
6. Handler, M. Z., Patel, P. A., Kapila, R., Al-Qubati, Y. & Schwartz, R. A. Cutaneous and mucocutaneous leishmaniasis: Clinical perspectives. *J Am Acad Dermatol* **73**, 897–908 (2015).
7. Aronson, N. *et al.* Diagnosis and Treatment of Leishmaniasis: Clinical Practice Guidelines by the Infectious Diseases Society of America (IDSA) and the American Society of Tropical Medicine and Hygiene (ASTMH). *Am J Trop Med Hyg* **96**, 24 (2017).
8. Blum, J. *et al.* LeishMan recommendations for treatment of cutaneous and mucosal leishmaniasis in travelers, 2014. *J Travel Med* **21**, 116–129 (2014).
9. Glans, H. *et al.* Treatment outcome of imported cutaneous leishmaniasis among travelers and migrants infected with Leishmania major and Leishmania tropica: a retrospective study in European centers 2013 to 2019. *International Journal of Infectious Diseases* **122**, 375–381 (2022).
10. Vandeputte, M. *et al.* Epidemiology, clinical pattern and impact of species-specific molecular diagnosis on management of leishmaniasis in Belgium, 2010–2018: A retrospective study. *Travel Med Infect Dis* **38**, 101885 (2020).

11. van Griensven, J. *et al.* Treatment of Cutaneous Leishmaniasis Caused by Leishmania aethiopica: A Systematic Review. *PLoS Negl Trop Dis* **10**, e0004495 (2016).

12. Olliaro, P. *et al.* Harmonized clinical trial methodologies for localized cutaneous leishmaniasis and potential for extensive network with capacities for clinical evaluation. *PLoS Negl Trop Dis* **12**, e0006141 (2018).
