# Supplementary material for: Tropistry: A registry-based modular platform to improve care of neglected tropical diseases in nonendemic settings—Study protocol with two targeted conditions: acute schistosomiasis and cutaneous/mucocutaneous leishmaniasis
Source: PLoS One. 2025 Oct 30;20(10):e0335032. doi: 10.1371/journal.pone.0335032 (PMC12574823; doi:10.1371/journal.pone.0335032)
Supplement: S1 File — (PDF) [file pone.0335032.s002.pdf]

Demographics

Record ID	______________________
Sex at birth	◯ Male<br>◯ Female<br>◯ Other
Year of birth	______________________

Country of birth

- ◯ Unknown
- ◯ Afghanistan
- ◯ Åland Islands
- ◯ Albania
- ◯ Algeria
- ◯ American Samoa
- ◯ Andorra
- ◯ Angola
- ◯ Anguilla
- ◯ Antarctica
- ◯ Antigua and Barbuda
- ◯ Argentina
- ◯ Armenia
- ◯ Aruba
- ◯ Australia
- ◯ Austria
- ◯ Azerbaijan
- ◯ Bahamas
- ◯ Bahrain
- ◯ Bangladesh
- ◯ Barbados
- ◯ Belarus
- ◯ Belgium
- ◯ Belize
- ◯ Benin
- ◯ Bermuda
- ◯ Bhutan
- ◯ Bolivia
- ◯ Bonaire, Sint Eustatius and Saba
- ◯ Bosnia and Herzegovina
- ◯ Botswana
- ◯ Bouvet Island
- ◯ Brazil
- ◯ British Indian Ocean Territory
- ◯ Brunei Darussalam
- ◯ Bulgaria
- ◯ Burkina Faso
- ◯ Burundi
- ◯ Cabo Verde
- ◯ Cambodia
- ◯ Cameroon
- ◯ Canada
- ◯ Cayman Islands
- ◯ Central African Republic
- ◯ Chad
- ◯ Chile
- ◯ China
- ◯ Christmas Island
- ◯ Cocos (Keeling) Islands
- ◯ Colombia
- ◯ Comoros
- ◯ Congo-Brazzaville
- ◯ Congo (DRC)
- ◯ Cook Islands
- ◯ Costa Rica
- ◯ Côte d'Ivoire
- ◯ Croatia
- ◯ Cuba
- ◯ Curaçao
- ◯ Cyprus
- ◯ Czechia
- ◯ Denmark
- ◯ Djibouti
- ◯ Dominica
- ◯ Dominican Republic
- ◯ Ecuador
- ◯ Egypt
- ◯ El Salvador
- ◯ Equatorial Guinea

◯ Eritrea
◯ Estonia
◯ Ethiopia
◯ Falkland Islands
◯ Faroe Islands
◯ Fiji
◯ Finland
◯ France
◯ French Guiana
◯ French Polynesia
◯ French Southern Territories
◯ Gabon
◯ Gambia
◯ Georgia
◯ Germany
◯ Ghana
◯ Gibraltar
◯ Greece
◯ Greenland
◯ Grenada
◯ Guadeloupe
◯ Guam
◯ Guatemala
◯ Guernsey
◯ Guinea
◯ Guinea-Bissau
◯ Guyana
◯ Haiti
◯ Heard Island and McDonald Islands
◯ Holy See
◯ Honduras
◯ Hong Kong
◯ Hungary
◯ Iceland
◯ India
◯ Indonesia
◯ Iran
◯ Iraq
◯ Ireland
◯ Isle of Man
◯ Israel
◯ Italy
◯ Jamaica
◯ Japan
◯ Jersey
◯ Jordan
◯ Kazakhstan
◯ Kenya
◯ Kiribati
◯ Kuwait
◯ Kyrgyzstan
◯ Laos
◯ Latvia
◯ Lebanon
◯ Lesotho
◯ Liberia
◯ Libya
◯ Liechtenstein
◯ Lithuania
◯ Luxembourg
◯ Macao
◯ Macedonia
◯ Madagascar
◯ Malawi
◯ Malaysia
◯ Maldives
◯ Mali
◯ Malta
◯ Marshall Islands
◯ Martinique
◯ Mauritania

◯ Mauritius
◯ Mayotte
◯ Mexico
◯ Micronesia
◯ Moldova
◯ Monaco
◯ Mongolia
◯ Montenegro
◯ Montserrat
◯ Morocco
◯ Mozambique
◯ Myanmar
◯ Namibia
◯ Nauru
◯ Nepal
◯ Netherlands
◯ New Caledonia
◯ New Zealand
◯ Nicaragua
◯ Niger
◯ Nigeria
◯ Niue
◯ Norfolk Island
◯ North Korea
◯ Northern Mariana Islands
◯ Norway
◯ Oman
◯ Pakistan
◯ Palau
◯ Palestine
◯ Panama
◯ Papua New Guinea
◯ Paraguay
◯ Peru
◯ Philippines
◯ Pitcairn
◯ Poland
◯ Portugal
◯ Puerto Rico
◯ Qatar
◯ Réunion
◯ Romania
◯ Russian Federation
◯ Rwanda
◯ Saint Barthélemy
◯ Saint Helena, Ascension and Tristan da Cunha
◯ Saint Kitts and Nevis
◯ Saint Lucia
◯ Saint Martin (French part)
◯ Saint Pierre and Miquelon
◯ Saint Vincent and the Grenadines
◯ Samoa
◯ San Marino
◯ Sao Tome and Principe
◯ Saudi Arabia
◯ Senegal
◯ Serbia
◯ Seychelles
◯ Sierra Leone
◯ Singapore
◯ Sint Maarten (Dutch part)
◯ Slovakia
◯ Slovenia
◯ Solomon Islands
◯ Somalia
◯ South Africa
◯ South Georgia and the South Sandwich Islands
◯ South Korea
◯ South Sudan
◯ Spain
◯ Sri Lanka

◯ Sudan
◯ Suriname
◯ Svalbard and Jan Mayen
◯ Swaziland
◯ Sweden
◯ Switzerland
◯ Syria
◯ Taiwan
◯ Tajikistan
◯ Tanzania
◯ Thailand
◯ Timor-Leste
◯ Togo
◯ Tokelau
◯ Tonga
◯ Trinidad and Tobago
◯ Tunisia
◯ Turkey
◯ Turkmenistan
◯ Turks and Caicos Islands
◯ Tuvalu
◯ Uganda
◯ Ukraine
◯ United Arab Emirates
◯ United Kingdom
◯ United States of America
◯ United States Minor Outlying Islands
◯ Uruguay
◯ Uzbekistan
◯ Vanuatu
◯ Venezuela
◯ Vietnam
◯ Virgin Islands (British)
◯ Virgin Islands (U.S.)
◯ Wallis and Futuna
◯ Yemen
◯ Zambia
◯ Zimbabwe
◯ Other (specify)
(Start typing to search)

Specify country of birth

____

Country of main residence

- ◯ Unknown
- ◯ Afghanistan
- ◯ Åland Islands
- ◯ Albania
- ◯ Algeria
- ◯ American Samoa
- ◯ Andorra
- ◯ Angola
- ◯ Anguilla
- ◯ Antarctica
- ◯ Antigua and Barbuda
- ◯ Argentina
- ◯ Armenia
- ◯ Aruba
- ◯ Australia
- ◯ Austria
- ◯ Azerbaijan
- ◯ Bahamas
- ◯ Bahrain
- ◯ Bangladesh
- ◯ Barbados
- ◯ Belarus
- ◯ Belgium
- ◯ Belize
- ◯ Benin
- ◯ Bermuda
- ◯ Bhutan
- ◯ Bolivia
- ◯ Bonaire, Sint Eustatius and Saba
- ◯ Bosnia and Herzegovina
- ◯ Botswana
- ◯ Bouvet Island
- ◯ Brazil
- ◯ British Indian Ocean Territory
- ◯ Brunei Darussalam
- ◯ Bulgaria
- ◯ Burkina Faso
- ◯ Burundi
- ◯ Cabo Verde
- ◯ Cambodia
- ◯ Cameroon
- ◯ Canada
- ◯ Cayman Islands
- ◯ Central African Republic
- ◯ Chad
- ◯ Chile
- ◯ China
- ◯ Christmas Island
- ◯ Cocos (Keeling) Islands
- ◯ Colombia
- ◯ Comoros
- ◯ Congo-Brazzaville
- ◯ Congo (DRC)
- ◯ Cook Islands
- ◯ Costa Rica
- ◯ Côte d'Ivoire
- ◯ Croatia
- ◯ Cuba
- ◯ Curaçao
- ◯ Cyprus
- ◯ Czechia
- ◯ Denmark
- ◯ Djibouti
- ◯ Dominica
- ◯ Dominican Republic
- ◯ Ecuador
- ◯ Egypt
- ◯ El Salvador
- ◯ Equatorial Guinea

◯ Eritrea
◯ Estonia
◯ Ethiopia
◯ Falkland Islands
◯ Faroe Islands
◯ Fiji
◯ Finland
◯ France
◯ French Guiana
◯ French Polynesia
◯ French Southern Territories
◯ Gabon
◯ Gambia
◯ Georgia
◯ Germany
◯ Ghana
◯ Gibraltar
◯ Greece
◯ Greenland
◯ Grenada
◯ Guadeloupe
◯ Guam
◯ Guatemala
◯ Guernsey
◯ Guinea
◯ Guinea-Bissau
◯ Guyana
◯ Haiti
◯ Heard Island and McDonald Islands
◯ Holy See
◯ Honduras
◯ Hong Kong
◯ Hungary
◯ Iceland
◯ India
◯ Indonesia
◯ Iran
◯ Iraq
◯ Ireland
◯ Isle of Man
◯ Israel
◯ Italy
◯ Jamaica
◯ Japan
◯ Jersey
◯ Jordan
◯ Kazakhstan
◯ Kenya
◯ Kiribati
◯ Kuwait
◯ Kyrgyzstan
◯ Laos
◯ Latvia
◯ Lebanon
◯ Lesotho
◯ Liberia
◯ Libya
◯ Liechtenstein
◯ Lithuania
◯ Luxembourg
◯ Macao
◯ Macedonia
◯ Madagascar
◯ Malawi
◯ Malaysia
◯ Maldives
◯ Mali
◯ Malta
◯ Marshall Islands
◯ Martinique
◯ Mauritania

◯ Mauritius
◯ Mayotte
◯ Mexico
◯ Micronesia
◯ Moldova
◯ Monaco
◯ Mongolia
◯ Montenegro
◯ Montserrat
◯ Morocco
◯ Mozambique
◯ Myanmar
◯ Namibia
◯ Nauru
◯ Nepal
◯ Netherlands
◯ New Caledonia
◯ New Zealand
◯ Nicaragua
◯ Niger
◯ Nigeria
◯ Niue
◯ Norfolk Island
◯ North Korea
◯ Northern Mariana Islands
◯ Norway
◯ Oman
◯ Pakistan
◯ Palau
◯ Palestine
◯ Panama
◯ Papua New Guinea
◯ Paraguay
◯ Peru
◯ Philippines
◯ Pitcairn
◯ Poland
◯ Portugal
◯ Puerto Rico
◯ Qatar
◯ Réunion
◯ Romania
◯ Russian Federation
◯ Rwanda
◯ Saint Barthélemy
◯ Saint Helena, Ascension and Tristan da Cunha
◯ Saint Kitts and Nevis
◯ Saint Lucia
◯ Saint Martin (French part)
◯ Saint Pierre and Miquelon
◯ Saint Vincent and the Grenadines
◯ Samoa
◯ San Marino
◯ Sao Tome and Principe
◯ Saudi Arabia
◯ Senegal
◯ Serbia
◯ Seychelles
◯ Sierra Leone
◯ Singapore
◯ Sint Maarten (Dutch part)
◯ Slovakia
◯ Slovenia
◯ Solomon Islands
◯ Somalia
◯ South Africa
◯ South Georgia and the South Sandwich Islands
◯ South Korea
◯ South Sudan
◯ Spain
◯ Sri Lanka

◯ Sudan
◯ Suriname
◯ Svalbard and Jan Mayen
◯ Swaziland
◯ Sweden
◯ Switzerland
◯ Syria
◯ Taiwan
◯ Tajikistan
◯ Tanzania
◯ Thailand
◯ Timor-Leste
◯ Togo
◯ Tokelau
◯ Tonga
◯ Trinidad and Tobago
◯ Tunisia
◯ Turkey
◯ Turkmenistan
◯ Turks and Caicos Islands
◯ Tuvalu
◯ Uganda
◯ Ukraine
◯ United Arab Emirates
◯ United Kingdom
◯ United States of America
◯ United States Minor Outlying Islands
◯ Uruguay
◯ Uzbekistan
◯ Vanuatu
◯ Venezuela
◯ Vietnam
◯ Virgin Islands (British)
◯ Virgin Islands (U.S.)
◯ Wallis and Futuna
◯ Yemen
◯ Zambia
◯ Zimbabwe
◯ Other (specify)
(Start typing to search)

Specify country of residence

____

Source file code

____
(Please specify the internal patient file code at your institution. It won't be included in data exportation for analysis.)

Travel information

Exposure context

Patient exposure context

◯ Short-term traveler (< 6 months)
◯ Long-term traveler / expatriate (> 6 months)
◯ Migrant

Which was the reason for traveling?

◯ Tourism / Leisure
◯ Visiting friends or relatives (VFR)
◯ Business or professional trip
◯ Humanitarian mission / Volunteer work
◯ Religious pilgrimage
◯ Other

Please specify other reason for traveling

____

Which was the reason for traveling?

◯ Humanitarian mission / volunteer work
◯ Professional / academic assignment
◯ Visiting friends or relatives (VFR)
◯ Long-term tourism / travel
◯ Retirement migration
◯ Other (specify)

Please specify other reason for traveling

____

Date of departure

____

Date of return

____

Date of arrival

____

Country of (most likely) exposure

- ◯ Unknown
- ◯ Afghanistan
- ◯ Åland Islands
- ◯ Albania
- ◯ Algeria
- ◯ American Samoa
- ◯ Andorra
- ◯ Angola
- ◯ Anguilla
- ◯ Antarctica
- ◯ Antigua and Barbuda
- ◯ Argentina
- ◯ Armenia
- ◯ Aruba
- ◯ Australia
- ◯ Austria
- ◯ Azerbaijan
- ◯ Bahamas
- ◯ Bahrain
- ◯ Bangladesh
- ◯ Barbados
- ◯ Belarus
- ◯ Belgium
- ◯ Belize
- ◯ Benin
- ◯ Bermuda
- ◯ Bhutan
- ◯ Bolivia
- ◯ Bonaire, Sint Eustatius and Saba
- ◯ Bosnia and Herzegovina
- ◯ Botswana
- ◯ Bouvet Island
- ◯ Brazil
- ◯ British Indian Ocean Territory
- ◯ Brunei Darussalam
- ◯ Bulgaria
- ◯ Burkina Faso
- ◯ Burundi
- ◯ Cabo Verde
- ◯ Cambodia
- ◯ Cameroon
- ◯ Canada
- ◯ Cayman Islands
- ◯ Central African Republic
- ◯ Chad
- ◯ Chile
- ◯ China
- ◯ Christmas Island
- ◯ Cocos (Keeling) Islands
- ◯ Colombia
- ◯ Comoros
- ◯ Congo-Brazzaville
- ◯ Congo (DRC)
- ◯ Cook Islands
- ◯ Costa Rica
- ◯ Côte d'Ivoire
- ◯ Croatia
- ◯ Cuba
- ◯ Curaçao
- ◯ Cyprus
- ◯ Czechia
- ◯ Denmark
- ◯ Djibouti
- ◯ Dominica
- ◯ Dominican Republic
- ◯ Ecuador
- ◯ Egypt
- ◯ El Salvador
- ◯ Equatorial Guinea

◯ Eritrea
◯ Estonia
◯ Ethiopia
◯ Falkland Islands
◯ Faroe Islands
◯ Fiji
◯ Finland
◯ France
◯ French Guiana
◯ French Polynesia
◯ French Southern Territories
◯ Gabon
◯ Gambia
◯ Georgia
◯ Germany
◯ Ghana
◯ Gibraltar
◯ Greece
◯ Greenland
◯ Grenada
◯ Guadeloupe
◯ Guam
◯ Guatemala
◯ Guernsey
◯ Guinea
◯ Guinea-Bissau
◯ Guyana
◯ Haiti
◯ Heard Island and McDonald Islands
◯ Holy See
◯ Honduras
◯ Hong Kong
◯ Hungary
◯ Iceland
◯ India
◯ Indonesia
◯ Iran
◯ Iraq
◯ Ireland
◯ Isle of Man
◯ Israel
◯ Italy
◯ Jamaica
◯ Japan
◯ Jersey
◯ Jordan
◯ Kazakhstan
◯ Kenya
◯ Kiribati
◯ Kuwait
◯ Kyrgyzstan
◯ Laos
◯ Latvia
◯ Lebanon
◯ Lesotho
◯ Liberia
◯ Libya
◯ Liechtenstein
◯ Lithuania
◯ Luxembourg
◯ Macao
◯ Macedonia
◯ Madagascar
◯ Malawi
◯ Malaysia
◯ Maldives
◯ Mali
◯ Malta
◯ Marshall Islands
◯ Martinique
◯ Mauritania

◯ Mauritius
◯ Mayotte
◯ Mexico
◯ Micronesia
◯ Moldova
◯ Monaco
◯ Mongolia
◯ Montenegro
◯ Montserrat
◯ Morocco
◯ Mozambique
◯ Myanmar
◯ Namibia
◯ Nauru
◯ Nepal
◯ Netherlands
◯ New Caledonia
◯ New Zealand
◯ Nicaragua
◯ Niger
◯ Nigeria
◯ Niue
◯ Norfolk Island
◯ North Korea
◯ Northern Mariana Islands
◯ Norway
◯ Oman
◯ Pakistan
◯ Palau
◯ Palestine
◯ Panama
◯ Papua New Guinea
◯ Paraguay
◯ Peru
◯ Philippines
◯ Pitcairn
◯ Poland
◯ Portugal
◯ Puerto Rico
◯ Qatar
◯ Réunion
◯ Romania
◯ Russian Federation
◯ Rwanda
◯ Saint Barthélemy
◯ Saint Helena, Ascension and Tristan da Cunha
◯ Saint Kitts and Nevis
◯ Saint Lucia
◯ Saint Martin (French part)
◯ Saint Pierre and Miquelon
◯ Saint Vincent and the Grenadines
◯ Samoa
◯ San Marino
◯ Sao Tome and Principe
◯ Saudi Arabia
◯ Senegal
◯ Serbia
◯ Seychelles
◯ Sierra Leone
◯ Singapore
◯ Sint Maarten (Dutch part)
◯ Slovakia
◯ Slovenia
◯ Solomon Islands
◯ Somalia
◯ South Africa
◯ South Georgia and the South Sandwich Islands
◯ South Korea
◯ South Sudan
◯ Spain
◯ Sri Lanka

○ Sudan
○ Suriname
○ Svalbard and Jan Mayen
○ Swaziland
○ Sweden
○ Switzerland
○ Syria
○ Taiwan
○ Tajikistan
○ Tanzania
○ Thailand
○ Timor-Leste
○ Togo
○ Tokelau
○ Tonga
○ Trinidad and Tobago
○ Tunisia
○ Turkey
○ Turkmenistan
○ Turks and Caicos Islands
○ Tuvalu
○ Uganda
○ Ukraine
○ United Arab Emirates
○ United Kingdom
○ United States of America
○ United States Minor Outlying Islands
○ Uruguay
○ Uzbekistan
○ Vanuatu
○ Venezuela
○ Vietnam
○ Virgin Islands (British)
○ Virgin Islands (U.S.)
○ Wallis and Futuna
○ Yemen
○ Zambia
○ Zimbabwe
○ Other (specify)
(Start typing to search)

Specify country of likely exposure

____

Date of first exposure to surface water

____
(If exact date unknown, best estimate)

Place of exposure

○ Lake
○ River
○ Other
○ Unknown

Please specify name

____

Other possible country of exposure

◯ Not applicable
◯ Unknown
◯ Afghanistan
◯ Åland Islands
◯ Albania
◯ Algeria
◯ American Samoa
◯ Andorra
◯ Angola
◯ Anguilla
◯ Antarctica
◯ Antigua and Barbuda
◯ Argentina
◯ Armenia
◯ Aruba
◯ Australia
◯ Austria
◯ Azerbaijan
◯ Bahamas
◯ Bahrain
◯ Bangladesh
◯ Barbados
◯ Belarus
◯ Belgium
◯ Belize
◯ Benin
◯ Bermuda
◯ Bhutan
◯ Bolivia
◯ Bonaire, Sint Eustatius and Saba
◯ Bosnia and Herzegovina
◯ Botswana
◯ Bouvet Island
◯ Brazil
◯ British Indian Ocean Territory
◯ Brunei Darussalam
◯ Bulgaria
◯ Burkina Faso
◯ Burundi
◯ Cabo Verde
◯ Cambodia
◯ Cameroon
◯ Canada
◯ Cayman Islands
◯ Central African Republic
◯ Chad
◯ Chile
◯ China
◯ Christmas Island
◯ Cocos (Keeling) Islands
◯ Colombia
◯ Comoros
◯ Congo-Brazzaville
◯ Congo (DRC)
◯ Cook Islands
◯ Costa Rica
◯ Côte d'Ivoire
◯ Croatia
◯ Cuba
◯ Curaçao
◯ Cyprus
◯ Czechia
◯ Denmark
◯ Djibouti
◯ Dominica
◯ Dominican Republic
◯ Ecuador
◯ Egypt
◯ El Salvador

◯ Equatorial Guinea
◯ Eritrea
◯ Estonia
◯ Ethiopia
◯ Falkland Islands
◯ Faroe Islands
◯ Fiji
◯ Finland
◯ France
◯ French Guiana
◯ French Polynesia
◯ French Southern Territories
◯ Gabon
◯ Gambia
◯ Georgia
◯ Germany
◯ Ghana
◯ Gibraltar
◯ Greece
◯ Greenland
◯ Grenada
◯ Guadeloupe
◯ Guam
◯ Guatemala
◯ Guernsey
◯ Guinea
◯ Guinea-Bissau
◯ Guyana
◯ Haiti
◯ Heard Island and McDonald Islands
◯ Holy See
◯ Honduras
◯ Hong Kong
◯ Hungary
◯ Iceland
◯ India
◯ Indonesia
◯ Iran
◯ Iraq
◯ Ireland
◯ Isle of Man
◯ Israel
◯ Italy
◯ Jamaica
◯ Japan
◯ Jersey
◯ Jordan
◯ Kazakhstan
◯ Kenya
◯ Kiribati
◯ Kuwait
◯ Kyrgyzstan
◯ Laos
◯ Latvia
◯ Lebanon
◯ Lesotho
◯ Liberia
◯ Libya
◯ Liechtenstein
◯ Lithuania
◯ Luxembourg
◯ Macao
◯ Macedonia
◯ Madagascar
◯ Malawi
◯ Malaysia
◯ Maldives
◯ Mali
◯ Malta
◯ Marshall Islands
◯ Martinique

◯ Mauritania
◯ Mauritius
◯ Mayotte
◯ Mexico
◯ Micronesia
◯ Moldova
◯ Monaco
◯ Mongolia
◯ Montenegro
◯ Montserrat
◯ Morocco
◯ Mozambique
◯ Myanmar
◯ Namibia
◯ Nauru
◯ Nepal
◯ Netherlands
◯ New Caledonia
◯ New Zealand
◯ Nicaragua
◯ Niger
◯ Nigeria
◯ Niue
◯ Norfolk Island
◯ North Korea
◯ Northern Mariana Islands
◯ Norway
◯ Oman
◯ Pakistan
◯ Palau
◯ Palestine
◯ Panama
◯ Papua New Guinea
◯ Paraguay
◯ Peru
◯ Philippines
◯ Pitcairn
◯ Poland
◯ Portugal
◯ Puerto Rico
◯ Qatar
◯ Réunion
◯ Romania
◯ Russian Federation
◯ Rwanda
◯ Saint Barthélemy
◯ Saint Helena, Ascension and Tristan da Cunha
◯ Saint Kitts and Nevis
◯ Saint Lucia
◯ Saint Martin (French part)
◯ Saint Pierre and Miquelon
◯ Saint Vincent and the Grenadines
◯ Samoa
◯ San Marino
◯ Sao Tome and Principe
◯ Saudi Arabia
◯ Senegal
◯ Serbia
◯ Seychelles
◯ Sierra Leone
◯ Singapore
◯ Sint Maarten (Dutch part)
◯ Slovakia
◯ Slovenia
◯ Solomon Islands
◯ Somalia
◯ South Africa
◯ South Georgia and the South Sandwich Islands
◯ South Korea
◯ South Sudan
◯ Spain

◯ Sri Lanka
◯ Sudan
◯ Suriname
◯ Svalbard and Jan Mayen
◯ Swaziland
◯ Sweden
◯ Switzerland
◯ Syria
◯ Taiwan
◯ Tajikistan
◯ Tanzania
◯ Thailand
◯ Timor-Leste
◯ Togo
◯ Tokelau
◯ Tonga
◯ Trinidad and Tobago
◯ Tunisia
◯ Turkey
◯ Turkmenistan
◯ Turks and Caicos Islands
◯ Tuvalu
◯ Uganda
◯ Ukraine
◯ United Arab Emirates
◯ United Kingdom
◯ United States of America
◯ United States Minor Outlying Islands
◯ Uruguay
◯ Uzbekistan
◯ Vanuatu
◯ Venezuela
◯ Vietnam
◯ Virgin Islands (British)
◯ Virgin Islands (U.S.)
◯ Wallis and Futuna
◯ Yemen
◯ Zambia
◯ Zimbabwe
◯ Other
(Start typing to search)

Specify other country of likely exposure

____

2nd other possible country of exposure

◯ Not applicable
◯ Unknown
◯ Afghanistan
◯ Åland Islands
◯ Albania
◯ Algeria
◯ American Samoa
◯ Andorra
◯ Angola
◯ Anguilla
◯ Antarctica
◯ Antigua and Barbuda
◯ Argentina
◯ Armenia
◯ Aruba
◯ Australia
◯ Austria
◯ Azerbaijan
◯ Bahamas
◯ Bahrain
◯ Bangladesh
◯ Barbados
◯ Belarus
◯ Belgium
◯ Belize
◯ Benin
◯ Bermuda
◯ Bhutan
◯ Bolivia
◯ Bonaire, Sint Eustatius and Saba
◯ Bosnia and Herzegovina
◯ Botswana
◯ Bouvet Island
◯ Brazil
◯ British Indian Ocean Territory
◯ Brunei Darussalam
◯ Bulgaria
◯ Burkina Faso
◯ Burundi
◯ Cabo Verde
◯ Cambodia
◯ Cameroon
◯ Canada
◯ Cayman Islands
◯ Central African Republic
◯ Chad
◯ Chile
◯ China
◯ Christmas Island
◯ Cocos (Keeling) Islands
◯ Colombia
◯ Comoros
◯ Congo-Brazzaville
◯ Congo (DRC)
◯ Cook Islands
◯ Costa Rica
◯ Côte d'Ivoire
◯ Croatia
◯ Cuba
◯ Curaçao
◯ Cyprus
◯ Czechia
◯ Denmark
◯ Djibouti
◯ Dominica
◯ Dominican Republic
◯ Ecuador
◯ Egypt
◯ El Salvador

◯ Equatorial Guinea
◯ Eritrea
◯ Estonia
◯ Ethiopia
◯ Falkland Islands
◯ Faroe Islands
◯ Fiji
◯ Finland
◯ France
◯ French Guiana
◯ French Polynesia
◯ French Southern Territories
◯ Gabon
◯ Gambia
◯ Georgia
◯ Germany
◯ Ghana
◯ Gibraltar
◯ Greece
◯ Greenland
◯ Grenada
◯ Guadeloupe
◯ Guam
◯ Guatemala
◯ Guernsey
◯ Guinea
◯ Guinea-Bissau
◯ Guyana
◯ Haiti
◯ Heard Island and McDonald Islands
◯ Holy See
◯ Honduras
◯ Hong Kong
◯ Hungary
◯ Iceland
◯ India
◯ Indonesia
◯ Iran
◯ Iraq
◯ Ireland
◯ Isle of Man
◯ Israel
◯ Italy
◯ Jamaica
◯ Japan
◯ Jersey
◯ Jordan
◯ Kazakhstan
◯ Kenya
◯ Kiribati
◯ Kuwait
◯ Kyrgyzstan
◯ Laos
◯ Latvia
◯ Lebanon
◯ Lesotho
◯ Liberia
◯ Libya
◯ Liechtenstein
◯ Lithuania
◯ Luxembourg
◯ Macao
◯ Macedonia
◯ Madagascar
◯ Malawi
◯ Malaysia
◯ Maldives
◯ Mali
◯ Malta
◯ Marshall Islands
◯ Martinique

◯ Mauritania
◯ Mauritius
◯ Mayotte
◯ Mexico
◯ Micronesia
◯ Moldova
◯ Monaco
◯ Mongolia
◯ Montenegro
◯ Montserrat
◯ Morocco
◯ Mozambique
◯ Myanmar
◯ Namibia
◯ Nauru
◯ Nepal
◯ Netherlands
◯ New Caledonia
◯ New Zealand
◯ Nicaragua
◯ Niger
◯ Nigeria
◯ Niue
◯ Norfolk Island
◯ North Korea
◯ Northern Mariana Islands
◯ Norway
◯ Oman
◯ Pakistan
◯ Palau
◯ Palestine
◯ Panama
◯ Papua New Guinea
◯ Paraguay
◯ Peru
◯ Philippines
◯ Pitcairn
◯ Poland
◯ Portugal
◯ Puerto Rico
◯ Qatar
◯ Réunion
◯ Romania
◯ Russian Federation
◯ Rwanda
◯ Saint Barthélemy
◯ Saint Helena, Ascension and Tristan da Cunha
◯ Saint Kitts and Nevis
◯ Saint Lucia
◯ Saint Martin (French part)
◯ Saint Pierre and Miquelon
◯ Saint Vincent and the Grenadines
◯ Samoa
◯ San Marino
◯ Sao Tome and Principe
◯ Saudi Arabia
◯ Senegal
◯ Serbia
◯ Seychelles
◯ Sierra Leone
◯ Singapore
◯ Sint Maarten (Dutch part)
◯ Slovakia
◯ Slovenia
◯ Solomon Islands
◯ Somalia
◯ South Africa
◯ South Georgia and the South Sandwich Islands
◯ South Korea
◯ South Sudan
◯ Spain

	◯ Sri Lanka<br>◯ Sudan<br>◯ Suriname<br>◯ Svalbard and Jan Mayen<br>◯ Swaziland<br>◯ Sweden<br>◯ Switzerland<br>◯ Syria<br>◯ Taiwan<br>◯ Tajikistan<br>◯ Tanzania<br>◯ Thailand<br>◯ Timor-Leste<br>◯ Togo<br>◯ Tokelau<br>◯ Tonga<br>◯ Trinidad and Tobago<br>◯ Tunisia<br>◯ Turkey<br>◯ Turkmenistan<br>◯ Turks and Caicos Islands<br>◯ Tuvalu<br>◯ Uganda<br>◯ Ukraine<br>◯ United Arab Emirates<br>◯ United Kingdom<br>◯ United States of America<br>◯ United States Minor Outlying Islands<br>◯ Uruguay<br>◯ Uzbekistan<br>◯ Vanuatu<br>◯ Venezuela<br>◯ Vietnam<br>◯ Virgin Islands (British)<br>◯ Virgin Islands (U.S.)<br>◯ Wallis and Futuna<br>◯ Yemen<br>◯ Zambia<br>◯ Zimbabwe<br>◯ Other<br>(Start typing to search)
Specify other country of likely exposure	______________________
Is this case part of a cluster?	◯ Yes<br>◯ No<br>◯ Unknown

Pre-travel consultation and malaria prophylaxis

Did the patient seek a pre-travel consultation before the trip?	◯ Yes<br>◯ No<br>◯ Unknown
Did the patient take malaria chemoprophylaxis during the trip?	◯ Yes<br>◯ No<br>◯ Unknown

Which malaria prophylaxis was taken?	◯ Atovaquone-proguanil<br>◯ Mefloquine<br>◯ Doxycycline<br>◯ Chloroquine<br>◯ Other (specify)<br>◯ Unknown
Specify other malaria prophylaxis	__________________________________
Additional comments in this section (travel-related information)	__________________________________<br>(Please include more information if the exposure is unclear or there is multidestination travel)

1st visit: Clinical features

Date of first visit

____

Did the patient experience a skin rash compatible with swimmer's dermatitis (e.g., itchy, maculopapular or urticarial rash) shortly after water exposure?

- ◯ Yes
- ◯ No
- ◯ Unknown

Did the patient report any symptoms prior to the consultation?

- ◯ Yes
- ◯ No
- ◯ Not assessed

(Symptoms related to initial swimmer's dermatitis should NOT be considered in this field.)

Which symptoms were present before the initial visit?

- ☐ Fever
- ☐ Chills
- ☐ Fatigue
- ☐ Myalgias/arthralgias
- ☐ Headache
- ☐ Abdominal pain
- ☐ Nausea/vomiting
- ☐ Diarrhea
- ☐ Dyspnea
- ☐ Cough
- ☐ Urticaria
- ☐ Angioedema
- ☐ Other

Which other?

____

Does the patient have any symptoms at the time of the visit?

- ◯ Yes
- ◯ No
- ◯ Not assessed

Which symptoms are present at the current visit?

- ☐ Fever
- ☐ Chills
- ☐ Fatigue
- ☐ Myalgias/arthralgias
- ☐ Headache
- ☐ Abdominal pain
- ☐ Nausea/vomiting
- ☐ Diarrhea
- ☐ Dyspnea
- ☐ Cough
- ☐ Urticaria
- ☐ Angioedema
- ☐ Other

Which other?

____

Date of onset of main symptoms

____

(This refers to the date when any symptom first began, whether it is now resolved (previous) or still present (current).)

Weight (kg)	__________________________________
Any abnormal clinical findings in physical examination?	◯ Yes<br>◯ No<br>◯ Unknown
Please specify abnormal clinical findings	__________________________________
Any underlying conditions?	◯ Yes<br>◯ No<br>◯ Unknown
Which one(s)?	☐ Hypertension<br>☐ Dyslipidemia<br>☐ Diabetes mellitus<br>☐ Cardiovascular disease (ischemic heart disease, myocardial infarction, heart failure, stroke...)<br>☐ Respiratory disease (asthma, COPD, interstitial lung disease...)<br>☐ Chronic kidney disease<br>☐ Chronic liver disease<br>☐ Immunocompromising condition<br>☐ Pregnancy at diagnosis<br>☐ Other
Please specify other underlying condition(s)	__________________________________
Type of immunocompromising condition	☐ HIV infection<br>☐ Solid organ transplantation<br>☐ Hematological malignancy / Stem cell transplantation<br>☐ Conventional immunosuppressants (e.g., corticosteroids, methotrexate)<br>☐ Biological immunosuppressants (e.g., anti-TNF, IL-6 inhibitors)<br>☐ Other (specify)<br>☐ Unknown
HIV infection	◯ CD4 count < 200/mm3<br>◯ CD4 count >200/mm3/AIDS events<br>◯ Unknown
Which organ?	☐ Kidney<br>☐ Liver<br>☐ Heart<br>☐ Lung<br>☐ Pancreas
Hematological malignancy / Stem cell transplantation	☐ Acute or chronic leukemia<br>☐ Lymphomas (Hodgkin and non-Hodgkin)<br>☐ Multiple myeloma<br>☐ Myelodysplastic syndromes<br>☐ Autologous HSCT<br>☐ Allogeneic HSCT<br>☐ Other

Conventional immunosuppressants	☐ Corticosteroids<br>☐ Antimetabolites: Azathioprine, methotrexate, leflunomide<br>☐ Calcineurin inhibitors: Cyclosporine, tacrolimus<br>☐ Alkylating agents: Cyclophosphamide, chlorambucil<br>☐ mTOR inhibitors: Sirolimus, everolimus<br>☐ Other
Biological immunosuppressants	☐ TNF-α inhibitors: Infliximab, adalimumab, etanercept...<br>☐ IL-6 inhibitors: Tocilizumab, sarilumab...<br>☐ B-cell depleting agents: Rituximab, obinutuzumab...<br>☐ IL-1 blockers: Anakinra, canakinumab...<br>☐ Integrin inhibitors: Natalizumab, vedolizumab<br>☐ JAK inhibitors: Tofacitinib, baricitinib...<br>☐ Other
Please specify other immunocompromising condition/immunosuppressant	__________________________________

1st visit: Basic lab results

□□ Laboratory Results - Important Instructions

Please provide laboratory results that are closest in time to the clinical visit for which this form is being completed.

□□ Which results to enter:

Select values from the laboratory test(s) performed on the same day as the visit whenever possible.
If same-day results are not available, choose the nearest available lab test performed before or after the visit (ideally within ±7 days).

□□ Why this matters:

Using results closest to the clinical assessment ensures accurate correlation between clinical status and laboratory findings.

□ If no lab tests were performed around the time of the visit, leave the fields blank and add a note if necessary.

Was a blood analysis performed in the first visit?	◯ Yes<br>◯ No<br>◯ Unknown
Date of lab test	______________________<br>(Enter the date of the lab test performed on the same day as the clinical visit, or the nearest available test within ±7 days.)

Main lab results

Hemoglobin value	______________________
Hemoglobin values unit	◯ g/dL<br>◯ Other
Hemoglobin unit - specify other	______________________
WBC count	______________________
WBC count unit	◯ x10^9/L<br>◯ Other
WBC count unit - specify other	______________________
Eosinophil count	______________________
Eosinophil count unit	◯ cells/µL<br>◯ Other
Eosinophil count unit - specify other	______________________

Platelet count	________________________
Platelet count unit	◯ ×10^9/L<br>◯ Other
Platelet count unit - specify other	________________________
C-reactive protein value	________________________
C-reactive protein unit	◯ mg/L<br>◯ mg/dL<br>◯ Other
C-reactive protein unit - specify other	________________________
IgE value	________________________
IgE unit	◯ IU/mL<br>◯ kU/L<br>◯ Other
IgE unit - specify other	________________________

Other lab results

Any other baseline lab abnormalities found?	◯ Yes<br>◯ No
Which one(s)?	☐ Elevated ALT<br>☐ Elevated AST<br>☐ Elevated GGT<br>☐ Elevated Alkaline Phosphatase<br>☐ Elevated LDH<br>☐ Elevated Creatinine<br>☐ Elevated Urea<br>☐ Other (specify)
Which other lab abnormalities?	________________________
Were any of the following tests performed?	☐ Urinalysis<br>☐ Fecal occult blood test (FOBT)
Were any of the following abnormalities present?	☐ Proteinuria<br>☐ Hematuria<br>☐ Leukocyturia<br>☐ Other

Proteinuria - Type of assessment	☐ Dipstick (semi-quantitative)<br>☐ Quantitative (lab-based)<br>☐ Unknown
Proteinuria (dipstick result)	◯ Negative<br>◯ Trace<br>◯ 1+<br>◯ 2+<br>◯ 3+<br>◯ 4+
Proteinuria (quantitative result)	__________________________________
Proteinuria unit	◯ mg/dL<br>◯ mg/day<br>◯ g/day<br>◯ Protein-to-creatinine ratio (mg/g)<br>◯ Other
Specify other proteinuria unit	__________________________________
Hematuria (graded by dipstick or microscopy)	◯ Microscopic (< 10 RBCs/µL or trace)<br>◯ Mild (10-50 RBCs/µL or 1+)<br>◯ Moderate (50-250 RBCs/µL or 2+)<br>◯ Gross hematuria (>250 RBCs/µL or ≥3+)<br>◯ Unknown
Leukocyturia (dipstick or microscopy result)	◯ Trace / Few WBCs (< 10/µL)<br>◯ Moderate (10-50 WBCs/µL or 1-2+)<br>◯ Marked (>50 WBCs/µL or ≥3+)<br>◯ Unknown
Please describe other urinalysis abnormalities	__________________________________
Fecal occult blood test result?	◯ Positive<br>◯ Negative<br>◯ Indeterminate<br>◯ Invalid<br>◯ Unknown
Other comments	____

1st visit: Schisto-specific tests

⚠

Note on Diagnosis of Acute Schistosomiasis:

Diagnosis of acute schistosomiasis is very challenging, especially at first presentation for symptomatic patients. Microscopic examination to detect eggs in urine and/or stool is often negative because the sensitivity of this method, which is usually limited for schistosomiasis, is even lower as eggs may become detectable only 4 to 6 weeks after exposure/infection. Similarly, serological methods become usually positive a few weeks (up to 2-3 months) after infection, as they are often based on adult worm antigens.

For this reason, both methods must be either performed 8-12 weeks after exposure to screen asymptomatic exposed individuals or repeated at that time if they were negative at initial workup.

In general, if there is a strong clinical suspicion in symptomatic patients, therapy should be initiated before full confirmation.

Of note, novel methods such as PCR-based or Circulating Anionic Antigen (CAA)-based assays in serum become positive more rapidly, within the 6 first weeks after infection, but are usually not available in the clinical practice. Very few reference/research laboratories in Europe could perform these tests at this moment.

Schisto-specific tests

Please fill in the following table as detailed as possible.

⚠

Important Instructions for this section:

1

□ For each test, please ensure that only one option is selected. If multiple options are selected in a matrix row by mistake, use the "reset" button to deselect the incorrect choice.

2

□ For all results marked as Positive, Negative, or Indeterminate, please provide the numerical value of the test result whenever available (e.g., titer, egg count, Ct value, etc) in the box that appears to the right of each row.

Was any schisto-specific test ordered during the first visit?

◯ Yes
◯ No
◯ Unknown

Date of Schisto-specific test ______________________________

Positive Negative Indeterminate Not done
Antibody serology
IHA ______ ______ ______ ______ ______
ELISA ______ ______ ______ ______ ______
Other ______ ______ ______ ______ ______ ______
Egg detection
Eggs in urine ______ ______ ______ ______
Eggs in stool ______ ______ ______ ______
Antigenic tests
POC CCA ______ ______ ______ ______ ______
LFA CAA (lateral flow assay) ______ ______ ______ ______ ______
Other ______ ______ ______ ______ ______ ______
PCR
Serum Dra1 ______ ______ ______ ______

Serum Sm1-7 ______ ______ ______ ______

Urine 28S rRNA

______ ______ ______ ______

Stool 28S rRNA

______ ______ ______ ______

Other ______

______ ______ ______ ______

Further comments

____

(Please use this section to further explain your choice and what your institution uses)

1st visit Other relevant results

Imaging tests

Was any imaging test done in this visit?

- ◯ Yes
- ◯ No
- ◯ Unknown

Which imaging test(s) were performed?

- ☐ Chest X-ray
- ☐ Abdominal ultrasound
- ☐ Chest CT scan
- ☐ Abdominal CT scan
- ☐ Brain CT scan
- ☐ Brain MRI
- ☐ Spinal cord MRI
- ☐ Hepatic MRI
- ☐ Echocardiogram
- ☐ PET-CT scan
- ☐ Other

(Check all that apply)

Specify which other imaging test was performed

____

Date of chest X-ray

____

Chest X-ray - Main Findings

- ☐ Normal
- ☐ Pulmonary infiltrates
- ☐ Interstitial opacities
- ☐ Pleural effusion
- ☐ Hilar or mediastinal lymphadenopathy
- ☐ Cardiomegaly
- ☐ Other (specify)

Specify other abnormal findings on chest X-ray

____

Date of abdominal US

____

Abdominal Ultrasound - Main Findings

- ☐ Normal
- ☐ Hepatosplenomegaly
- ☐ Splenomegaly only
- ☐ Hyperechogenic liver
- ☐ Enlarged periportal lymph nodes
- ☐ Ascites
- ☐ Other (specify)

Specify other abnormal findings on abdominal US

____

Date of chest CT scan

____

Chest CT scan - Main Findings	☐ Normal<br>☐ Pulmonary nodules<br>☐ Ground-glass opacities<br>☐ Interstitial infiltrates<br>☐ Pleural effusion<br>☐ Enlarged mediastinal or hilar lymph nodes<br>☐ Other (specify)
Specify other abnormal findings on chest CT scan	__________________________________
Date of abdominal CT scan	__________________________________
Abdominal CT scan - Main Findings	☐ Normal<br>☐ Hepatosplenomegaly<br>☐ Splenomegaly only<br>☐ Enlarged abdominal or periportal lymph nodes<br>☐ Focal hepatic lesions (e.g., granulomas or nodules)<br>☐ Ascites<br>☐ Other (specify)
Specify other abnormal findings on abdominal CT scan	__________________________________
Date of brain CT scan	__________________________________
Please describe any abnormal imaging findings on brain CT	__________________________________
Date of brain MRI	__________________________________
Please describe any abnormal imaging findings on brain MRI	__________________________________
Date of spinal cord MRI	__________________________________
Please describe any abnormal imaging findings on spinal cord MRI	__________________________________
Date of hepatic MRI	__________________________________
Please describe any abnormal imaging findings on hepatic MRI	__________________________________
Date of echocardiogram	__________________________________
Please describe any abnormal imaging findings on echocardiogram	__________________________________
Date of PET-CT scan	__________________________________

Please describe any abnormal imaging findings on PET-CT scan ________________________

Date of other imaging test ________________________

Please describe any abnormal imaging findings on other imaging tests ________________________

Concomitant infections

Were other concomitant infections diagnosed?
- ◯ Yes
- ◯ No

Which one(s)?
- ☐ Malaria
- ☐ Dengue
- ☐ Strongyloidiasis
- ☐ Leptospirosis
- ☐ Other

Other (specify) ________________________

Comments on other relevant investigations or diagnosis ________________________

1st visit: Case definition

How would you define the case at first visit?

Choose one Case classification Epidemiological criteria Microbiological criteria Clinical criteria
______ Confirmed acute schistosomiasis

Eggs, CAA, PCR, or Seroconversion

Optional*
______ Probable acute schistosomiasis

Serology or CCA

Optional*
______ Possible acute schistosomiasis
______ Cannot be defined as acute schistosomiasis, but high epidemiological suspicion

(e.g. cluster contact)

CAA=circulating anodic antigen. CCA=circulating cathodic antigen.

* Clinical criteria can be absent if there is absolutely no evidence of previous exposure.

Complete case definitions can be found in on Tamarozzi F, Mazzi C, Antinori S, et al. Consensus definitions in imported human schistosomiasis: a GeoSentinel and TropNet Delphi study. Lancet Infect Dis. 2024

[Attachment: "Consensus definitions in imported human schistosomiasis.pdf"]

Comments on initial case definition

____

1st visit: Treatment

□□ Treatment Registration - Important Instructions

This form is repeatable. If more than one administration of the same drug was prescribed (e.g., repeated praziquantel course or more than one corticosteroid course), you should create a new form entry.

□□ How to repeat the form:

You can create a new instance of this form in either of the following ways:

- From the Record Home Page, click the □ button located next to the status icon of the treatment form.
- Within the form itself, click the "+ Add new" button found at the top of the page under the "Current Instance" menu.

Previous treatment

Had the patient received any previous treatment before arriving to your center?	○ Yes<br>○ No<br>○ Unknown
Do you know which treatment(s) had the patient previously received?	☐ Empirical antiparasitic treatment<br>☐ Empirical antibiotic treatment<br>☐ Symptomatic treatment<br>☐ Other (specify)<br>☐ Unknown
Do you know which antiparasitic treatment(s)?	☐ Albendazole<br>☐ Mebendazole<br>☐ Ivermectin<br>☐ Praziquantel<br>☐ Metronidazole or tinidazole<br>☐ Antimalarial treatment<br>☐ Other<br>☐ Unknown
Which antimalarial treatment(s)?	☐ Artemether + lumefantrine<br>☐ Artesunate + amodiaquine<br>☐ Dihydroartemisinin + piperaquine<br>☐ Artesunate + mefloquine<br>☐ Artesunate + sulfadoxine-pyrimethamine<br>☐ Intravenous artesunate<br>☐ Intramuscular artemether<br>☐ Quinine + doxycycline<br>☐ Quinine + clindamycin<br>☐ Other (specify)

Do you know which antibiotic(s)?	☐ Anti-staphylococcal penicillin (e.g., cloxacillin, nafcillin)<br>☐ Broad-spectrum penicillin (e.g., amoxicillin-clavulanate, piperacillin-tazobactam)<br>☐ First/second generation cephalosporin (e.g., cefazolin, cephalexin, cefuroxime)<br>☐ Other beta-lactam antibiotic<br>☐ Macrolide (e.g., azithromycin, clarithromycin)<br>☐ Quinolone (e.g., moxifloxacin, levofloxacin)<br>☐ Other (specify)<br>☐ Unknown
Do you know which symptomatic treatment(s)?	☐ Paracetamol<br>☐ NSAIDs (e.g., ibuprofen, naproxen, celecoxib)<br>☐ Antihistamines (e.g., loratadine, diphenhydramine)<br>☐ Topical corticosteroids (e.g., hydrocortisone, betamethasone)<br>☐ Systemic corticosteroids (e.g., prednisone, dexamethasone)<br>☐ Antiperistaltics (e.g., loperamide, racecadotril)<br>☐ Antiemetics (e.g., metoclopramide, ondansetron)<br>☐ Gastric acid suppressants (e.g., proton pump inhibitors like omeprazole, or H2 blockers like ranitidine or famotidine)<br>☐ Other (specify)<br>☐ Unknown
Please specify any other treatments the patient received before arriving at your center	______________________________

Treatment of acute schistosomiasis remains controversial and inconsistent even in reference centers, as no evidence supports any specific regimen at this moment. There is some consensus that corticosteroids are usually necessary to control the symptoms whenever they develop, and that praziquantel (that is effective only against adult worms) should be administered at least once after 8-12 weeks and earlier also in case of symptoms. However, there is so far no evidence-based recommendation on the optimal timing, dosage, duration, and combination of both drugs.

Based on limited observational evidence, the regimen proposed/suggested at the Institute of Tropical Medicine, Antwerp, Belgium is as follows:

Symptomatic patient

Time after exposure/infection Recommended treatment
< 6 weeks Methylprednisolone 0.5 mg/kg/day (or equivalent dose with other corticosteroid)

x 3 days

To be prolonged/repeated if unsatisfactory control of inflammatory symptoms.

>6 weeks Praziquantel 40 mg/kg single dose

followed after 2-4 hours* by

Methylprednisolone 0.5 mg/kg/day (or equivalent dose with other corticosteroid)

x 3 days

To be prolonged/repeated if unsatisfactory control of inflammatory symptoms

* Note that this interval allows the concentration peak of praziquantel within 1-2 hours after administration, with no drug interaction

A second 40 mg/kg single-dose course of praziquantel at week 12 after infection should be administered to clear all juvenile worms which have meanwhile become adults

---

Treatment of acute schistosomiasis remains controversial and inconsistent even in reference centers, as no evidence supports any specific regimen at this moment. There is some consensus that corticosteroids are usually necessary to control the symptoms whenever they develop, and that praziquantel (that is effective only against adult worms) should be administered at least once after 8-12 weeks and earlier also in case of symptoms. However, there is so far no evidence-based recommendation on the optimal timing, dosage, duration, and combination of both drugs.

Based on limited observational evidence, the regimen proposed/suggested at the Institute of Tropical Medicine, Antwerp, Belgium is as follows:

Asymptomatic patient

Time after exposure/infection Recommended treatment

< 6 weeks Abstention

>6 weeks Praziquantel 40 mg/kg single dose

followed after 2-4 hours* by

Methylprednisolone 0.5 mg/kg/day (or equivalent dose with other corticosteroid)

single dose

To be prolonged/repeated if clinical exacerbation

* Note that this interval allows the concentration peak of praziquantel within 1-2 hours after administration, with no drug interaction

A second 40 mg/kg single-dose course of praziquantel at week 12 after infection should be administered to clear all juvenile worms which have meanwhile become adults

---

Which treatment did you prescribe at the initial visit? (more than one is possible)

☐ Praziquantel
☐ Corticosteroids
☐ Observation (no treatment, 'wait and see')
☐ Other

Please specify other treatment

____

Please specify dose, frequency and duration (in days)

____

Praziquantel administration

Praziquantel start date

____

Praziquantel administration

◯ 40mg/kg single dose
◯ Two separate doses of 20 mg/kg each, administered 2 hours apart. Followed by corticosteroids.
◯ 20mg/kg/12h for 1 day
◯ 30mg/kg/12h for 1 day
◯ 20mg/kg/12h for 2 days
◯ 30mg/kg/12h for 2 days
◯ Other

Praziquantel dose (mg/Kg)

____

Praziquantel frequency

____
(every X hours, for example)

Total duration of praziquantel (in days)

____

Corticosteroid administration

Corticosteroid start date

____

Corticosteroid type

◯ Methylprednisolone
◯ Prednisolone
◯ Prednisone
◯ Dexamethasone
◯ Other

Initial corticosteroid dose (mg/kg)

____

Methylprednisolone-equivalent dose (mg/kg)

____

Corticosteroid frequency

◯ Once a day
◯ Every 12h
◯ Every 8h
◯ Other

Specify corticosteroid frequency

____

Total duration of corticosteroid therapy (in days)

____

Was a step-down corticosteroid taper used?

◯ Yes
◯ No
◯ Unknown

Step-down corticosteroid taper schedule

Step	Dose (mg/day)	MTP equivalent (mg/day)	Duration (days)
Step 1	______	______	______
Step 2	______	______	______
Step 3	______	______	______
Step 4	______	______	______
Step 5	______	______	______

Why did the patient receive corticosteroids despite being asymptomatic at initial assessment?

____

Side effects

Any side effects due to treatment?

- ◯ Yes
- ◯ No
- ◯ Unknown

Date of side effects onset

____

Which type(s) of side effects?

- ☐ Systemic/general
- ☐ Neurological
- ☐ Dermatologic
- ☐ Gastrointestinal
- ☐ Other

(Select all that apply)

Which general/systemic side effects?

- ☐ Fever
- ☐ Malaise
- ☐ Fatigue
- ☐ Myalgia
- ☐ Paradoxical reaction
- ☐ Eosinophilia
- ☐ Elevated ALT/AST
- ☐ Other

(Paradoxical reaction refers to temporary worsening of symptoms after treatment, due to immune reaction against dying parasites (not drug toxicity).)

Which neurological side effects?

- ☐ Headache
- ☐ Dizziness
- ☐ Drowsiness
- ☐ Vertigo
- ☐ Seizure
- ☐ Other

Which dermatological side effects?

- ☐ Urticaria
- ☐ Angioedema
- ☐ Rash
- ☐ Pruritus
- ☐ Other

Which gastrointestinal side effects?

- ☐ Nausea
- ☐ Vomiting
- ☐ Abdominal pain
- ☐ Diarrhea
- ☐ Anorexia
- ☐ Other

Please specify other side effect(s)

____

Severity of the most significant side effect	◯ Mild - no interference with activity<br>◯ Moderate - some interference, no hospitalization<br>◯ Severe - prevents daily activity, may require hospitalization<br>◯ Life-threatening - urgent intervention required<br>◯ Death<br>◯ Unknown
Date of side effect resolution	__________________________________
Date of death	__________________________________
Comments	____

F-U visit: Clinical features

Was a (new) follow-up visit done?	◯ Yes<br>◯ No<br>◯ Unknown
Reason follow-up visit was not performed	☐ Patient did not attend follow-up visit<br>☐ Patient declined further visits<br>☐ Patient asymptomatic and treatment instructions given at previous visit, follow-up deemed unnecessary<br>☐ Patient left the country / returned to home country<br>☐ Patient referred to another center<br>☐ Other (specify)<br>☐ Unknown
Specify other reason why follow-up visit was not performed	__________________________________
Date of follow-up visit	__________________________________
How was this visit done?	◯ In person<br>◯ Phone call<br>◯ Video call (Zoom, Hangouts, WA...)<br>◯ Email<br>◯ Unknown
Did the patient have symptoms at this visit?	◯ Yes<br>◯ No<br>◯ Unknown
Specify	☐ Persistence of all previous symptoms<br>☐ Partial resolution of symptoms<br>☐ Recurrence of symptoms after initial improvement<br>☐ New symptoms onset<br>☐ Unknown
Specify	◯ All previous symptoms resolved<br>◯ Patient was always asymptomatic<br>◯ Unknown
Date of symptom resolution	__________________________________

Which symptom(s) persisted?	☐ Fever<br>☐ Chills<br>☐ Fatigue<br>☐ Myalgias/arthralgias<br>☐ Headache<br>☐ Abdominal pain<br>☐ Nausea/vomiting<br>☐ Diarrhea<br>☐ Dyspnea<br>☐ Cough<br>☐ Urticaria<br>☐ Angioedema<br>☐ Other
Date of symptom recurrence	__________________________________
Which symptom(s) recurred?	☐ Fever<br>☐ Chills<br>☐ Fatigue<br>☐ Myalgias/arthralgias<br>☐ Headache<br>☐ Abdominal pain<br>☐ Nausea/vomiting<br>☐ Diarrhea<br>☐ Dyspnea<br>☐ Cough<br>☐ Urticaria<br>☐ Angioedema<br>☐ Other
If other, specify	__________________________________
Date of new symptoms onset	__________________________________
Which new symptom(s) appeared?	☐ Fever<br>☐ Chills<br>☐ Fatigue<br>☐ Myalgias/arthralgias<br>☐ Headache<br>☐ Abdominal pain<br>☐ Nausea/vomiting<br>☐ Diarrhea<br>☐ Dyspnea<br>☐ Cough<br>☐ Urticaria<br>☐ Angioedema<br>☐ Other
If other, specify	__________________________________

Confidential

F-U visit: Basic lab results

▯▯ Laboratory Results - Important Instructions

Please provide laboratory results that are closest in time to the clinical visit for which this form is being completed.

▯▯ Which results to enter:

Select values from the laboratory test(s) performed on the same day as the visit whenever possible.
If same-day results are not available, choose the nearest available lab test performed before or after the visit (ideally within ±7 days).

▯▯ Why this matters:

Using results closest to the clinical assessment ensures accurate correlation between clinical status and laboratory findings.

Was a blood analysis done?	◯ Yes<br>◯ No<br>◯ Unknown
Date of lab test	__________________________________<br>(Enter the date of the lab test performed on the same day as the clinical visit, or the nearest available test within ±7 days.)

Main Lab results

Hemoglobin value	__________________________________
Hemoglobin values unit	◯ g/dL<br>◯ Other
Hemoglobin unit - specify other	__________________________________
WBC count	__________________________________
WBC count unit	◯ x10^9/L<br>◯ Other
WBC count unit - specify other	__________________________________
Eosinophil count	__________________________________
EOS count unit	◯ cells/µL<br>◯ Other
EOS count unit - specify other	__________________________________

Platelet count	__________________________________
Platelet count unit	◯ $\times 10^9$/L<br>◯ Other
Platelet count unit - specify other	__________________________________
IgE value	__________________________________
IgE unit	◯ IU/mL<br>◯ kU/L<br>◯ Other
IgE unit - specify other	__________________________________
C-reactive protein value	__________________________________
C-reactive protein unit	◯ mg/L<br>◯ Other
C-reactive protein unit - specify other	__________________________________

Other lab results

Any other baseline lab abnormalities found?	◯ Yes<br>◯ No
Which one(s)?	☐ Elevated ALT<br>☐ Elevated AST<br>☐ Elevated GGT<br>☐ Elevated Alkaline Phosphatase<br>☐ Elevated LDH<br>☐ Elevated Creatinine<br>☐ Elevated Urea<br>☐ Other (specify)
Which other lab abnormalities?	__________________________________
Were any of the following tests performed?	☐ Urinalysis<br>☐ Fecal occult blood test (FOBT)
Were any of the following abnormalities present?	☐ Proteinuria<br>☐ Hematuria<br>☐ Leukocyturia<br>☐ Other
Please describe other urinalysis abnormalities	__________________________________

Proteinuria - Type of assessment	☐ Dipstick (semi-quantitative)<br>☐ Quantitative (lab-based)<br>☐ Unknown
Proteinuria (dipstick result)	◯ Negative<br>◯ Trace<br>◯ 1+<br>◯ 2+<br>◯ 3+<br>◯ 4+
Proteinuria (quantitative result)	________________________
Proteinuria unit	◯ mg/dL<br>◯ mg/day<br>◯ g/day<br>◯ Protein-to-creatinine ratio (mg/g)<br>◯ Other
Specify other proteinuria unit	________________________
Hematuria (graded by dipstick or microscopy)	◯ Microscopic (< 10 RBCs/µL or trace)<br>◯ Mild (10-50 RBCs/µL or 1+)<br>◯ Moderate (50-250 RBCs/µL or 2+)<br>◯ Gross hematuria (>250 RBCs/µL or ≥3+)<br>◯ Unknown
Leukocyturia (dipstick or microscopy result)	◯ Trace / Few WBCs (< 10/µL)<br>◯ Moderate (10-50 WBCs/µL or 1-2+)<br>◯ Marked (>50 WBCs/µL or ≥3+)<br>◯ Unknown
Fecal occult blood test result?	◯ Positive<br>◯ Negative<br>◯ Indeterminate<br>◯ Invalid<br>◯ Unknown
Other comments	______________________________

F-U visit: Schisto-specific tests

Schisto-specific tests

Please fill in the following table as detailed as possible.

⚠

Important Instructions for this section:

1

 For each test, please ensure that only one option is selected. If multiple options are selected in a matrix row by mistake, use the "reset" button to deselect the incorrect choice.

2

 For all results marked as Positive, Negative, or Indeterminate, please provide the numerical value of the test result whenever available (e.g., titer, egg count, Ct value, etc) in the box that appears to the right of each row.

⚠

Note on Diagnosis of Acute Schistosomiasis:

Diagnosis of acute schistosomiasis is very challenging, especially at first presentation for symptomatic patients. Microscopic examination to detect eggs in urine and/or stool is often negative because the sensitivity of this method, which is usually limited for schistosomiasis, is even lower as eggs may become detectable only 4 to 6 weeks after exposure/infection. Similarly, serological methods become usually positive a few weeks (up to 2-3 months) after infection, as they are often based on adult worm antigens.

For this reason, both methods must be either performed 8-12 weeks after exposure to screen asymptomatic exposed individuals or repeated at that time if they were negative at initial workup.

In general, if there is a strong clinical suspicion in symptomatic patients, therapy should be initiated before full confirmation.

Of note, novel methods such as PCR-based or Circulating Anionic Antigen (CAA)-based assays in serum become positive more rapidly, within the 6 first weeks after infection, but are usually not available in the clinical practice. Very few reference/research laboratories in Europe could perform these tests at this moment.

Was a new schisto-specific test done?

◯ Yes
◯ No
◯ Unknown

Date of Schisto-specific test

____

Positive Negative Indeterminate Not done
Antibody serology
IHA ______ ______ ______ ______ ______
ELISA ______ ______ ______ ______ ______
Other ______ ______ ______ ______ ______ ______
Egg detection
Eggs in urine ______ ______ ______ ______
Eggs in stool ______ ______ ______ ______
Antigenic tests
POC CCA ______ ______ ______ ______ ______
LFA CAA (lateral flow assay) ______ ______ ______ ______ ______
Other ______ ______ ______ ______ ______ ______
PCR
Serum Dra1 ______ ______ ______ ______

Serum Sm1-7 ______ ______ ______ ______

Urine 28S rRNA

______ ______ ______ ______

Stool 28S rRNA

______ ______ ______ ______

Other ______

______ ______ ______ ______

Further comments

____

(Please use this section to further explain your choice and what your institution uses)

F-U Visit Other relevant results

Was any imaging test done in this visit?

◯ Yes
◯ No
◯ Unknown

Imaging tests

Which imaging test(s) were performed?

☐ Chest X-ray
☐ Abdominal ultrasound
☐ Chest CT scan
☐ Abdominal CT scan
☐ Brain CT scan
☐ Brain MRI
☐ Spinal cord MRI
☐ Hepatic MRI
☐ Echocardiogram
☐ PET-CT scan
☐ Other
(Check all that apply)

Specify which other imaging test was performed

____

Date of chest X-ray

____

Chest X-ray - Main Findings

☐ Normal
☐ Pulmonary infiltrates
☐ Interstitial opacities
☐ Pleural effusion
☐ Hilar or mediastinal lymphadenopathy
☐ Cardiomegaly
☐ Other (specify)

Specify other abnormal findings on chest X-ray

____

Date of abdominal US

____

Abdominal Ultrasound - Main Findings

☐ Normal
☐ Hepatosplenomegaly
☐ Splenomegaly only
☐ Hyperechogenic liver
☐ Enlarged periportal lymph nodes
☐ Ascites
☐ Other (specify)

Specify other abnormal findings on abdominal US

____

Date of chest CT scan

____

Chest CT scan - Main Findings	☐ Normal<br>☐ Pulmonary nodules<br>☐ Ground-glass opacities<br>☐ Interstitial infiltrates<br>☐ Pleural effusion<br>☐ Enlarged mediastinal or hilar lymph nodes<br>☐ Other (specify)
Specify other abnormal findings on chest CT scan	__________________________________
Date of abdominal CT scan	__________________________________
Abdominal CT scan - Main Findings	☐ Normal<br>☐ Hepatosplenomegaly<br>☐ Splenomegaly only<br>☐ Enlarged abdominal or periportal lymph nodes<br>☐ Focal hepatic lesions (e.g., granulomas or nodules)<br>☐ Ascites<br>☐ Other (specify)
Specify other abnormal findings on abdominal CT scan	__________________________________
Date of brain CT scan	__________________________________
Please describe any abnormal imaging findings on brain CT	__________________________________
Date of brain MRI	__________________________________
Please describe any abnormal imaging findings on brain MRI	__________________________________
Date of spinal cord MRI	__________________________________
Please describe any abnormal imaging findings on spinal cord MRI	__________________________________
Date of hepatic MRI	__________________________________
Please describe any abnormal imaging findings on hepatic MRI	__________________________________
Date of echocardiogram	__________________________________
Please describe any abnormal imaging findings on echocardiogram	__________________________________
Date of PET-CT scan	__________________________________

Please describe any abnormal imaging findings on PET-CT scan	________________________
Date of other imaging test	________________________
Please describe any abnormal imaging findings on other imaging tests	________________________

Other infections

Were other concomitant infections diagnosed?	◯ Yes<br>◯ No<br>◯ Unknown
Which one(s)?	☐ Malaria<br>☐ Dengue<br>☐ Strongyloidiasis<br>☐ Leptospirosis<br>☐ Other
Other (specify)	________________________
Comments on other relevant investigations or diagnosis	______________________________

F-U visit: Case definition

Did the case classification change from the previous visit based on new clinical or laboratory data?

- ◯ Yes
- ◯ No
- ◯ FU visit not done
- ◯ Unknown

How would you define the case at this visit?

Choose one Case classification Epidemiological criteria Microbiological criteria Clinical criteria
______ Confirmed acute schistosomiasis

Eggs, CAA, PCR, or Seroconversion

Optional*
______ Probable acute schistosomiasis

Serology or CCA

Optional*
______ Possible acute schistosomiasis
______ Cannot be defined as acute schistosomiasis, but high epidemiological suspicion

(e.g. cluster contact)

CAA=circulating anodic antigen. CCA=circulating cathodic antigen.

* Clinical criteria can be absent if there is absolutely no evidence of previous exposure.

Complete case definitions can be found in on Tamarozzi F, Mazzi C, Antinori S, et al. Consensus definitions in imported human schistosomiasis: a GeoSentinel and TropNet Delphi study. Lancet Infect Dis. 2024

[Attachment: "Consensus definitions in imported human schistosomiasis.pdf"]

Comments on follow-up case definition

____

F-U visit: Treatment

□□ Treatment Registration - Important Instructions

This form is repeatable. If more than one administration of the same drug was prescribed (e.g., repeated praziquantel course or more than one corticosteroid course), you should create a new form entry.

□□ How to repeat the form:

You can create a new instance of this form in either of the following ways:

- From the Record Home Page, click the □ button located next to the status icon of the treatment form.
- Within the form itself, click the "+ Add new" button found at the top of the page under the "Current Instance" menu.

Was treatment administered/prescribed in a follow-up visit?
○ Yes
○ No

Why did the patient receive treatment in this visit?
☐ Persistent symptoms
☐ Clinical relapse after initial improvement
☐ Eosinophilia rebound
☐ Seroconversion or diagnostic confirmation
☐ Persistance of positive antigen result
☐ Persistance of positive PCR result
☐ First praziquantel dose after decided to ''wait and see'' in the first visit
☐ Second praziquantel dose to clear all juvenile worms which have meanwhile become adults
☐ Other (specify)

Specify other reason why the patient received a new treatment course
____

Treatment of acute schistosomiasis remains controversial and inconsistent even in reference centers, as no evidence supports any specific regimen at this moment. There is some consensus that corticosteroids are usually necessary to control the symptoms whenever they develop, and that praziquantel (that is effective only against adult worms) should be administered at least once after 8-12 weeks and earlier also in case of symptoms. However, there is so far no evidence-based recommendation on the optimal timing, dosage, duration, and combination of both drugs.

Based on limited observational evidence, the regimen proposed/suggested at the Institute of Tropical Medicine, Antwerp, Belgium is as follows:

Symptomatic patient

Time after exposure/infection Recommended treatment
< 6 weeks Methylprednisolone 0.5 mg/kg/day (or equivalent dose with other corticosteroid)

x 3 days

To be prolonged/repeated if unsatisfactory control of inflammatory symptoms.

>6 weeks Praziquantel 40 mg/kg single dose

followed after 2-4 hours* by

Methylprednisolone 0.5 mg/kg/day (or equivalent dose with other corticosteroid)

x 3 days

To be prolonged/repeated if unsatisfactory control of inflammatory symptoms

* Note that this interval allows the concentration peak of praziquantel within 1-2 hours after administration, with no drug interaction

Asymptomatic patient

Time after exposure/infection Recommended treatment
< 6 weeks Abstention

>6 weeks Praziquantel 40 mg/kg single dose

followed after 2-4 hours* by

Methylprednisolone 0.5 mg/kg/day (or equivalent dose with other corticosteroid)

single dose

To be prolonged/repeated if clinical exacerbation

* Note that this interval allows the concentration peak of praziquantel within 1-2 hours after administration, with no drug interaction

A second 40 mg/kg single-dose course of praziquantel at week 12 after infection should be administered to clear all juvenile worms which have meanwhile become adults

---

Which one?

- ☐ Praziquantel
- ☐ Corticosteroid
- ☐ Other

Please specify other treatment

____

Please specify dose, frequency and duration (in days)

____

Praziquantel administration

Praziquantel start date

____

Praziquantel administration

- ◯ 40mg/kg single dose
- ◯ Two separate doses of 20 mg/kg each, administered 2 hours apart. Followed by corticosteroids.
- ◯ 20mg/kg/12h for 1 day
- ◯ 30mg/kg/12h for 1 day
- ◯ 20mg/kg/12h for 2 days
- ◯ 30mg/kg/12h for 2 days
- ◯ Other

Praziquantel dose (mg/Kg)

____

Praziquantel frequency

____
(every X hours, for example)

Total duration of praziquantel (in days)

____

Corticosteroid administration

Corticosteroid start date

____

Corticosteroid type

○ Methylprednisolone
○ Prednisolone
○ Prednisone
○ Dexamethasone
○ Other

Initial corticosteroid dose (mg/kg)

____

Methylprednisolone-equivalent dose (mg/kg)

____

Corticosteroid frequency

○ Once a day
○ Every 12h
○ Every 8h
○ Other

Specify corticosteroid frequency

____

Total duration of corticosteroid therapy (in days)

____

Was a step-down corticosteroid taper used?

○ Yes
○ No
○ Unknown

Step-down corticosteroid taper schedule

Step Dose (mg/day) MTP equivalent (mg/day) Duration (days)
Step 1 ______ ______ ______
Step 2 ______ ______ ______
Step 3 ______ ______ ______
Step 4 ______ ______ ______
Step 5 ______ ______ ______

Side effects

Any side effects due to treatment?	◯ Yes<br>◯ No<br>◯ Unknown
Date of side effects onset	________________________
Which type(s) of side effects?	☐ Systemic/general<br>☐ Neurological<br>☐ Dermatologic<br>☐ Gastrointestinal<br>☐ Other<br>(Select all that apply)
Which general/systemic side effects?	☐ Fever<br>☐ Malaise<br>☐ Fatigue<br>☐ Myalgia<br>☐ Paradoxical reaction<br>☐ Eosinophilia<br>☐ Elevated ALT/AST<br>☐ Other<br>(Paradoxical reaction refers to temporary worsening of symptoms after treatment, due to immune reaction against dying parasites (not drug toxicity).)
Which neurological side effects?	☐ Headache<br>☐ Dizziness<br>☐ Drowsiness<br>☐ Vertigo<br>☐ Seizure<br>☐ Other
Which dermatologic side effects?	☐ Urticaria<br>☐ Angioedema<br>☐ Rash<br>☐ Pruritus<br>☐ Other
Which gastrointestinal side effects?	☐ Nausea<br>☐ Vomiting<br>☐ Abdominal pain<br>☐ Diarrhea<br>☐ Anorexia<br>☐ Other
Please specify other side effect(s)	________________________
Severity of the most significant side effect	◯ Mild - no interference with activity<br>◯ Moderate - some interference, no hospitalization<br>◯ Severe - prevents daily activity, may require hospitalization<br>◯ Life-threatening - urgent intervention required<br>◯ Death<br>◯ Unknown

Date of side effect resolution

____

Date of death

____

Comments

____

Outcome

Date of outcome assessment

____
(Specify the date of last contact with the patient.)

How would you finally define the case?

Choose one Case classification Epidemiological criteria Microbiological criteria Clinical criteria
______ Confirmed acute schistosomiasis

Eggs, CAA, PCR, or Seroconversion

Optional*
______ Probable acute schistosomiasis

Serology or CCA

Optional*
______ Possible acute schistosomiasis
______ Cannot be defined as acute schistosomiasis, but high epidemiological suspicion

(e.g. cluster contact)

CAA=circulating anodic antigen. CCA=circulating cathodic antigen.

* Clinical criteria can be absent if there is absolutely no evidence of previous exposure.

Complete case definitions can be found in on Tamarozzi F, Mazzi C, Antinori S, et al. Consensus definitions in imported human schistosomiasis: a GeoSentinel and TropNet Delphi study. Lancet Infect Dis. 2024

[Attachment: "Consensus definitions in imported human schistosomiasis.pdf"]

Outcome

☐ Cure
☐ Relapse
☐ Hospitalization
☐ Death
☐ Lost to follow-up
(Relapse is defined as the recurrence of symptoms or laboratory abnormalities after initial improvement or cure. If a patient experienced a relapse but had resolution of symptoms at the time of final assessment, both "Cure" and "Relapse" should be selected.)

Reason for lost to follow-up

☐ Patient did not attend follow-up visit
☐ Patient declined further visits
☐ Patient left the country / returned to home country
☐ Patient referred to another center
☐ Other (specify)
☐ Unknown

Specify other reason why the patient was lost to follow-up

____

How many relapses (worsening after symptoms resolution or improvement) did the patient have?

____
(Enter the number of distinct episodes of symptom worsening after a period of resolution or improvement.)

Date of death

____

Cure assessment

What elements led you to consider the patient as cured?

- ☐ Symptom resolution
- ☐ Normalization of basic laboratory (e.g. eosinophil count, CRP)
- ☐ Negativization of schisto-specific tests (e.g., CAA, PCR, serology)
- ☐ Other

Please specify how you assessed cure

____

Date of symptom resolution

____

Date of basic laboratory normalization

____

Which one(s)?

- ☐ Serology negativization
- ☐ Significant serological titer reduction
- ☐ Antigen test negativization
- ☐ PCR negativization
- ☐ Other

Date of serology negativization

____

Date of significant serological titer reduction

____

Date of antigen test negativization

____

Date of PCR negativization

____

Please specify

____

Hospitalization

Which was the reason for hospitalization?

☐ Diagnostic workup for fever in a returning traveler
☐ Severity of clinical condition at presentation
☐ Treatment administration (e.g., intravenous therapy, monitoring)
☐ Severe adverse event or side effect
☐ Other

Please explain other reason for hospitalization

____

Date of hospitalization

____

How long was the patient hospitalized (days)?

____

Other complications and additional investigations

Any other complication during the course of the disease?

◯ Yes
◯ No
◯ Unknown

Please specify other complications

____

Any other relevant additional investigation during the course of the disease?

◯ Yes
◯ No
◯ Unknown

Please specify

____

Comment(s)

____

□□ Thank you for your valuable contribution

Thank you very much for having spent some time in entering those important data. We are confident that this collaborative effort will generate key information to improve the future clinical guidance of, and care of patients with, acute schistosomiasis.

TEMPLATE Schisto diagnostic lab results 2

Antibody serology	☐ IHA ______<br>☐ ELISA ______<br>☐ others
Egg detection	☐ Eggs in urine<br>☐ Eggs in stool
Antigenic tests	☐ POC CCA<br>☐ LFA CAA (lateral flow essay)<br>☐ Other
PCR	☐ urine<br>☐ serum Dra1<br>☐ serum Sm1-7<br>☐ stool<br>☐ other

Antibody serology

	Positive	Negative	Not done
IHA	◯	◯	◯
ELISA	◯	◯	◯
Others ______	◯	◯	◯

____

Positive Negative Not done
Antibody serology
IHA ______ ______ ______ ______
ELISA ______ ______ ______ ______
Other ______ ______ ______ ______ ______
Egg detection
Eggs in urine ______ ______ ______ ______
Eggs in stool ______ ______ ______ ______
Antigenic tests
POC CCA ______ ______ ______ ______
LFA CCA (lateral flow assay) ______ ______ ______ ______
Other ______ ______ ______ ______ ______
PCR
Serum Dra1 ______ ______ ______ ______
Serum Sm1-7 ______ ______ ______ ______
Urine 28S rRNA

______ ______ ______ ______
Stool 28S rRNA

______ ______ ______ ______
Other ______

______ ______ ______ ______

Was a second course of corticosteroids administered?	◯ Yes<br>◯ No

2nd corticosteroid course type	◯ Methylprednisolone<br>◯ Prednisolone<br>◯ Prednisone<br>◯ Dexamethasone<br>◯ Other
2nd corticosteroid course start date	__________________________________
2nd corticosteroid course dose (mg/kg)	__________________________________
2nd methylprednisolone-equivalent dose (mg/kg)	__________________________________
2nd corticosteroid course frequency	◯ Once a day<br>◯ Every 12h<br>◯ Every 8h<br>◯ Other
Specify second corticosteroid course frequency	__________________________________
Duration of 2nd corticosteroid course (in days)	__________________________________
Was a second corticosteroid course administered?	◯ Yes<br>◯ No
2nd corticosteroid course start date	__________________________________
2nd corticosteroid course type	◯ Methylprednisolone<br>◯ Prednisolone<br>◯ Prednisone<br>◯ Dexamethasone<br>◯ Other
2nd corticosteroid dose (mg/kg)	__________________________________
2nd methylprednisolone-equivalent dose (mg/kg)	__________________________________
2nd corticosteroid course frequency	◯ Once a day<br>◯ Every 12h<br>◯ Every 8h<br>◯ Other
Specify other frequency for 2nd corticosteroid course	__________________________________
Duration of 2nd corticosteroid course (in days)	__________________________________

If yes, specify side effects:	☐ Nausea<br>☐ Vomiting<br>☐ Headache<br>☐ Dizziness<br>☐ Swelling<br>☐ Other<br>(Select all that apply)
Where?	______________________________
