# Supplementary material for: Tropistry: A registry-based modular platform to improve care of neglected tropical diseases in nonendemic settings—Study protocol with two targeted conditions: acute schistosomiasis and cutaneous/mucocutaneous leishmaniasis
Source: PLoS One. 2025 Oct 30;20(10):e0335032. doi: 10.1371/journal.pone.0335032 (PMC12574823; doi:10.1371/journal.pone.0335032)
Supplement: S2 File — (PDF) [file pone.0335032.s003.pdf]

Demographics

Record ID	__________________________________
Sex at birth	◯ Male<br>◯ Female<br>◯ Other
Year of birth	__________________________________

Country of birth

- ◯ Unknown
- ◯ Afghanistan
- ◯ Åland Islands
- ◯ Albania
- ◯ Algeria
- ◯ American Samoa
- ◯ Andorra
- ◯ Angola
- ◯ Anguilla
- ◯ Antarctica
- ◯ Antigua and Barbuda
- ◯ Argentina
- ◯ Armenia
- ◯ Aruba
- ◯ Australia
- ◯ Austria
- ◯ Azerbaijan
- ◯ Bahamas
- ◯ Bahrain
- ◯ Bangladesh
- ◯ Barbados
- ◯ Belarus
- ◯ Belgium
- ◯ Belize
- ◯ Benin
- ◯ Bermuda
- ◯ Bhutan
- ◯ Bolivia
- ◯ Bonaire, Sint Eustatius and Saba
- ◯ Bosnia and Herzegovina
- ◯ Botswana
- ◯ Bouvet Island
- ◯ Brazil
- ◯ British Indian Ocean Territory
- ◯ Brunei Darussalam
- ◯ Bulgaria
- ◯ Burkina Faso
- ◯ Burundi
- ◯ Cabo Verde
- ◯ Cambodia
- ◯ Cameroon
- ◯ Canada
- ◯ Cayman Islands
- ◯ Central African Republic
- ◯ Chad
- ◯ Chile
- ◯ China
- ◯ Christmas Island
- ◯ Cocos (Keeling) Islands
- ◯ Colombia
- ◯ Comoros
- ◯ Congo-Brazzaville
- ◯ Congo (DRC)
- ◯ Cook Islands
- ◯ Costa Rica
- ◯ Côte d'Ivoire
- ◯ Croatia
- ◯ Cuba
- ◯ Curaçao
- ◯ Cyprus
- ◯ Czechia
- ◯ Denmark
- ◯ Djibouti
- ◯ Dominica
- ◯ Dominican Republic
- ◯ Ecuador
- ◯ Egypt
- ◯ El Salvador
- ◯ Equatorial Guinea

◯ Eritrea
◯ Estonia
◯ Ethiopia
◯ Falkland Islands
◯ Faroe Islands
◯ Fiji
◯ Finland
◯ France
◯ French Guiana
◯ French Polynesia
◯ French Southern Territories
◯ Gabon
◯ Gambia
◯ Georgia
◯ Germany
◯ Ghana
◯ Gibraltar
◯ Greece
◯ Greenland
◯ Grenada
◯ Guadeloupe
◯ Guam
◯ Guatemala
◯ Guernsey
◯ Guinea
◯ Guinea-Bissau
◯ Guyana
◯ Haiti
◯ Heard Island and McDonald Islands
◯ Holy See
◯ Honduras
◯ Hong Kong
◯ Hungary
◯ Iceland
◯ India
◯ Indonesia
◯ Iran
◯ Iraq
◯ Ireland
◯ Isle of Man
◯ Israel
◯ Italy
◯ Jamaica
◯ Japan
◯ Jersey
◯ Jordan
◯ Kazakhstan
◯ Kenya
◯ Kiribati
◯ Kuwait
◯ Kyrgyzstan
◯ Laos
◯ Latvia
◯ Lebanon
◯ Lesotho
◯ Liberia
◯ Libya
◯ Liechtenstein
◯ Lithuania
◯ Luxembourg
◯ Macao
◯ Macedonia
◯ Madagascar
◯ Malawi
◯ Malaysia
◯ Maldives
◯ Mali
◯ Malta
◯ Marshall Islands
◯ Martinique
◯ Mauritania

◯ Mauritius
◯ Mayotte
◯ Mexico
◯ Micronesia
◯ Moldova
◯ Monaco
◯ Mongolia
◯ Montenegro
◯ Montserrat
◯ Morocco
◯ Mozambique
◯ Myanmar
◯ Namibia
◯ Nauru
◯ Nepal
◯ Netherlands
◯ New Caledonia
◯ New Zealand
◯ Nicaragua
◯ Niger
◯ Nigeria
◯ Niue
◯ Norfolk Island
◯ North Korea
◯ Northern Mariana Islands
◯ Norway
◯ Oman
◯ Pakistan
◯ Palau
◯ Palestine
◯ Panama
◯ Papua New Guinea
◯ Paraguay
◯ Peru
◯ Philippines
◯ Pitcairn
◯ Poland
◯ Portugal
◯ Puerto Rico
◯ Qatar
◯ Réunion
◯ Romania
◯ Russian Federation
◯ Rwanda
◯ Saint Barthélemy
◯ Saint Helena, Ascension and Tristan da Cunha
◯ Saint Kitts and Nevis
◯ Saint Lucia
◯ Saint Martin (French part)
◯ Saint Pierre and Miquelon
◯ Saint Vincent and the Grenadines
◯ Samoa
◯ San Marino
◯ Sao Tome and Principe
◯ Saudi Arabia
◯ Senegal
◯ Serbia
◯ Seychelles
◯ Sierra Leone
◯ Singapore
◯ Sint Maarten (Dutch part)
◯ Slovakia
◯ Slovenia
◯ Solomon Islands
◯ Somalia
◯ South Africa
◯ South Georgia and the South Sandwich Islands
◯ South Korea
◯ South Sudan
◯ Spain
◯ Sri Lanka

◯ Sudan
◯ Suriname
◯ Svalbard and Jan Mayen
◯ Swaziland
◯ Sweden
◯ Switzerland
◯ Syria
◯ Taiwan
◯ Tajikistan
◯ Tanzania
◯ Thailand
◯ Timor-Leste
◯ Togo
◯ Tokelau
◯ Tonga
◯ Trinidad and Tobago
◯ Tunisia
◯ Turkey
◯ Turkmenistan
◯ Turks and Caicos Islands
◯ Tuvalu
◯ Uganda
◯ Ukraine
◯ United Arab Emirates
◯ United Kingdom
◯ United States of America
◯ United States Minor Outlying Islands
◯ Uruguay
◯ Uzbekistan
◯ Vanuatu
◯ Venezuela
◯ Vietnam
◯ Virgin Islands (British)
◯ Virgin Islands (U.S.)
◯ Wallis and Futuna
◯ Yemen
◯ Zambia
◯ Zimbabwe
◯ Other (specify)
(Start typing to search)

Specify country of birth

____

Country of main residence

◯ Unknown
◯ Afghanistan
◯ Åland Islands
◯ Albania
◯ Algeria
◯ American Samoa
◯ Andorra
◯ Angola
◯ Anguilla
◯ Antarctica
◯ Antigua and Barbuda
◯ Argentina
◯ Armenia
◯ Aruba
◯ Australia
◯ Austria
◯ Azerbaijan
◯ Bahamas
◯ Bahrain
◯ Bangladesh
◯ Barbados
◯ Belarus
◯ Belgium
◯ Belize
◯ Benin
◯ Bermuda
◯ Bhutan
◯ Bolivia
◯ Bonaire, Sint Eustatius and Saba
◯ Bosnia and Herzegovina
◯ Botswana
◯ Bouvet Island
◯ Brazil
◯ British Indian Ocean Territory
◯ Brunei Darussalam
◯ Bulgaria
◯ Burkina Faso
◯ Burundi
◯ Cabo Verde
◯ Cambodia
◯ Cameroon
◯ Canada
◯ Cayman Islands
◯ Central African Republic
◯ Chad
◯ Chile
◯ China
◯ Christmas Island
◯ Cocos (Keeling) Islands
◯ Colombia
◯ Comoros
◯ Congo-Brazzaville
◯ Congo (DRC)
◯ Cook Islands
◯ Costa Rica
◯ Côte d'Ivoire
◯ Croatia
◯ Cuba
◯ Curaçao
◯ Cyprus
◯ Czechia
◯ Denmark
◯ Djibouti
◯ Dominica
◯ Dominican Republic
◯ Ecuador
◯ Egypt
◯ El Salvador
◯ Equatorial Guinea

◯ Eritrea
◯ Estonia
◯ Ethiopia
◯ Falkland Islands
◯ Faroe Islands
◯ Fiji
◯ Finland
◯ France
◯ French Guiana
◯ French Polynesia
◯ French Southern Territories
◯ Gabon
◯ Gambia
◯ Georgia
◯ Germany
◯ Ghana
◯ Gibraltar
◯ Greece
◯ Greenland
◯ Grenada
◯ Guadeloupe
◯ Guam
◯ Guatemala
◯ Guernsey
◯ Guinea
◯ Guinea-Bissau
◯ Guyana
◯ Haiti
◯ Heard Island and McDonald Islands
◯ Holy See
◯ Honduras
◯ Hong Kong
◯ Hungary
◯ Iceland
◯ India
◯ Indonesia
◯ Iran
◯ Iraq
◯ Ireland
◯ Isle of Man
◯ Israel
◯ Italy
◯ Jamaica
◯ Japan
◯ Jersey
◯ Jordan
◯ Kazakhstan
◯ Kenya
◯ Kiribati
◯ Kuwait
◯ Kyrgyzstan
◯ Laos
◯ Latvia
◯ Lebanon
◯ Lesotho
◯ Liberia
◯ Libya
◯ Liechtenstein
◯ Lithuania
◯ Luxembourg
◯ Macao
◯ Macedonia
◯ Madagascar
◯ Malawi
◯ Malaysia
◯ Maldives
◯ Mali
◯ Malta
◯ Marshall Islands
◯ Martinique
◯ Mauritania

◯ Mauritius
◯ Mayotte
◯ Mexico
◯ Micronesia
◯ Moldova
◯ Monaco
◯ Mongolia
◯ Montenegro
◯ Montserrat
◯ Morocco
◯ Mozambique
◯ Myanmar
◯ Namibia
◯ Nauru
◯ Nepal
◯ Netherlands
◯ New Caledonia
◯ New Zealand
◯ Nicaragua
◯ Niger
◯ Nigeria
◯ Niue
◯ Norfolk Island
◯ North Korea
◯ Northern Mariana Islands
◯ Norway
◯ Oman
◯ Pakistan
◯ Palau
◯ Palestine
◯ Panama
◯ Papua New Guinea
◯ Paraguay
◯ Peru
◯ Philippines
◯ Pitcairn
◯ Poland
◯ Portugal
◯ Puerto Rico
◯ Qatar
◯ Réunion
◯ Romania
◯ Russian Federation
◯ Rwanda
◯ Saint Barthélemy
◯ Saint Helena, Ascension and Tristan da Cunha
◯ Saint Kitts and Nevis
◯ Saint Lucia
◯ Saint Martin (French part)
◯ Saint Pierre and Miquelon
◯ Saint Vincent and the Grenadines
◯ Samoa
◯ San Marino
◯ Sao Tome and Principe
◯ Saudi Arabia
◯ Senegal
◯ Serbia
◯ Seychelles
◯ Sierra Leone
◯ Singapore
◯ Sint Maarten (Dutch part)
◯ Slovakia
◯ Slovenia
◯ Solomon Islands
◯ Somalia
◯ South Africa
◯ South Georgia and the South Sandwich Islands
◯ South Korea
◯ South Sudan
◯ Spain
◯ Sri Lanka

◯ Sudan
◯ Suriname
◯ Svalbard and Jan Mayen
◯ Swaziland
◯ Sweden
◯ Switzerland
◯ Syria
◯ Taiwan
◯ Tajikistan
◯ Tanzania
◯ Thailand
◯ Timor-Leste
◯ Togo
◯ Tokelau
◯ Tonga
◯ Trinidad and Tobago
◯ Tunisia
◯ Turkey
◯ Turkmenistan
◯ Turks and Caicos Islands
◯ Tuvalu
◯ Uganda
◯ Ukraine
◯ United Arab Emirates
◯ United Kingdom
◯ United States of America
◯ United States Minor Outlying Islands
◯ Uruguay
◯ Uzbekistan
◯ Vanuatu
◯ Venezuela
◯ Vietnam
◯ Virgin Islands (British)
◯ Virgin Islands (U.S.)
◯ Wallis and Futuna
◯ Yemen
◯ Zambia
◯ Zimbabwe
◯ Other (specify)
(Start typing to search)

Specify country of residence

____

Source file code

____
(Please specify the internal patient file code at your institution.)

Exposure Information

Exposure context

Patient exposure context	☐ Short-term traveler (< 6 months)<br>☐ Long-term traveler / expatriate (> 6 months)<br>☐ Migrant<br>☐ Autochthonous case
Which was the reason for traveling?	☐ Tourism / Leisure<br>☐ Visiting friends or relatives (VFR)<br>☐ Business or professional trip<br>☐ Humanitarian mission / Volunteer work<br>☐ Religious pilgrimage<br>☐ Other
Which was the reason for traveling?	☐ Humanitarian mission / volunteer work<br>☐ Professional / academic assignment<br>☐ Visiting friends or relatives (VFR)<br>☐ Long-term tourism / travel<br>☐ Retirement migration<br>☐ Other (specify)
Please specify other reason for traveling	__________________________________
Date of departure	__________________________________<br>(If unsure: use 01-MM-YYYY if month known, or 01-01-YYYY if only year known.)
Date of return	__________________________________<br>(If unsure: use 01-MM-YYYY if month known, or 01-01-YYYY if only year known.)
Date of arrival to current country	__________________________________<br>(If unsure: use 01-MM-YYYY if month known, or 01-01-YYYY if only year known.)

Country of (most likely) exposure

◯ Unknown
◯ Afghanistan
◯ Åland Islands
◯ Albania
◯ Algeria
◯ American Samoa
◯ Andorra
◯ Angola
◯ Anguilla
◯ Antarctica
◯ Antigua and Barbuda
◯ Argentina
◯ Armenia
◯ Aruba
◯ Australia
◯ Austria
◯ Azerbaijan
◯ Bahamas
◯ Bahrain
◯ Bangladesh
◯ Barbados
◯ Belarus
◯ Belgium
◯ Belize
◯ Benin
◯ Bermuda
◯ Bhutan
◯ Bolivia
◯ Bonaire, Sint Eustatius and Saba
◯ Bosnia and Herzegovina
◯ Botswana
◯ Bouvet Island
◯ Brazil
◯ British Indian Ocean Territory
◯ Brunei Darussalam
◯ Bulgaria
◯ Burkina Faso
◯ Burundi
◯ Cabo Verde
◯ Cambodia
◯ Cameroon
◯ Canada
◯ Cayman Islands
◯ Central African Republic
◯ Chad
◯ Chile
◯ China
◯ Christmas Island
◯ Cocos (Keeling) Islands
◯ Colombia
◯ Comoros
◯ Congo-Brazzaville
◯ Congo (DRC)
◯ Cook Islands
◯ Costa Rica
◯ Côte d'Ivoire
◯ Croatia
◯ Cuba
◯ Curaçao
◯ Cyprus
◯ Czechia
◯ Denmark
◯ Djibouti
◯ Dominica
◯ Dominican Republic
◯ Ecuador
◯ Egypt
◯ El Salvador
◯ Equatorial Guinea

◯ Eritrea
◯ Estonia
◯ Ethiopia
◯ Falkland Islands
◯ Faroe Islands
◯ Fiji
◯ Finland
◯ France
◯ French Guiana
◯ French Polynesia
◯ French Southern Territories
◯ Gabon
◯ Gambia
◯ Georgia
◯ Germany
◯ Ghana
◯ Gibraltar
◯ Greece
◯ Greenland
◯ Grenada
◯ Guadeloupe
◯ Guam
◯ Guatemala
◯ Guernsey
◯ Guinea
◯ Guinea-Bissau
◯ Guyana
◯ Haiti
◯ Heard Island and McDonald Islands
◯ Holy See
◯ Honduras
◯ Hong Kong
◯ Hungary
◯ Iceland
◯ India
◯ Indonesia
◯ Iran
◯ Iraq
◯ Ireland
◯ Isle of Man
◯ Israel
◯ Italy
◯ Jamaica
◯ Japan
◯ Jersey
◯ Jordan
◯ Kazakhstan
◯ Kenya
◯ Kiribati
◯ Kuwait
◯ Kyrgyzstan
◯ Laos
◯ Latvia
◯ Lebanon
◯ Lesotho
◯ Liberia
◯ Libya
◯ Liechtenstein
◯ Lithuania
◯ Luxembourg
◯ Macao
◯ Macedonia
◯ Madagascar
◯ Malawi
◯ Malaysia
◯ Maldives
◯ Mali
◯ Malta
◯ Marshall Islands
◯ Martinique
◯ Mauritania

◯ Mauritius
◯ Mayotte
◯ Mexico
◯ Micronesia
◯ Moldova
◯ Monaco
◯ Mongolia
◯ Montenegro
◯ Montserrat
◯ Morocco
◯ Mozambique
◯ Myanmar
◯ Namibia
◯ Nauru
◯ Nepal
◯ Netherlands
◯ New Caledonia
◯ New Zealand
◯ Nicaragua
◯ Niger
◯ Nigeria
◯ Niue
◯ Norfolk Island
◯ North Korea
◯ Northern Mariana Islands
◯ Norway
◯ Oman
◯ Pakistan
◯ Palau
◯ Palestine
◯ Panama
◯ Papua New Guinea
◯ Paraguay
◯ Peru
◯ Philippines
◯ Pitcairn
◯ Poland
◯ Portugal
◯ Puerto Rico
◯ Qatar
◯ Réunion
◯ Romania
◯ Russian Federation
◯ Rwanda
◯ Saint Barthélemy
◯ Saint Helena, Ascension and Tristan da Cunha
◯ Saint Kitts and Nevis
◯ Saint Lucia
◯ Saint Martin (French part)
◯ Saint Pierre and Miquelon
◯ Saint Vincent and the Grenadines
◯ Samoa
◯ San Marino
◯ Sao Tome and Principe
◯ Saudi Arabia
◯ Senegal
◯ Serbia
◯ Seychelles
◯ Sierra Leone
◯ Singapore
◯ Sint Maarten (Dutch part)
◯ Slovakia
◯ Slovenia
◯ Solomon Islands
◯ Somalia
◯ South Africa
◯ South Georgia and the South Sandwich Islands
◯ South Korea
◯ South Sudan
◯ Spain
◯ Sri Lanka

- ◯ Sudan
- ◯ Suriname
- ◯ Svalbard and Jan Mayen
- ◯ Swaziland
- ◯ Sweden
- ◯ Switzerland
- ◯ Syria
- ◯ Taiwan
- ◯ Tajikistan
- ◯ Tanzania
- ◯ Thailand
- ◯ Timor-Leste
- ◯ Togo
- ◯ Tokelau
- ◯ Tonga
- ◯ Trinidad and Tobago
- ◯ Tunisia
- ◯ Turkey
- ◯ Turkmenistan
- ◯ Turks and Caicos Islands
- ◯ Tuvalu
- ◯ Uganda
- ◯ Ukraine
- ◯ United Arab Emirates
- ◯ United Kingdom
- ◯ United States of America
- ◯ United States Minor Outlying Islands
- ◯ Uruguay
- ◯ Uzbekistan
- ◯ Vanuatu
- ◯ Venezuela
- ◯ Vietnam
- ◯ Virgin Islands (British)
- ◯ Virgin Islands (U.S.)
- ◯ Wallis and Futuna
- ◯ Yemen
- ◯ Zambia
- ◯ Zimbabwe
- ◯ Other (specify)

(Start typing to search)

Specify country of likely exposure

____

Other possible country of exposure

◯ Not applicable
◯ Unknown
◯ Afghanistan
◯ Åland Islands
◯ Albania
◯ Algeria
◯ American Samoa
◯ Andorra
◯ Angola
◯ Anguilla
◯ Antarctica
◯ Antigua and Barbuda
◯ Argentina
◯ Armenia
◯ Aruba
◯ Australia
◯ Austria
◯ Azerbaijan
◯ Bahamas
◯ Bahrain
◯ Bangladesh
◯ Barbados
◯ Belarus
◯ Belgium
◯ Belize
◯ Benin
◯ Bermuda
◯ Bhutan
◯ Bolivia
◯ Bonaire, Sint Eustatius and Saba
◯ Bosnia and Herzegovina
◯ Botswana
◯ Bouvet Island
◯ Brazil
◯ British Indian Ocean Territory
◯ Brunei Darussalam
◯ Bulgaria
◯ Burkina Faso
◯ Burundi
◯ Cabo Verde
◯ Cambodia
◯ Cameroon
◯ Canada
◯ Cayman Islands
◯ Central African Republic
◯ Chad
◯ Chile
◯ China
◯ Christmas Island
◯ Cocos (Keeling) Islands
◯ Colombia
◯ Comoros
◯ Congo-Brazzaville
◯ Congo (DRC)
◯ Cook Islands
◯ Costa Rica
◯ Côte d'Ivoire
◯ Croatia
◯ Cuba
◯ Curaçao
◯ Cyprus
◯ Czechia
◯ Denmark
◯ Djibouti
◯ Dominica
◯ Dominican Republic
◯ Ecuador
◯ Egypt
◯ El Salvador

◯ Equatorial Guinea
◯ Eritrea
◯ Estonia
◯ Ethiopia
◯ Falkland Islands
◯ Faroe Islands
◯ Fiji
◯ Finland
◯ France
◯ French Guiana
◯ French Polynesia
◯ French Southern Territories
◯ Gabon
◯ Gambia
◯ Georgia
◯ Germany
◯ Ghana
◯ Gibraltar
◯ Greece
◯ Greenland
◯ Grenada
◯ Guadeloupe
◯ Guam
◯ Guatemala
◯ Guernsey
◯ Guinea
◯ Guinea-Bissau
◯ Guyana
◯ Haiti
◯ Heard Island and McDonald Islands
◯ Holy See
◯ Honduras
◯ Hong Kong
◯ Hungary
◯ Iceland
◯ India
◯ Indonesia
◯ Iran
◯ Iraq
◯ Ireland
◯ Isle of Man
◯ Israel
◯ Italy
◯ Jamaica
◯ Japan
◯ Jersey
◯ Jordan
◯ Kazakhstan
◯ Kenya
◯ Kiribati
◯ Kuwait
◯ Kyrgyzstan
◯ Laos
◯ Latvia
◯ Lebanon
◯ Lesotho
◯ Liberia
◯ Libya
◯ Liechtenstein
◯ Lithuania
◯ Luxembourg
◯ Macao
◯ Macedonia
◯ Madagascar
◯ Malawi
◯ Malaysia
◯ Maldives
◯ Mali
◯ Malta
◯ Marshall Islands
◯ Martinique

◯ Mauritania
◯ Mauritius
◯ Mayotte
◯ Mexico
◯ Micronesia
◯ Moldova
◯ Monaco
◯ Mongolia
◯ Montenegro
◯ Montserrat
◯ Morocco
◯ Mozambique
◯ Myanmar
◯ Namibia
◯ Nauru
◯ Nepal
◯ Netherlands
◯ New Caledonia
◯ New Zealand
◯ Nicaragua
◯ Niger
◯ Nigeria
◯ Niue
◯ Norfolk Island
◯ North Korea
◯ Northern Mariana Islands
◯ Norway
◯ Oman
◯ Pakistan
◯ Palau
◯ Palestine
◯ Panama
◯ Papua New Guinea
◯ Paraguay
◯ Peru
◯ Philippines
◯ Pitcairn
◯ Poland
◯ Portugal
◯ Puerto Rico
◯ Qatar
◯ Réunion
◯ Romania
◯ Russian Federation
◯ Rwanda
◯ Saint Barthélemy
◯ Saint Helena, Ascension and Tristan da Cunha
◯ Saint Kitts and Nevis
◯ Saint Lucia
◯ Saint Martin (French part)
◯ Saint Pierre and Miquelon
◯ Saint Vincent and the Grenadines
◯ Samoa
◯ San Marino
◯ Sao Tome and Principe
◯ Saudi Arabia
◯ Senegal
◯ Serbia
◯ Seychelles
◯ Sierra Leone
◯ Singapore
◯ Sint Maarten (Dutch part)
◯ Slovakia
◯ Slovenia
◯ Solomon Islands
◯ Somalia
◯ South Africa
◯ South Georgia and the South Sandwich Islands
◯ South Korea
◯ South Sudan
◯ Spain

◯ Sri Lanka
◯ Sudan
◯ Suriname
◯ Svalbard and Jan Mayen
◯ Swaziland
◯ Sweden
◯ Switzerland
◯ Syria
◯ Taiwan
◯ Tajikistan
◯ Tanzania
◯ Thailand
◯ Timor-Leste
◯ Togo
◯ Tokelau
◯ Tonga
◯ Trinidad and Tobago
◯ Tunisia
◯ Turkey
◯ Turkmenistan
◯ Turks and Caicos Islands
◯ Tuvalu
◯ Uganda
◯ Ukraine
◯ United Arab Emirates
◯ United Kingdom
◯ United States of America
◯ United States Minor Outlying Islands
◯ Uruguay
◯ Uzbekistan
◯ Vanuatu
◯ Venezuela
◯ Vietnam
◯ Virgin Islands (British)
◯ Virgin Islands (U.S.)
◯ Wallis and Futuna
◯ Yemen
◯ Zambia
◯ Zimbabwe
◯ Other
(Start typing to search)

Specify country of likely exposure

____

2nd other possible country of exposure

- ◯ Not applicable
- ◯ Unknown
- ◯ Afghanistan
- ◯ Åland Islands
- ◯ Albania
- ◯ Algeria
- ◯ American Samoa
- ◯ Andorra
- ◯ Angola
- ◯ Anguilla
- ◯ Antarctica
- ◯ Antigua and Barbuda
- ◯ Argentina
- ◯ Armenia
- ◯ Aruba
- ◯ Australia
- ◯ Austria
- ◯ Azerbaijan
- ◯ Bahamas
- ◯ Bahrain
- ◯ Bangladesh
- ◯ Barbados
- ◯ Belarus
- ◯ Belgium
- ◯ Belize
- ◯ Benin
- ◯ Bermuda
- ◯ Bhutan
- ◯ Bolivia
- ◯ Bonaire, Sint Eustatius and Saba
- ◯ Bosnia and Herzegovina
- ◯ Botswana
- ◯ Bouvet Island
- ◯ Brazil
- ◯ British Indian Ocean Territory
- ◯ Brunei Darussalam
- ◯ Bulgaria
- ◯ Burkina Faso
- ◯ Burundi
- ◯ Cabo Verde
- ◯ Cambodia
- ◯ Cameroon
- ◯ Canada
- ◯ Cayman Islands
- ◯ Central African Republic
- ◯ Chad
- ◯ Chile
- ◯ China
- ◯ Christmas Island
- ◯ Cocos (Keeling) Islands
- ◯ Colombia
- ◯ Comoros
- ◯ Congo-Brazzaville
- ◯ Congo (DRC)
- ◯ Cook Islands
- ◯ Costa Rica
- ◯ Côte d'Ivoire
- ◯ Croatia
- ◯ Cuba
- ◯ Curaçao
- ◯ Cyprus
- ◯ Czechia
- ◯ Denmark
- ◯ Djibouti
- ◯ Dominica
- ◯ Dominican Republic
- ◯ Ecuador
- ◯ Egypt
- ◯ El Salvador

◯ Equatorial Guinea
◯ Eritrea
◯ Estonia
◯ Ethiopia
◯ Falkland Islands
◯ Faroe Islands
◯ Fiji
◯ Finland
◯ France
◯ French Guiana
◯ French Polynesia
◯ French Southern Territories
◯ Gabon
◯ Gambia
◯ Georgia
◯ Germany
◯ Ghana
◯ Gibraltar
◯ Greece
◯ Greenland
◯ Grenada
◯ Guadeloupe
◯ Guam
◯ Guatemala
◯ Guernsey
◯ Guinea
◯ Guinea-Bissau
◯ Guyana
◯ Haiti
◯ Heard Island and McDonald Islands
◯ Holy See
◯ Honduras
◯ Hong Kong
◯ Hungary
◯ Iceland
◯ India
◯ Indonesia
◯ Iran
◯ Iraq
◯ Ireland
◯ Isle of Man
◯ Israel
◯ Italy
◯ Jamaica
◯ Japan
◯ Jersey
◯ Jordan
◯ Kazakhstan
◯ Kenya
◯ Kiribati
◯ Kuwait
◯ Kyrgyzstan
◯ Laos
◯ Latvia
◯ Lebanon
◯ Lesotho
◯ Liberia
◯ Libya
◯ Liechtenstein
◯ Lithuania
◯ Luxembourg
◯ Macao
◯ Macedonia
◯ Madagascar
◯ Malawi
◯ Malaysia
◯ Maldives
◯ Mali
◯ Malta
◯ Marshall Islands
◯ Martinique

◯ Mauritania
◯ Mauritius
◯ Mayotte
◯ Mexico
◯ Micronesia
◯ Moldova
◯ Monaco
◯ Mongolia
◯ Montenegro
◯ Montserrat
◯ Morocco
◯ Mozambique
◯ Myanmar
◯ Namibia
◯ Nauru
◯ Nepal
◯ Netherlands
◯ New Caledonia
◯ New Zealand
◯ Nicaragua
◯ Niger
◯ Nigeria
◯ Niue
◯ Norfolk Island
◯ North Korea
◯ Northern Mariana Islands
◯ Norway
◯ Oman
◯ Pakistan
◯ Palau
◯ Palestine
◯ Panama
◯ Papua New Guinea
◯ Paraguay
◯ Peru
◯ Philippines
◯ Pitcairn
◯ Poland
◯ Portugal
◯ Puerto Rico
◯ Qatar
◯ Réunion
◯ Romania
◯ Russian Federation
◯ Rwanda
◯ Saint Barthélemy
◯ Saint Helena, Ascension and Tristan da Cunha
◯ Saint Kitts and Nevis
◯ Saint Lucia
◯ Saint Martin (French part)
◯ Saint Pierre and Miquelon
◯ Saint Vincent and the Grenadines
◯ Samoa
◯ San Marino
◯ Sao Tome and Principe
◯ Saudi Arabia
◯ Senegal
◯ Serbia
◯ Seychelles
◯ Sierra Leone
◯ Singapore
◯ Sint Maarten (Dutch part)
◯ Slovakia
◯ Slovenia
◯ Solomon Islands
◯ Somalia
◯ South Africa
◯ South Georgia and the South Sandwich Islands
◯ South Korea
◯ South Sudan
◯ Spain

◯ Sri Lanka
◯ Sudan
◯ Suriname
◯ Svalbard and Jan Mayen
◯ Swaziland
◯ Sweden
◯ Switzerland
◯ Syria
◯ Taiwan
◯ Tajikistan
◯ Tanzania
◯ Thailand
◯ Timor-Leste
◯ Togo
◯ Tokelau
◯ Tonga
◯ Trinidad and Tobago
◯ Tunisia
◯ Turkey
◯ Turkmenistan
◯ Turks and Caicos Islands
◯ Tuvalu
◯ Uganda
◯ Ukraine
◯ United Arab Emirates
◯ United Kingdom
◯ United States of America
◯ United States Minor Outlying Islands
◯ Uruguay
◯ Uzbekistan
◯ Vanuatu
◯ Venezuela
◯ Vietnam
◯ Virgin Islands (British)
◯ Virgin Islands (U.S.)
◯ Wallis and Futuna
◯ Yemen
◯ Zambia
◯ Zimbabwe
◯ Other
(Start typing to search)

Specify country of likely exposure

____

3rd other possible country of exposure

◯ Not applicable
◯ Unknown
◯ Afghanistan
◯ Åland Islands
◯ Albania
◯ Algeria
◯ American Samoa
◯ Andorra
◯ Angola
◯ Anguilla
◯ Antarctica
◯ Antigua and Barbuda
◯ Argentina
◯ Armenia
◯ Aruba
◯ Australia
◯ Austria
◯ Azerbaijan
◯ Bahamas
◯ Bahrain
◯ Bangladesh
◯ Barbados
◯ Belarus
◯ Belgium
◯ Belize
◯ Benin
◯ Bermuda
◯ Bhutan
◯ Bolivia
◯ Bonaire, Sint Eustatius and Saba
◯ Bosnia and Herzegovina
◯ Botswana
◯ Bouvet Island
◯ Brazil
◯ British Indian Ocean Territory
◯ Brunei Darussalam
◯ Bulgaria
◯ Burkina Faso
◯ Burundi
◯ Cabo Verde
◯ Cambodia
◯ Cameroon
◯ Canada
◯ Cayman Islands
◯ Central African Republic
◯ Chad
◯ Chile
◯ China
◯ Christmas Island
◯ Cocos (Keeling) Islands
◯ Colombia
◯ Comoros
◯ Congo-Brazzaville
◯ Congo (DRC)
◯ Cook Islands
◯ Costa Rica
◯ Côte d'Ivoire
◯ Croatia
◯ Cuba
◯ Curaçao
◯ Cyprus
◯ Czechia
◯ Denmark
◯ Djibouti
◯ Dominica
◯ Dominican Republic
◯ Ecuador
◯ Egypt
◯ El Salvador

◯ Equatorial Guinea
◯ Eritrea
◯ Estonia
◯ Ethiopia
◯ Falkland Islands
◯ Faroe Islands
◯ Fiji
◯ Finland
◯ France
◯ French Guiana
◯ French Polynesia
◯ French Southern Territories
◯ Gabon
◯ Gambia
◯ Georgia
◯ Germany
◯ Ghana
◯ Gibraltar
◯ Greece
◯ Greenland
◯ Grenada
◯ Guadeloupe
◯ Guam
◯ Guatemala
◯ Guernsey
◯ Guinea
◯ Guinea-Bissau
◯ Guyana
◯ Haiti
◯ Heard Island and McDonald Islands
◯ Holy See
◯ Honduras
◯ Hong Kong
◯ Hungary
◯ Iceland
◯ India
◯ Indonesia
◯ Iran
◯ Iraq
◯ Ireland
◯ Isle of Man
◯ Israel
◯ Italy
◯ Jamaica
◯ Japan
◯ Jersey
◯ Jordan
◯ Kazakhstan
◯ Kenya
◯ Kiribati
◯ Kuwait
◯ Kyrgyzstan
◯ Laos
◯ Latvia
◯ Lebanon
◯ Lesotho
◯ Liberia
◯ Libya
◯ Liechtenstein
◯ Lithuania
◯ Luxembourg
◯ Macao
◯ Macedonia
◯ Madagascar
◯ Malawi
◯ Malaysia
◯ Maldives
◯ Mali
◯ Malta
◯ Marshall Islands
◯ Martinique

◯ Mauritania
◯ Mauritius
◯ Mayotte
◯ Mexico
◯ Micronesia
◯ Moldova
◯ Monaco
◯ Mongolia
◯ Montenegro
◯ Montserrat
◯ Morocco
◯ Mozambique
◯ Myanmar
◯ Namibia
◯ Nauru
◯ Nepal
◯ Netherlands
◯ New Caledonia
◯ New Zealand
◯ Nicaragua
◯ Niger
◯ Nigeria
◯ Niue
◯ Norfolk Island
◯ North Korea
◯ Northern Mariana Islands
◯ Norway
◯ Oman
◯ Pakistan
◯ Palau
◯ Palestine
◯ Panama
◯ Papua New Guinea
◯ Paraguay
◯ Peru
◯ Philippines
◯ Pitcairn
◯ Poland
◯ Portugal
◯ Puerto Rico
◯ Qatar
◯ Réunion
◯ Romania
◯ Russian Federation
◯ Rwanda
◯ Saint Barthélemy
◯ Saint Helena, Ascension and Tristan da Cunha
◯ Saint Kitts and Nevis
◯ Saint Lucia
◯ Saint Martin (French part)
◯ Saint Pierre and Miquelon
◯ Saint Vincent and the Grenadines
◯ Samoa
◯ San Marino
◯ Sao Tome and Principe
◯ Saudi Arabia
◯ Senegal
◯ Serbia
◯ Seychelles
◯ Sierra Leone
◯ Singapore
◯ Sint Maarten (Dutch part)
◯ Slovakia
◯ Slovenia
◯ Solomon Islands
◯ Somalia
◯ South Africa
◯ South Georgia and the South Sandwich Islands
◯ South Korea
◯ South Sudan
◯ Spain

◯ Sri Lanka
◯ Sudan
◯ Suriname
◯ Svalbard and Jan Mayen
◯ Swaziland
◯ Sweden
◯ Switzerland
◯ Syria
◯ Taiwan
◯ Tajikistan
◯ Tanzania
◯ Thailand
◯ Timor-Leste
◯ Togo
◯ Tokelau
◯ Tonga
◯ Trinidad and Tobago
◯ Tunisia
◯ Turkey
◯ Turkmenistan
◯ Turks and Caicos Islands
◯ Tuvalu
◯ Uganda
◯ Ukraine
◯ United Arab Emirates
◯ United Kingdom
◯ United States of America
◯ United States Minor Outlying Islands
◯ Uruguay
◯ Uzbekistan
◯ Vanuatu
◯ Venezuela
◯ Vietnam
◯ Virgin Islands (British)
◯ Virgin Islands (U.S.)
◯ Wallis and Futuna
◯ Yemen
◯ Zambia
◯ Zimbabwe
◯ Other
(Start typing to search)

Specify country of likely exposure

____

4th other possible country of exposure

◯ Not applicable
◯ Unknown
◯ Afghanistan
◯ Åland Islands
◯ Albania
◯ Algeria
◯ American Samoa
◯ Andorra
◯ Angola
◯ Anguilla
◯ Antarctica
◯ Antigua and Barbuda
◯ Argentina
◯ Armenia
◯ Aruba
◯ Australia
◯ Austria
◯ Azerbaijan
◯ Bahamas
◯ Bahrain
◯ Bangladesh
◯ Barbados
◯ Belarus
◯ Belgium
◯ Belize
◯ Benin
◯ Bermuda
◯ Bhutan
◯ Bolivia
◯ Bonaire, Sint Eustatius and Saba
◯ Bosnia and Herzegovina
◯ Botswana
◯ Bouvet Island
◯ Brazil
◯ British Indian Ocean Territory
◯ Brunei Darussalam
◯ Bulgaria
◯ Burkina Faso
◯ Burundi
◯ Cabo Verde
◯ Cambodia
◯ Cameroon
◯ Canada
◯ Cayman Islands
◯ Central African Republic
◯ Chad
◯ Chile
◯ China
◯ Christmas Island
◯ Cocos (Keeling) Islands
◯ Colombia
◯ Comoros
◯ Congo-Brazzaville
◯ Congo (DRC)
◯ Cook Islands
◯ Costa Rica
◯ Côte d'Ivoire
◯ Croatia
◯ Cuba
◯ Curaçao
◯ Cyprus
◯ Czechia
◯ Denmark
◯ Djibouti
◯ Dominica
◯ Dominican Republic
◯ Ecuador
◯ Egypt
◯ El Salvador

◯ Equatorial Guinea
◯ Eritrea
◯ Estonia
◯ Ethiopia
◯ Falkland Islands
◯ Faroe Islands
◯ Fiji
◯ Finland
◯ France
◯ French Guiana
◯ French Polynesia
◯ French Southern Territories
◯ Gabon
◯ Gambia
◯ Georgia
◯ Germany
◯ Ghana
◯ Gibraltar
◯ Greece
◯ Greenland
◯ Grenada
◯ Guadeloupe
◯ Guam
◯ Guatemala
◯ Guernsey
◯ Guinea
◯ Guinea-Bissau
◯ Guyana
◯ Haiti
◯ Heard Island and McDonald Islands
◯ Holy See
◯ Honduras
◯ Hong Kong
◯ Hungary
◯ Iceland
◯ India
◯ Indonesia
◯ Iran
◯ Iraq
◯ Ireland
◯ Isle of Man
◯ Israel
◯ Italy
◯ Jamaica
◯ Japan
◯ Jersey
◯ Jordan
◯ Kazakhstan
◯ Kenya
◯ Kiribati
◯ Kuwait
◯ Kyrgyzstan
◯ Laos
◯ Latvia
◯ Lebanon
◯ Lesotho
◯ Liberia
◯ Libya
◯ Liechtenstein
◯ Lithuania
◯ Luxembourg
◯ Macao
◯ Macedonia
◯ Madagascar
◯ Malawi
◯ Malaysia
◯ Maldives
◯ Mali
◯ Malta
◯ Marshall Islands
◯ Martinique

◯ Mauritania
◯ Mauritius
◯ Mayotte
◯ Mexico
◯ Micronesia
◯ Moldova
◯ Monaco
◯ Mongolia
◯ Montenegro
◯ Montserrat
◯ Morocco
◯ Mozambique
◯ Myanmar
◯ Namibia
◯ Nauru
◯ Nepal
◯ Netherlands
◯ New Caledonia
◯ New Zealand
◯ Nicaragua
◯ Niger
◯ Nigeria
◯ Niue
◯ Norfolk Island
◯ North Korea
◯ Northern Mariana Islands
◯ Norway
◯ Oman
◯ Pakistan
◯ Palau
◯ Palestine
◯ Panama
◯ Papua New Guinea
◯ Paraguay
◯ Peru
◯ Philippines
◯ Pitcairn
◯ Poland
◯ Portugal
◯ Puerto Rico
◯ Qatar
◯ Réunion
◯ Romania
◯ Russian Federation
◯ Rwanda
◯ Saint Barthélemy
◯ Saint Helena, Ascension and Tristan da Cunha
◯ Saint Kitts and Nevis
◯ Saint Lucia
◯ Saint Martin (French part)
◯ Saint Pierre and Miquelon
◯ Saint Vincent and the Grenadines
◯ Samoa
◯ San Marino
◯ Sao Tome and Principe
◯ Saudi Arabia
◯ Senegal
◯ Serbia
◯ Seychelles
◯ Sierra Leone
◯ Singapore
◯ Sint Maarten (Dutch part)
◯ Slovakia
◯ Slovenia
◯ Solomon Islands
◯ Somalia
◯ South Africa
◯ South Georgia and the South Sandwich Islands
◯ South Korea
◯ South Sudan
◯ Spain

◯ Sri Lanka
◯ Sudan
◯ Suriname
◯ Svalbard and Jan Mayen
◯ Swaziland
◯ Sweden
◯ Switzerland
◯ Syria
◯ Taiwan
◯ Tajikistan
◯ Tanzania
◯ Thailand
◯ Timor-Leste
◯ Togo
◯ Tokelau
◯ Tonga
◯ Trinidad and Tobago
◯ Tunisia
◯ Turkey
◯ Turkmenistan
◯ Turks and Caicos Islands
◯ Tuvalu
◯ Uganda
◯ Ukraine
◯ United Arab Emirates
◯ United Kingdom
◯ United States of America
◯ United States Minor Outlying Islands
◯ Uruguay
◯ Uzbekistan
◯ Vanuatu
◯ Venezuela
◯ Vietnam
◯ Virgin Islands (British)
◯ Virgin Islands (U.S.)
◯ Wallis and Futuna
◯ Yemen
◯ Zambia
◯ Zimbabwe
◯ Other
(Start typing to search)

Specify country of likely exposure

____

Approximate date of suspected exposure

____
(If unsure: use 01-MM-YYYY if month known, or 01-01-YYYY if only year known.)

Animal contact

Has the patient had regular contact with animals during the stay in the endemic area?

◯ Yes
◯ No
◯ Not assessed

What kind of animal(s)?	☐ Dog(s)<br>☐ Cat(s)<br>☐ Rodent(s) (e.g., rats, mice, squirrels)<br>☐ Livestock (e.g., goats, sheep, cattle, pigs)<br>☐ Poultry (e.g., chickens, ducks)<br>☐ Wild animals (e.g., hunting, handling bush meat)<br>☐ Other (please specify below)<br>☐ Unknown
Please specify which kind of animal(s)	__________________________________
Additional comments in this section (exposure-related information)	____

Clinical features at diagnosis

Date of first visit

____

Clinical characterization

Type of Leishmaniasis

- ◯ Cutaneous leishmaniasis (CL): only skin involved
- ◯ Mucocutaneous leishmaniasis (MCL): both mucosa and skin involved
- ◯ Mucosal leishmaniasis (ML): only mucosa involved
- ◯ Post-Kala-azar dermal Leishmaniasis (PKDL)
- ◯ Other (specify)
- ◯ Unknown

Specify type of Leishmaniasis

____

Type of episode

- ◯ New
- ◯ Relapse
- ◯ Unknown

(Select "Relapse" only if there was complete cure before, followed by recurrence at the same site or new lesions elsewhere caused by the same parasite (ideally confirmed by species typing).)

Episode number

____

(Specify the number of this episode starting from the appearance of first lesion.)

Date of diagnosis of previous episode

____

Type of Leishmaniasis in previous episode

- ◯ Cutaneous leishmaniasis (CL)
- ◯ Mucocutaneous leishmaniasis (MCL)
- ◯ Mucosal leishmaniasis (ML)
- ◯ Visceral leishmaniasis (VL)
- ◯ Unknown

Do you know which treatment(s) did the patient receive for previous infection?	☐ Liposomal Amphotericin B (Ambisome)<br>☐ Intralesional meglumine antimoniate (Glucantime)<br>☐ Intramuscular/intravenous meglumine antimoniate (Glucantime)<br>☐ Miltefosine<br>☐ Pentamidine<br>☐ Intralesional sodium stibogluconate (SSG)<br>☐ Intramuscular/intravenous sodium stibogluconate (SSG)<br>☐ Ketoconazole<br>☐ Fluconazole<br>☐ Ointment paromomycin (15%) and methylbenzethonium (12%)<br>☐ Cryotherapy<br>☐ Thermotherapy<br>☐ Wash/dressing<br>☐ Systemic antibiotics<br>☐ Topic antibiotics<br>☐ Other<br>☐ None<br>☐ Unknown

Cutaneous involvement

Date of onset of cutaneous lesions	______________________________<br>(If unknown, provide the best estimate)
Number of cutaneous lesions at the time of initial visit at your center	______________________________
Maximum diameter of the largest cutaneous lesion (cm)	______________________________<br>(In case of ulcers, please provide the longest diameter of the ulcerated area)
Surface area of the largest cutaneous lesion (cm^2) (optional)	______________________________<br>(Estimate by multiplying the two longest perpendicular diameters (Length × Width) in cm)

Instructions to measure the diameters in order to estimate area (optional)

Ulcerated lesion Indurated area of a non-ulcerated lesion

Source: Ranasinghe S,et al. (2024). Development of a Core Outcome Measure Instrument; "LeishCOM_LCL", for Localised Cutaneous Leishmaniasis. PLOS Neglected Tropical Diseases, 18(8): e0012393. https://doi.org/10.1371/journal.pntd.0012393

Location of the largest lesion	☐ Head/neck<br>☐ Face<br>☐ Ears<br>☐ Eyes<br>☐ Nose<br>☐ Lips<br>☐ Back<br>☐ Thorax<br>☐ Abdomen<br>☐ Buttocks<br>☐ Genitals or perineum<br>☐ Joints<br>☐ Upper limbs<br>☐ Hands<br>☐ Fingers<br>☐ Lower limbs<br>☐ Feet<br>☐ Toes<br>☐ Other<br>(Consider each location as mutually exclusive with the other ones (i.e.,. if there is only 1 lesion located on the face, select "yes" for "face" but no for "head/neck"))
Other location(s)	__________________________________
Largest lesion features	☐ Ulcer<br>☐ Papule<br>☐ Nodule<br>☐ Squamous plaque<br>☐ Dry crust<br>☐ Sporotrichoid lesions<br>☐ Verrucous lesion<br>☐ Vegetative (exophytic) lesion<br>☐ Satellite lesions<br>☐ Scar<br>☐ Other
Please specify the type of lesion	__________________________________
Maximum diameter of the SECOND largest cutaneous lesion (cm)	__________________________________<br>(In case of ulcers, please provide the longest diameter of the ulcerated area)
Surface area of the SECOND largest cutaneous lesion (cm^2) (optional)	__________________________________<br>(Estimate by multiplying the two longest perpendicular diameters (Length × Width) in cm)

Location of SECOND largest lesion	☐ Head/neck<br>☐ Face<br>☐ Ears<br>☐ Eyes<br>☐ Nose<br>☐ Lips<br>☐ Back<br>☐ Thorax<br>☐ Abdomen<br>☐ Buttocks<br>☐ Genitals or perineum<br>☐ Joints<br>☐ Upper limbs<br>☐ Hands<br>☐ Fingers<br>☐ Lower limbs<br>☐ Feet<br>☐ Toes<br>☐ Other<br>(Consider each location as mutually exclusive with the other ones (i.e.,. if there is only 1 lesion located on the face, select "yes" for "face" but no for "head/neck"))
Other location(s)	__________________________________
SECOND largest lesion features	☐ Ulcer<br>☐ Papule<br>☐ Nodule<br>☐ Squamous plaque<br>☐ Dry crust<br>☐ Sporotrichoid lesions<br>☐ Verrucous lesion<br>☐ Vegetative (exophytic) lesion<br>☐ Satellite lesions<br>☐ Scar<br>☐ Other
Please specify the type of lesion	__________________________________
Maximum diameter of the THIRD largest cutaneous lesion (cm)	__________________________________<br>(In case of ulcers, please provide the longest diameter of the ulcerated area)
Surface area of the THIRD largest cutaneous lesion (cm^2) (optional)	__________________________________<br>(Estimate by multiplying the two longest perpendicular diameters (Length × Width) in cm)

Location of THIRD largest lesion	☐ Head/neck<br>☐ Face<br>☐ Ears<br>☐ Eyes<br>☐ Nose<br>☐ Lips<br>☐ Back<br>☐ Thorax<br>☐ Abdomen<br>☐ Buttocks<br>☐ Genitals or perineum<br>☐ Joints<br>☐ Upper limbs<br>☐ Hands<br>☐ Fingers<br>☐ Lower limbs<br>☐ Feet<br>☐ Toes<br>☐ Other<br>(Consider each location as mutually exclusive with the other ones (i.e.,. if there is only 1 lesion located on the face, select "yes" for "face" but no for "head/neck"))
Other location(s)	__________________________________
THIRD largest lesion features	☐ Ulcer<br>☐ Papule<br>☐ Nodule<br>☐ Squamous plaque<br>☐ Dry crust<br>☐ Sporotrichoid lesions<br>☐ Verrucous lesion<br>☐ Vegetative (exophytic) lesion<br>☐ Satellite lesions<br>☐ Scar<br>☐ Other
Please specify the type of lesion	__________________________________

Location of other lesions (select ALL that apply)	☐ Head/neck<br>☐ Face<br>☐ Ears<br>☐ Eyes<br>☐ Nose<br>☐ Lips<br>☐ Back<br>☐ Thorax<br>☐ Abdomen<br>☐ Buttocks<br>☐ Genitals or perineum<br>☐ Joints<br>☐ Upper limbs<br>☐ Hands<br>☐ Fingers<br>☐ Lower limbs<br>☐ Feet<br>☐ Toes<br>☐ Other<br>(Consider each location as mutually exclusive with the other ones (i.e.,. if there is only 1 lesion located on the face, select "yes" for "face" but no for "head/neck"))
Other location(s)	______________________________
Other lesion(s) features (select ALL that apply)	☐ Ulcer<br>☐ Papule<br>☐ Nodule<br>☐ Squamous plaque<br>☐ Dry crust<br>☐ Sporotrichoid lesions<br>☐ Verrucous lesion<br>☐ Vegetative (exophytic) lesion<br>☐ Satellite lesions<br>☐ Scar<br>☐ Other
Please specify the type of lesion	______________________________
Can you provide a picture of the main cutaneous lesion(s) at initial visit?	
Which symptom(s) were associated with the cutaneous lesion(s)?	☐ Pain<br>☐ Pruritus<br>☐ Burning sensation<br>☐ Functional limitation (e.g., difficulty moving mouth/eye)<br>☐ Other (specify)
Please specify other symptoms	______________________________

Mucosal involvement

Date of onset of mucosal lesions	__________________________________<br>(If unknown, provide the best estimate)
Number of mucosal lesions at the time of initial visit at your center	__________________________________
Maximum diameter of the largest mucosal lesion (mm)	__________________________________<br>(In case of ulcers, please provide the longest diameter of the ulcerated area)
Location of mucosal lesions (select ALL that apply)	☐ Nasal mucosa / septum<br>☐ Oral cavity (palate, uvula, tonsils)<br>☐ Pharynx (posterior wall)<br>☐ Larynx (including epiglottis and vocal cords)<br>☐ Other site (specify)
Other location(s) for mucosal lesions	__________________________________
Can you provide a picture of the main mucosal lesion(s) at initial visit?	
Severity of symptoms of mucosal leishmaniasis	☐ No symptoms<br>☐ Mild - Symptoms confined to the nose (e.g., nasal obstruction)<br>☐ Moderate - Odynophagia, dysphonia, and/or mild respiratory distress (no dyspnea or airway threat)<br>☐ Severe - Severe respiratory distress (e.g., dyspnea, stridor, risk of airway obstruction)<br>☐ Unknown

Severity classification of mucosal leishmaniasis

Classification	Definition
Mild	Involvement of ≤2 mucous sites
Moderate	Involvement of >2 mucous sites, with mild or no respiratory distress
Severe	Same as moderate, plus severe respiratory distress

Based on Llanos-Cuentas A et al. Efficacy of sodium stibogluconate alone and in combination with allopurinol for treatment of mucocutaneous leishmaniasis. Clin Infect Dis. 1997 Sep;25(3):677-84. doi: 10.1086/513776.

[Attachment: "Llanos-Cuentas et al.pdf"]

Please indicate severity of mucosal involvement according to provided classification	◯ Mild<br>◯ Moderate<br>◯ Severe<br>◯ Unknown

Other clinical findings

Was there any palpable lymphadenopathy draining the area of the lesion?	◯ Yes<br>◯ No<br>◯ Unclear<br>◯ Not assessed<br>(Indicate whether regional lymph nodes were evaluated. Select "Unclear" if evaluation status is uncertain, or "Not assessed" if no attempt was made to examine them.)
Were there signs of bacterial superinfection?	◯ No<br>◯ Yes<br>◯ Unknown<br>(Purulent drainage, local cellulitis...)
Were there other relevant findings in physical examination?	______________________________
Weight (kg)	______________________________
Height (cm)	______________________________
Skin phototype	◯ Phototype I-II (very fair to fair skin)<br>◯ Phototype III-IV (medium to olive skin)<br>◯ Phototype V-VI (brown to dark brown/black skin)<br>◯ Unknown

Underlying conditions

Any underlying conditions?	◯ Yes<br>◯ No<br>◯ Unknown
Which one(s)?	☐ Hypertension<br>☐ Dyslipidemia<br>☐ Diabetes mellitus<br>☐ Cardiovascular disease (ischemic heart disease, myocardial infarction, heart failure, stroke...)<br>☐ Respiratory disease (asthma, COPD, interstitial lung disease...)<br>☐ Chronic kidney disease<br>☐ Chronic liver disease<br>☐ Immunocompromising condition<br>☐ Pregnancy<br>☐ Other<br>(Select all that apply)
Specify other underlying condition	______________________________

Type of immunocompromising condition	☐ HIV infection<br>☐ Solid organ transplantation<br>☐ Hematological malignancy / Stem cell transplantation<br>☐ Conventional immunosuppressants (e.g., corticosteroids, methotrexate)<br>☐ Biological immunosuppressants (e.g., anti-TNF, IL-6 inhibitors)<br>☐ Other (specify)<br>☐ Unknown
HIV infection	◯ CD4 count < 200/mm3<br>◯ CD4 count >200/mm3/AIDS events<br>◯ Unknown
Which organ?	☐ Kidney<br>☐ Liver<br>☐ Heart<br>☐ Lung<br>☐ Pancreas
Hematological malignancy / Stem cell transplantation	☐ Acute or chronic leukemia<br>☐ Lymphomas (Hodgkin and non-Hodgkin)<br>☐ Multiple myeloma<br>☐ Myelodysplastic syndromes<br>☐ Autologous HSCT<br>☐ Allogeneic HSCT<br>☐ Other
Conventional immunosuppressants	☐ Corticosteroids<br>☐ Antimetabolites: Azathioprine, methotrexate, leflunomide<br>☐ Calcineurin inhibitors: Cyclosporine, tacrolimus<br>☐ Alkylating agents: Cyclophosphamide, chlorambucil<br>☐ mTOR inhibitors: Sirolimus, everolimus<br>☐ Other
Biological immunosuppressants	☐ TNF-α inhibitors: Infliximab, adalimumab, etanercept...<br>☐ IL-6 inhibitors: Tocilizumab, sarilumab...<br>☐ B-cell depleting agents: Rituximab, obinutuzumab...<br>☐ IL-1 blockers: Anakinra, canakinumab...<br>☐ Integrin inhibitors: Natalizumab, vedolizumab<br>☐ JAK inhibitors: Tofacitinib, baricitinib...<br>☐ Other
Which other(s) immunocompromising condition/immunosuppressant drugs?	______________________________
Comments	______________________________

Microbiological diagnosis

Sample type	☐ Skin scraping<br>☐ Skin biopsy<br>☐ Skin aspirate<br>☐ Curettage<br>☐ Swab<br>☐ Mucosal biopsy<br>☐ Other
Specify other sample type	__________________________________
**Diagnostic test**	
PCR test	◯ Negative<br>◯ Positive<br>◯ Inconclusive<br>◯ Not done<br>◯ Unknown
Culture	◯ Negative<br>◯ Positive<br>◯ Inconclusive<br>◯ Not done<br>◯ Unknown
Microscopy	◯ Negative<br>◯ Positive<br>◯ Inconclusive<br>◯ Not done<br>◯ Unknown
Histopathology	◯ Negative<br>◯ Positive<br>◯ Inconclusive<br>◯ Not done<br>◯ Unknown
Histopathological features	☐ Presence of amastigotes<br>☐ Granulomatous inflammation<br>☐ Non-specific chronic inflammation (no granulomas)<br>☐ Necrotizing inflammation<br>☐ Other (specify)
Please specify other histopathological features	__________________________________

Leishmania species

Leishmania species

- [ ] Leishmania subgenus
- [ ] L.L. tropica
- [ ] L.L. major
- [ ] L.L. aethiopica
- [ ] L.L. donovani complex
- [ ] L.L. donovani
- [ ] L.L. infantum/chagasi (synonyms)
- [ ] L.L. mexicana complex
- [ ] L.L. mexicana
- [ ] L.L. venezuelensis
- [ ] L.L. amazonensis
- [ ] Viannia (subgenus)
- [ ] L.V. braziliensis complex
- [ ] L.V. braziliensis
- [ ] L.V. peruviana
- [ ] L.V. guyanensis complex
- [ ] L.V. guyanensis
- [ ] L.V. panamensis
- [ ] Other
- [ ] Leishmania species not identified

(Select the identified species. If only the complex or subgenus is known, select that instead. Use "Other" to specify unlisted species, or "Leishmania species not identified" if unknown.)

Specify other Leishmania species

____

Date of diagnosis confirmation

____

How was the case confirmed? (select all that apply)

- [ ] Confirmed by parasitology
- [ ] Confirmed by PCR
- [ ] Confirmed by histopathology
- [ ] Confirmed clinically
- [ ] Unknown

(Select all confirmation methods that contributed to the final diagnosis. This is not limited to the first test performed.)

Which criteria supported the clinical diagnosis?

- [ ] Typical lesion morphology (e.g., ulcer with raised edges, central crust)
- [ ] Compatible lesion location (exposed areas, mucosal involvement)
- [ ] Chronic evolution (more than 2-4 weeks without healing)
- [ ] Compatible epidemiological exposure (residence or travel to endemic area)
- [ ] Family or community cases (cluster)
- [ ] Absence of alternative diagnosis (e.g., no signs of bacterial or fungal infection)
- [ ] Prior similar lesions in the patient with known leishmaniasis
- [ ] Other criteria (specify below)

(Select ALL that apply)

Please describe other criteria that support the diagnosis

____

Was CL/MCL the first diagnosis to be considered?	◯ Yes<br>◯ No (specify which diagnosis was first considered)<br>◯ Unknown
Which was the first suspicion among differential diagnosis?	◯ Bacterial skin infection<br>◯ Fungal skin infection<br>◯ Mycobacterial skin infection<br>◯ Cutaneous carcinoma<br>◯ Inflammatory dermatosis<br>◯ Trauma or insect bite<br>◯ Other parasitic disease (specify)<br>◯ Other (specify)
Specify which diagnosis was first considered	__________________________________

Bacterial superinfection

Was a bacterial culture performed on a sample taken from the lesion?	◯ No<br>◯ Yes, negative result<br>◯ Yes, positive result (specify identified microorganism)<br>◯ Unknown
Which microorganism(s) was identified?	◯ Staphylococcus aureus<br>◯ Streptococcus pyogenes (Group A Streptococcus)<br>◯ Pseudomonas aeruginosa<br>◯ Escherichia coli<br>◯ Klebsiella pneumoniae<br>◯ Proteus spp.<br>◯ Polymicrobial (specify)<br>◯ Other (specify)
Please specify bacteria in case of polymicrobial or other	__________________________________
Comments	____

Initial treatment

Previous treatment

Had the patient received any previous treatment for other considered diagnosis?	◯ Yes (specify)<br>◯ No<br>◯ Unknown
Specify which treatment the patient had previously received for other considered diagnosis	__________________________
Had the patient received any previous treatment for leishmaniasis?	◯ Yes<br>◯ No<br>◯ Unknown
Do you know which treatment had the patient previously received?	☐ Liposomal Amphotericin B (Ambisome)<br>☐ Intralesional meglumine antimoniate (Glucantime)<br>☐ Intramuscular/intravenous meglumine antimoniate (Glucantime)<br>☐ Miltefosine<br>☐ Pentamidine<br>☐ Intralesional sodium stibogluconate (SSG)<br>☐ Intramuscular/intravenous sodium stibogluconate (SSG)<br>☐ Ketoconazole<br>☐ Fluconazole<br>☐ Ointment paromomycin (15%) and methylbenzethonium (12%)<br>☐ Cryotherapy<br>☐ Thermotherapy<br>☐ Wash/dressing<br>☐ Systemic antibiotics<br>☐ Topical antibiotics<br>☐ Other<br>☐ Unknown
Specify other treatment	__________________________

Current episode

Based on the current evaluation, how would you classify this case?

Please select ALL the items that apply for this case

Criteria for Complex CL
Size

>4 cm

______

Number of lesions

>4

______

Location

Ears, eyelids, nose, lips, joints, fingers, toes

______

Mucosal involvement

Yes

______

Immunosuppression

Yes

______

Failure to local treatment

Yes

______

Disseminated CL/Leishmaniasis recidivans

Yes

______

Subcutaneous nodules

Yes

______

Lymphatic spread

Yes

______

Based on Vandeputte M, van Henten S, van Griensven J, Huits R, Van Esbroeck M, Van der Auwera G, Cnops L, Bottieau E. Epidemiology, clinical pattern and impact of species-specific molecular diagnosis on management of leishmaniasis in Belgium, 2010-2018: A retrospective study. Travel Med Infect Dis. 2020

[Attachment: "1-s2.0-S1477893920303811-main.pdf"]

◯ Simple CL
◯ Complex CL
◯ Unknown

Leishmania major, simple CL

Spontaneous cure rate is 40-90% at 3 months and close to 100% at 12 months. Consider observation/simple wound care if few, small and non-disturbing lesions.

Local treatment.

First-line treatment
- Intralesional infiltrations of antimonials (2-3 ml Glucantime® 1-3x/week up to 4-6 administrations) AND/OR cryotherapy (2 cycles of 10-30 seconds): cure rate of 70-90% for any method (slightly higher if both methods are combined).

Alternative regimens
- Local heat therapy (50°C for 30 seconds): Thermomed® (expensive device, not readily available).
- 15% paromomycin/12% methylbenzethonium chloride ointment BID for 10-20 days: not available in Belgium; Leishcutan® (Teva) 350 euros on internet

NB: evidence grade A for all regimens; similar efficacy

Leishmania major, complex CL

- Meglumine antimoniate 20 mg SbV/kg od slow iv x 10(-14) days (cure rate: 50-85%; grade D).
- Miltefosine 50 mg po tid x 28 days (cure rate: 85-100%; grade B).
- L-AmB total dose 20 mg/kg; 3 mg/kg Days 1 - 5 and 10 (only some case reports; grade D); experts increasingly recommend a 4- or 5-day regimen (not interrupted) for ease of use. NB: fluconazole 200 mg po bid x 6 weeks: cure rate of 80% in one small RCT in Saudi Arabia; similar efficacy never reproduced elsewhere). For sure not a first choice.

There is no clear first-line regimen, except that antimoniates have been more used and are readily available.

Did the patient receive a treatment course for Leishmania at first visit?	◯ Yes<br>◯ No
Was the treatment administered as inpatient or outpatient?	◯ Inpatient<br>◯ Outpatient<br>◯ Mixed (part inpatient, part outpatient)<br>◯ Unknown<br>(Day hospital or ambulatory treatment (without overnight stay) should be classified as Outpatient.)
Were safety investigations performed before starting treatment?	☐ ECG<br>☐ Liver function tests (ALT/AST)<br>☐ Renal function tests (creatinine, urea)<br>☐ Full blood count<br>☐ Pregnancy test<br>☐ Electrolytes (K+, Na+, etc.)<br>☐ Other (specify)<br>☐ None<br>☐ Unknown<br>(Select all that apply)
Specify other investigations performed before treatment initiation	__________________________

Were any test results abnormal?	◯ Yes (specify)<br>◯ No<br>◯ Unknown
Specify which abnormal results were identified	__________________________________
Start date of treatment	__________________________________
Treatment regimen for Leishmania	☐ Liposomal Amphotericin B (Ambisome)<br>☐ Meglumine antimoniate (Glucantime)<br>☐ Miltefosine<br>☐ Pentamidine<br>☐ Sodium stibogluconate (SSG)<br>☐ Ketoconazole<br>☐ Fluconazole<br>☐ Ointment paromomycin (15%) and methylbenzethonium (12%)<br>☐ Cryotherapy<br>☐ Thermotherapy<br>☐ Wash/dressing<br>☐ Other<br>☐ Unknown
Other Leishmania treatment regimen	__________________________________

Liposomal Amphotericin B

Start date of Liposomal Amphotericin B	__________________________________
How was Liposomal Amphotericin B administered?	☐ Intravenous<br>☐ Other
Specify other route of administration for Liposomal Amphotericin B	__________________________________
What dose of intravenous Liposomal Amphotericin B was used?	◯ 20mg/kg (3 mg/kg/day D1-5, 10)<br>◯ 20mg/kg (3 mg/kg/day D1-5, 14, 21)<br>◯ 20mg/kg (4 mg/kg/day D1-5)<br>◯ 20mg/kg (3 mg/kg/day D1-7)<br>◯ 40mg/kg (4mg/kg/day D1-10)<br>◯ 40mg/kg (4mg/kg/day D1-5, 10, 17, 24, 31, 38)<br>◯ Other<br>◯ Unknown
Specify intravenous Liposomal Amphotericin B dose	__________________________________
Specify liposomal amphotericin B dose administered by other route	__________________________________

Meglumine antimoniate

How was meglumine antimoniate (Glucantime) administered?
- ☐ Intralesional
- ☐ Intramuscular
- ☐ Intravenous

Start date of intralesional meglumine antimoniate
____

How many intralesional meglumine antimoniate (Glucantime) injections were administered?
____

How often were intralesional meglumine antimoniate (Glucantime) injections administered?
- ◯ Every 5 days
- ◯ Every 7 days
- ◯ Every 2 weeks
- ◯ Every 3 weeks
- ◯ Other

Please specify how often were intralesional meglumine antimoniate administered
____

Start date of intramuscular/intravenous meglumine antimoniate
____

What was the intended dose of intravenous/intramuscular meglumine antimoniate (Glucantime)? (mg SbV/Kg/day)
____
(Enter the prescribed weight-based dose, even if the actual dose was rounded)

What was the total daily dose of meglumine antimoniate (Glucantime) actually administered? (mg SbV/day)
____
(Enter the exact dose given per day, including any rounding or dose capping)

How long was intravenous/intramuscular meglumine antimoniate (Glucantime) administered? (days)
____

Miltefosine

Start date of miltefosine
____

What dose of miltefosine was used? (mg/Kg/day)
____

How long was miltefosine administered? (days)
____

Pentamidine

Start date of pentamidine

____

What was the prescribed daily dose of pentamidine? (mg/day)

____

How often was pentamidine administered?

- ◯ Every 2 days
- ◯ Every 7 days
- ◯ Other

Specify pentamidine administration frequency

____

How many pentamidine doses were administered?

____

Sodium stibogluconate

How was sodium stibogluconate administered?

- ☐ Intralesional
- ☐ Intramuscular
- ☐ Intravenous

Start date of intralesional sodium stibogluconate

____

How many intralesional sodium stibogluconate injections were administered?

____

How often were intralesional sodium stibogluconate injections administered?

- ◯ Every 5 days
- ◯ Every 7 days
- ◯ Every 2 weeks
- ◯ Every 3 weeks
- ◯ Other

Please specify how often were intralesional meglumine antimoniate administered

____

Start date of intramuscular/intravenous sodium stibogluconate

____

What was the intended dose of intravenous/intramuscular sodium stigobluconate? (mg SbV/kg/day)

____
(Enter the prescribed weight-based dose, even if the actual dose was rounded.)

What was the total daily dose of intravenous/intramuscular sodium stigobluconate actually administered? (mg SbV/day)

____
(Enter the exact dose given per day, including any rounding or dose capping)

How long was intravenous/intramuscular sodium stibogluconate administered? (days)

____

Ketoconazole

Start date of ketoconazole ______________________

What dose of ketoconazole was used? (mg/day) ______________________

How long was ketoconazole administered? (days) ______________________

Fluconazole

Start date of fluconazole ______________________

What dose of fluconazole was used? (mg/day) ______________________

How long was fluconazole administered? (days) ______________________

Topical paromomycin

Start date of topical paromomycin ______________________

How long was topical paromomycin applied? (days) ______________________

How often was topical paromomycin applied?
- ◯ Once a day
- ◯ Every 12h
- ◯ Every 8h
- ◯ Other

Specify frequency of topical paromomycin administration ______________________

Cryotherapy

Start date of cryotherapy ______________________

How many cryotherapy sessions (treatment visits) were performed? ______________________
(Enter the total number of cryotherapy treatment visits, regardless of the number of freeze-thaw cycles performed at each session)

How many freeze-thaw cycles were performed per treatment session (visit)? ______________________
(A treatment session refers to one visit. A cycle consists of one freeze followed by complete thawing. Enter the usual number applied per session.)

How often were cryotherapy sessions performed?	◯ Every week<br>◯ Every 2 weeks<br>◯ Every 3 weeks<br>◯ Other
Specify how often was cryotherapy performed	______________________

Thermotherapy

Start date of thermotherapy	______________________
Which type of thermotherapy was used for treatment?	☐ Radiofrequency thermotherapy (e.g., ThermoMed)<br>☐ Hand-held exothermic crystallization (e.g., HECT-CL)<br>☐ Infrared thermotherapy (e.g., IR heater device)<br>☐ Microwave thermotherapy (e.g., 2.45 GHz device)<br>☐ Laser therapy (e.g., CO_2 laser)<br>☐ Ultrasound therapy (e.g., handheld therapeutic ultrasound)<br>☐ Hot water therapy (e.g., warm water bag or hydrotherapy)<br>☐ Other (specify)<br>☐ Unknown<br>(If more than one was applied, select all. A follow-up question will ask for the specific device or subtype.)
Specify other type of thermotherapy	______________________
Specify the RF thermotherapy device used	◯ ThermoMed<br>◯ Other RF device (specify)
Specify other RF device	______________________
Specify the hand-held exothermic crystallization device used	◯ HECT-CL<br>◯ Other exothermic device (specify)
Specify other Hand-held exothermic crystallization device	______________________
Specify the type of laser therapy used	◯ CO_2 laser<br>◯ Pulsed dye laser (PDL)<br>◯ Nd:YAG laser<br>◯ Erbium glass laser<br>◯ Diode laser<br>◯ Intravenous laser blood irradiation (ILBI)<br>◯ Other laser type (specify)
Specify other type of laser therapy	______________________
Specify the ultrasound therapy device used	◯ Standard handheld therapeutic ultrasound device<br>◯ Other (specify)

Specify other ultrasound device

____

Specify details on how the thermotherapy was performed (e.g., method, device, manufacturer)

____

(Describe the procedure, duration, number of sessions, temperature used, and manufacturer/model if available (e.g., hot pack type, infrared lamp, microwave device).)

How many thermotherapy sessions (treatment visits) were performed?

____

How often was thermotherapy performed?

- ◯ Every week
- ◯ Every 2 weeks
- ◯ Every 3 weeks
- ◯ Other

Specify how often was thermotherapy performed

____

Combination therapy

Combination therapy?

- ◯ Yes
- ◯ No
- ◯ Unknown

Timing of combination therapy administration

- ◯ Concomitant administration (started at the same time)
- ◯ Consecutive administration without gap (second treatment started immediately after first ended)
- ◯ Consecutive administration with gap (second treatment started after a time gap)
- ◯ Unknown

(Consecutive administration example: first cryotherapy, then immediately or after a time gap amphotericin B)

Which combination treatment was used?

- ◯ Cryotherapy + Intralesional meglumine antimoniate (Glucantime)
- ◯ Liposomal Amphotericin B (Ambisome) + Intralesional meglumine antimoniate (Glucantime)
- ◯ Liposomal Amphotericin B (Ambisome) + Cryotherapy
- ◯ Liposomal Amphotericin B (Ambisome) + Miltefosine
- ◯ Intravenous meglumine antimoniate + pentoxifylline
- ◯ Other (specify)

Specify other treatment combination

____

Please indicate the order in which the treatments were administered. If treatments were given at the same time, check the same column for each.

	First	Second	Third
Liposomal Amphotericin B (Ambisome)	☐	☐	☐
Intralesional meglumine antimoniate (Glucantime)	☐	☐	☐
IV/IM Meglumine antimoniate (Glucantime)	☐	☐	☐
Miltefosine	☐	☐	☐
Pentamidine	☐	☐	☐
Intralesional sodium stibogluconate (SSG)	☐	☐	☐
IV/IM sodium stibogluconate (SSG)	☐	☐	☐
Ketoconazole	☐	☐	☐
Fluconazole	☐	☐	☐
Ointment paromomycin (15%) and methylbenzethonium (12%)	☐	☐	☐
Cryotherapy	☐	☐	☐
Thermotherapy	☐	☐	☐
Other	☐	☐	☐

Was the initial treatment course completed as prescribed?

- ◯ Yes - Completed full treatment course as prescribed
- ◯ No - Did not complete treatment
- ◯ Partial - Completed most, but not full prescribed course
- ◯ Unknown

If not completed, what was the main reason?

- ◯ Adverse event
- ◯ Poor compliance (non-adherence)
- ◯ Loss to follow-up
- ◯ Early stop due to lack of response
- ◯ Early clinical cure (treatment stopped due to complete or rapid response before treatment completion)
- ◯ Other (specify)

Specify other reason why treatment was not completed as prescribed

____

Bacterial superinfection treatment

Which treatment was administered for bacterial superinfection?

- ☐ Systemic antibiotics
- ☐ Topical antibiotics
- ☐ None
- ☐ Unknown

Start date of antibiotic administration

____

Which systemic antibiotic was administered?	◯ Anti-staphylococcal penicillin (e.g., cloxacillin, nafcillin)<br>◯ Broad-spectrum penicillin (e.g., amoxicillin-clavulanate, piperacillin-tazobactam)<br>◯ First/second generation cephalosporin (e.g., cefazolin, cephalexin, cefuroxime)<br>◯ Other beta-lactam antibiotic<br>◯ Macrolide (e.g., azithromycin, clarithromycin)<br>◯ Quinolone (e.g., moxifloxacin, levofloxacin)<br>◯ Other (specify)<br>◯ None
Specify other systemic antibiotic	__________________________________
Duration of systemic antibiotic treatment (days)	__________________________________
Which topical antibiotic was administered?	◯ Fusidic acid<br>◯ Mupirocin<br>◯ Gentamicin<br>◯ Neomycin<br>◯ Clindamycin<br>◯ Erythromycin<br>◯ Metronidazole<br>◯ Other (specify)
Specify other topical antibiotic	__________________________________
Duration of topical antibiotic treatment (days)	__________________________________

Other treatments

Did you prescribe other concomitant treatment?	◯ Yes<br>◯ No<br>◯ Unknown
Which other treatments were prescribed?	☐ Paracetamol<br>☐ NSAIDs (e.g. ibuprofen, naproxen, celecoxib)<br>☐ Antihistamines (e.g., loratadine, diphenhydramine)<br>☐ Topical corticosteroids (e.g., hydrocortisone, betamethasone)<br>☐ Systemic corticosteroids (e.g., prednisone, dexamethasone)<br>☐ Opioids (e.g. tramadol, morphine, oxycodone, fentanyl)<br>☐ Pentoxifylline<br>☐ Other
Specify other concomitant treatment	__________________________________
Comments	____

Initial treatment-related adverse events

⚠

Treatment-Related Adverse Events - Important Instructions

This form is repeatable. Use it to record each individual adverse event (AE) observed during or after treatment.

 How to repeat the form:

You can create a new instance of this form in either of the following ways:

- From the Record Home Page, click the button located next to the status icon of the Treatment-related adverse events form.
- Within the form itself, click the "+ Add new" button found at the top of the page under the "Current Instance" menu. For each adverse event, record:

- Suspected treatment(s) involved
- Type of side event
- Date of onset and resolution
- Severity and clinical outcome
- Any action taken (e.g., dose modification, treatment stopped)

☑

This structure allows accurate tracking and linkage of AEs to specific treatments.

⚠

If no treatment has been registered for the patient, the adverse event instrument will remain empty. In that case, no fields need to be completed and the form should be left blank.

Side effects of Leishmania treatment

Was a treatment-related adverse event registered?	◯ Yes<br>◯ No<br>◯ Unknown

Treatment most likely associated with this adverse event	☐ Liposomal Amphotericin B (Ambisome)<br>☐ Intralesional meglumine antimoniate (Glucantime)<br>☐ Intramuscular/intravenous meglumine antimoniate (Glucantime)<br>☐ Miltefosine<br>☐ Pentamidine<br>☐ Intralesional sodium stibogluconate (SSG)<br>☐ Intramuscular/intravenous sodium stibogluconate (SSG)<br>☐ Ketoconazole<br>☐ Fluconazole<br>☐ Ointment paromomycin (15%) and methylbenzethonium (12%)<br>☐ Cryotherapy<br>☐ Thermotherapy<br>☐ Systemic antibiotics<br>☐ Topical antibiotics<br>☐ Other<br>☐ Unknown
Which type of side effect?	◯ Local side effect<br>◯ Systemic side effect<br>◯ Unknown
Date of side effect onset	________________________________
Timing of local side effects onset	◯ On same day<br>◯ < 3 days after application<br>◯ 3-7 days after application<br>◯ >7 days
Describe local side effect	☐ Pain at lesion or injection site<br>☐ Redness at lesion or injection site<br>☐ Swelling at lesion or injection site<br>☐ Itching at lesion or injection site<br>☐ Burning sensation at lesion or injection site<br>☐ Ulceration or worsening of local wound<br>☐ Localized infection (clinical suspicion or culture-confirmed)<br>☐ Other (specify)
Describe other local side effects	________________________________

Type of systemic side effects	◯ Dermatologic toxicity<br>◯ Hematologic toxicity<br>◯ Metabolic or electrolyte disturbance<br>◯ Hypersensitivity or anaphylaxis<br>◯ Cardiotoxicity<br>◯ Hepatotoxicity<br>◯ Renal toxicity<br>◯ Gastrointestinal symptoms<br>◯ Neurologic or neuropsychiatric toxicity<br>◯ Pancreatitis<br>◯ Infusion-related reaction<br>◯ Other (specify)<br>◯ Unknown<br>(Choose the type of adverse event. Based on your selection, a second field will appear to detail the specific clinical manifestation (e.g., rash, neutropenia, QT prolongation).)
Describe other systemic side effects	__________________________
Specify dermatologic toxicity	☐ Rash (maculopapular, erythematous, etc.)<br>☐ Skin ulceration<br>☐ Pruritus<br>☐ Dry skin<br>☐ Hyperpigmentation or discoloration<br>☐ Alopecia<br>☐ Other (specify)
Specify other dermatologic toxicity	__________________________
Specify hematological toxicity	☐ Anemia<br>☐ Leukopenia<br>☐ Neutropenia<br>☐ Lymphopenia<br>☐ Thrombocytopenia<br>☐ Pancytopenia<br>☐ Other (specify)
Specify other hematological toxicity	__________________________
Specify metabolic or electrolyte abnormality	☐ Hypokalemia<br>☐ Hyponatremia<br>☐ Hyperkalemia<br>☐ Hyperglycemia<br>☐ Hypoglycemia<br>☐ Acidosis<br>☐ Alkalosis<br>☐ Other (specify)
Specify other metabolic or electrolyte abnormality	__________________________

Specify hypersensitivity reaction	☐ Anaphylaxis<br>☐ Urticaria<br>☐ Facial or airway edema<br>☐ Bronchospasm<br>☐ Flushing<br>☐ Hypotension<br>☐ Other (specify)
Specify other hypersensitivity reaction	__________________________________
Specify cardiac event	☐ QT interval prolongation<br>☐ Bradycardia<br>☐ Sinus or supraventricular tachycardia<br>☐ Atrial fibrillation or flutter<br>☐ AV block or conduction abnormality<br>☐ Myocarditis or pericarditis<br>☐ Heart failure<br>☐ Chest pain (suspected cardiac origin)<br>☐ Other (specify)
Specify other cardiac toxicity	__________________________________
Specify type of liver test abnormality or clinical finding	☐ Elevated ALT and/or AST<br>☐ Elevated ALP<br>☐ Elevated GGT<br>☐ Elevated bilirubin<br>☐ Jaundice<br>☐ Clinical hepatitis symptoms (e.g., fatigue, anorexia)<br>☐ Other (specify)
Specify other hepatotoxicity	__________________________________
Specify renal toxicity	☐ Elevated creatinine (lab increase not meeting AKI criteria)<br>☐ Proteinuria<br>☐ Hematuria<br>☐ Acute kidney injury (AKI) (creatinine ↑ ≥1.5× baseline and/or oliguria)<br>☐ Other (specify)
Specify other renal toxicity	__________________________________
Specify gastrointestinal toxicity	☐ Nausea<br>☐ Vomiting<br>☐ Diarrhea<br>☐ Abdominal pain<br>☐ Mucositis or oral ulcers<br>☐ Constipation<br>☐ Anorexia<br>☐ Other (specify)
Specify other gastrointestinal toxicity	__________________________________

Specify neurological or psychiatric event	☐ Headache<br>☐ Dizziness or vertigo<br>☐ Paresthesia or numbness<br>☐ Seizure<br>☐ Confusion or disorientation<br>☐ Tremor or ataxia<br>☐ Visual disturbances<br>☐ Mood or behavioral changes<br>☐ Other (specify)
Specify other neurological or psychiatric event	__________________________________
Specify infusion-related symptoms	☐ Fever<br>☐ Chills<br>☐ Hypotension<br>☐ Back pain<br>☐ Flushing<br>☐ Dyspnea<br>☐ Other (specify)
Specify other infusion-related symptoms	__________________________________
Severity of the side effect	○ Mild - no interference with activity<br>○ Moderate - some interference, no hospitalization<br>○ Severe - prevents daily activity, may require hospitalization<br>○ Life-threatening - urgent intervention required<br>○ Death<br>○ Unknown
Date of side effect resolution	__________________________________
Date of death	__________________________________
How was the adverse event managed?	☐ No action needed<br>☐ Symptomatic treatment (e.g., antihistamines, NSAIDs)<br>☐ Treatment temporarily interrupted<br>☐ Treatment permanently discontinued<br>☐ Switched to alternative treatment<br>☐ Referred for specialist evaluation or hospitalization<br>☐ Unknown

Regarding suspected treatment-related bacterial superinfection

Was a bacterial culture performed on a sample taken from the lesion?	○ No<br>○ Yes, negative result<br>○ Yes, positive result (specify identified microorganism)<br>○ Unknown

Which microorganism(s) was identified?	◯ Staphylococcus aureus<br>◯ Streptococcus pyogenes (Group A Streptococcus)<br>◯ Pseudomonas aeruginosa<br>◯ Escherichia coli<br>◯ Klebsiella pneumoniae<br>◯ Proteus spp.<br>◯ Polymicrobial (specify)<br>◯ Other (specify)
Please specify bacteria in case of polymicrobial or other	__________________________________
Which treatment was administered for bacterial superinfection?	☐ Systemic antibiotics<br>☐ Topical antibiotics<br>☐ None<br>☐ Unknown
Start date of antibiotic administration	__________________________________
Which systemic antibiotic was administered?	◯ Anti-staphylococcal penicillin (e.g., cloxacillin, nafcillin)<br>◯ Broad-spectrum penicillin (e.g., amoxicillin-clavulanate, piperacillin-tazobactam)<br>◯ First/second generation cephalosporin (e.g., cefazolin, cephalexin, cefuroxime)<br>◯ Other beta-lactam antibiotic<br>◯ Macrolide (e.g., azithromycin, clarithromycin)<br>◯ Quinolone (e.g., moxifloxacin, levofloxacin)<br>◯ Other (specify)<br>◯ None
Specify other systemic antibiotic	__________________________________
Duration of systemic antibiotic treatment (days)	__________________________________
Which topical antibiotic was administered?	◯ Fusidic acid<br>◯ Mupirocin<br>◯ Gentamicin<br>◯ Neomycin<br>◯ Clindamycin<br>◯ Erythromycin<br>◯ Metronidazole<br>◯ Other (specify)
Specify other topical antibiotic	__________________________________
Duration of topical antibiotic treatment (days)	__________________________________

Follow-up visit

 Follow-Up Visit Registration - Important Instructions

This form is repeatable. For each follow-up visit, create a new form entry to document the patient's progress.

 To add a new follow-up visit entry:

You can create a new instance of this form in either of the following ways:

- From the Record Home Page, click the button located next to the status icon of the Follow-up visit form.
- Within the form itself, click the "+ Add new" button found at the top of the page under the "Current Instance" menu. Standard recommended follow-up visits (timing counted from treatment initiation):

- 1st follow-up at day 42 - Initial cure assessment

- 2nd follow-up at day 90 - Initial cure assessment

- 3rd follow-up at 6 months - Final cure assessment

- 4th follow-up at 12 months is recommended for MCL and ML and optional for CL to check for relapse.

Other visits may also be recorded if clinically indicated.

Note: The scheduled time points are recommended and represent the preferred timing for assessments. However, we understand that in clinical practice, exact adherence may not always be possible. A window of ±14 days is acceptable for the 1st and 2nd visits, ±4 weeks for the 3rd visit and ±8 weeks for the 4th visit. If a visit falls outside these windows, it should be recorded as an "Other follow-up visit" rather than as a scheduled visit.

These recommendations are based on Olliaro P et al. Harmonized clinical trial ethodologies for localized cutaneous leishmaniasis and potential for extensive network with capacities for clinical evaluation. PLoS Negl Trop Dis 2018 12(1): e0006141.

Date of follow-up visit

____
(Follow-up visits should be performed approximately at days 42 and 90, and at 6 and 12 months after treatment initiation.)

Which follow-up visit is this?

○ 1st follow-up visit (approximately day 42 from treatment initiation)
○ 2nd follow-up visit (approximately day 90 from treatment initiation)
○ 3rd follow-up visit (approximately 6 months from treatment initiation)
○ 4th follow-up visit (approximately 12 months from treatment initiation)
○ Other

How was this visit done?	○ In person<br>○ Phone call<br>○ Video call (Zoom, Hangouts, WA...)<br>○ Email<br>○ Unknown

Clinical assessment

Clinical response	☐ Cure: complete re-epithelization or flattening<br>☐ Partial improvement<br>☐ No change<br>☐ Clinical worsening/new complications<br>☐ Other (specify)<br>☐ Unknown<br>☐ Lost to follow-up
Please specify other clinical response	__________________________
Features of partial improvement	☐ ≥50% re-epithelization in the largest ulcerated lesion size<br>☐ < 50% re-epithelization in the largest ulcerated lesion size<br>☐ ≥50% flattening in the largest non-ulcerated lesion size<br>☐ < 50% flattening in the largest non-ulcerated lesion size<br>☐ Reduction in induration or erythema<br>☐ Pain or tenderness improved<br>☐ Some lesions completely cured<br>☐ Other<br>(For ulcerated lesions: estimate the % re-epithelization relative to the baseline diameter of the ulcer. Surrounding erythema is not included.)
Signs of clinical worsening	☐ Onset of new lesion(s)<br>☐ Increase in size of existing lesion(s)<br>☐ New mucosal involvement (patients without previous mucosal involvement)<br>☐ Progression of mucosal involvement (e.g. other sites affected)<br>☐ Development of new regional lymphadenopathy<br>☐ Bacterial superinfection<br>☐ Other (specify)
Describe other signs of clinical worsening	__________________________

Features of new lesion(s)	☐ Ulcer<br>☐ Papule<br>☐ Nodule<br>☐ Squamous plaque<br>☐ Dry crust<br>☐ Sporotrichoid lesions<br>☐ Verrucous lesion<br>☐ Vegetative (exophytic) lesion<br>☐ Satellite lesions<br>☐ Scar<br>☐ Other
Please specify the type of lesion	______________________
Date of cure	______________________<br>(If it is unknown, provide the best estimate)
When did the lesion(s) start to improve?	______________________<br>(If it is unknown, provide the best estimate)
Can you provide a picture of the lesion(s) at this visit?	

New treatment episode

Did the patient receive a new treatment course for Leishmania?	◯ Yes<br>◯ No<br>◯ Unknown
Why did the patient receive a new treatment course?	☐ Adverse effects with previous treatment<br>☐ Poor compliance with previous treatment regimen<br>☐ Clinical criteria (worsening, no response, or partial response...)<br>☐ Treatment not available (out of stock, resource limitations...)<br>☐ Other

Reassess the case classification based on the current evaluation.Cases initially considered simple CL may now meet criteria for complex forms. Please select ALL the items that apply on the current evaluation.

Criteria for Complex CL

Size

>4 cm

______

Number of lesions

>4

______

Location

Ears, eyelids, nose, lips, joints, fingers, toes

———

Mucosal involvement

Yes

———

Immunosuppression

Yes

———

Failure to local treatment

Yes

———

Disseminated CL/Leishmaniasis recidivans

Yes

———

Subcutaneous nodules

Yes

———

Lymphatic spread

Yes

———

Based on Vandeputte M, van Henten S, van Griensven J, Huits R, Van Esbroeck M, Van der Auwera G, Cnops L, Bottieau E. Epidemiology, clinical pattern and impact of species-specific molecular diagnosis on management of leishmaniasis in Belgium, 2010-2018: A retrospective study. Travel Med Infect Dis. 2020

[Attachment: "1-s2.0-S1477893920303811-main.pdf"]

	◯ Simple CL<br>◯ Complex CL<br>◯ Unknown
Was the treatment administered as inpatient or outpatient?	◯ Inpatient<br>◯ Outpatient<br>◯ Mixed (part inpatient, part outpatient)<br>◯ Unknown

Were safety investigations performed before starting treatment?	☐ ECG<br>☐ Liver function tests (ALT/AST)<br>☐ Renal function tests (creatinine, urea)<br>☐ Full blood count<br>☐ Pregnancy test<br>☐ Electrolytes (K+, Na+, etc.)<br>☐ Other (specify)<br>☐ None<br>☐ Unknown<br>(Select all that apply)
Specify other investigations performed before treatment initiation	__________________________
Were any test results abnormal?	◯ Yes (specify)<br>◯ No<br>◯ Unknown
Specify which abnormal results were identified	__________________________
Start date of treatment	__________________________
Treatment regimen for Leishmania	☐ Liposomal Amphotericin B (Ambisome)<br>☐ Meglumin antimoniate (Glucantime)<br>☐ Miltefosine<br>☐ Pentamidine<br>☐ Sodium stibogluconate (SSG)<br>☐ Ketoconazole<br>☐ Fluconazole<br>☐ Ointment paromomycin (15%) and methylbenzethonium (12%)<br>☐ Cryotherapy<br>☐ Thermotherapy<br>☐ Wash/dressing<br>☐ Other<br>☐ Unknown
Other treatment regimen for Leishmania	__________________________

Liposomal Amphotericin B

Start date of Liposomal Amphotericin B	__________________________
How was Liposomal Amphotericin B administered?	☐ Intravenous<br>☐ Other
Specify other route of administration for Amphotericin B	__________________________

What dose of intravenous Liposomal Amphotericin B was used?	◯ 20mg/kg (3 mg/kg/day D1-5, 10)<br>◯ 20mg/kg (3 mg/kg/day D1-5, 14, 21)<br>◯ 20mg/kg (4 mg/kg/day D1-5)<br>◯ 20mg/kg (3 mg/kg/day D1-7)<br>◯ 40mg/kg (4mg/kg/day D1-10)<br>◯ 40mg/kg (4mg/kg/day D1-5, 10, 17, 24, 31, 38)<br>◯ Other
Specify intravenous Liposomal Amphotericin B dose	__________________________________
Specify liposomal amphotericin B dose administered by other route	__________________________________

Meglumine antimoniate

How was meglumine antimoniate (Glucantime) administered?	☐ Intralesional<br>☐ Intramuscular<br>☐ Intravenous
Start date of intralesional meglumine antimoniate	__________________________________
How many intralesional meglumine antimoniate (Glucantime) injections were administered?	__________________________________
How often were intralesional meglumine antimoniate (Glucantime) injections administered?	◯ Every 5 days<br>◯ Every 7 days<br>◯ Every 2 weeks<br>◯ Every 3 weeks<br>◯ Other
Specify how often were intralesional meglumine antimoniate injections administered	__________________________________
Start date of intramuscular/intravenous meglumine antimoniate	__________________________________
What was the intended dose of intravenous/intramuscular meglumine antimoniate (Glucantime)? (mg SbV/Kg/day)	__________________________________<br>(Enter the prescribed weight-based dose, even if the actual dose was rounded)
What was the total daily dose of meglumine antimoniate (Glucantime) actually administered? (mg SbV/day)	__________________________________<br>(Enter the exact dose given per day, including any rounding or dose capping)
How long was intravenous/intramuscular meglumine antimoniate (Glucantime) administered? (days)	__________________________________

Miltefosine

Start date of miltefosine ______________________

What was the prescribed daily dose of miltefosine? (mg/day) ______________________

How long was miltefosine administered? (days) ______________________

Pentamidine

Start date of pentamidine ______________________

What dose of pentamidine was used? (mg/Kg/day) ______________________

How often was pentamidine administered?
- ◯ Every 2 days
- ◯ Every 7 days
- ◯ Other

Specify pentamidine administration frequency ______________________

How many pentamidine doses were administered? ______________________

Sodium stibogluconate

How was sodium stibogluconate (SSG) administered?
- ☐ Intralesional
- ☐ Intramuscular
- ☐ Intravenous

Start date of intralesional sodium stibogluconate ______________________

How many intralesional sodium stibogluconate injections were administered? ______________________

How often were intralesional sodium stibogluconate injections administered?
- ◯ Every 5 days
- ◯ Every 7 days
- ◯ Every 2 weeks
- ◯ Every 3 weeks
- ◯ Other

Specify how often were intralesional sodium stibogluconate injections administered ______________________

Start date of intramuscular/intravenous sodium stibogluconate ______________________

What was the intended dose of intravenous/intramuscular sodium stigobluconate? (mg SbV/kg/day) | ______________________________
(Enter the prescribed weight-based dose, even if the actual dose was rounded.)

What was the total daily dose of intravenous/intramuscular sodium stigobluconate actually administered? (mg SbV/day) | ______________________________
(Enter the exact dose given per day, including any rounding or dose capping)

How long was intravenous/intramuscular sodium stibogluconate administered? (days) | ______________________________

Ketoconazole

Start date of ketoconazole | ______________________________

What dose of ketoconazole was used? (mg/day) | ______________________________

How long was ketoconazole administered? (days) | ______________________________

Fluconazole

Start date of fluconazole | ______________________________

What dose of fluconazole was used? (mg/day) | ______________________________

How long was fluconazole administered? (days) | ______________________________

Topical paromomycin

Start date of topical paromomycin | ______________________________

How long was topical paromomycin applied? (days) | ______________________________

How often was topical paromomycin applied?
- ◯ Once a day
- ◯ Every 12h
- ◯ Every 8h
- ◯ Other

Specify frequency of topical paromomycin administration | ______________________________

Cryotherapy

Start date of cryotherapy	__________________________________
How many cryotherapy sessions (treatment visits) were performed?	__________________________________<br>(Enter the total number of cryotherapy treatment visits, regardless of the number of freeze-thaw cycles performed at each session)
How many freeze-thaw cycles were performed per treatment session (visit)?	__________________________________<br>(A treatment session refers to one visit. A cycle consists of one freeze followed by complete thawing. Enter the usual number applied per session.)
How often was cryotherapy performed?	◯ Every week<br>◯ Every 2 weeks<br>◯ Every 3 weeks<br>◯ Other
Specify how often was cryotherapy performed	__________________________________

Thermotherapy

Start date of thermotherapy	__________________________________
Which type of thermotherapy was used for treatment?	☐ Radiofrequency thermotherapy (e.g., ThermoMed)<br>☐ Hand-held exothermic crystallization (e.g., HECT-CL)<br>☐ Infrared thermotherapy (e.g., IR heater device)<br>☐ Microwave thermotherapy (e.g., 2.45 GHz device)<br>☐ Laser therapy (e.g., CO_2 laser)<br>☐ Ultrasound therapy (e.g., handheld therapeutic ultrasound)<br>☐ Hot water therapy (e.g., warm water bag or hydrotherapy)<br>☐ Other (specify)<br>☐ Unknown<br>(If more than one was applied, select all. A follow-up question will ask for the specific device or subtype.)
Specify other type of thermotherapy	__________________________________
Specify the RF thermotherapy device used	◯ ThermoMed<br>◯ Other RF device (specify)
Specify other RF device	__________________________________
Specify the hand-held exothermic crystallization device used	◯ HECT-CL<br>◯ Other exothermic device (specify)

Specify other Hand-held exothermic crystallization device	__________________________
Specify the type of laser therapy used	◯ CO_2 laser<br>◯ Pulsed dye laser (PDL)<br>◯ Nd:YAG laser<br>◯ Erbium glass laser<br>◯ Diode laser<br>◯ Intravenous laser blood irradiation (ILBI)<br>◯ Other laser type (specify)
Specify other type of laser therapy	__________________________
Specify the ultrasound therapy device used	◯ Standard handheld therapeutic ultrasound device<br>◯ Other (specify)
Specify other ultrasound device	__________________________
Specify details on how the thermotherapy was performed (e.g., method, device, manufacturer)	__________________________<br>(Describe the procedure, duration, number of sessions, temperature used, and manufacturer/model if available (e.g., hot pack type, infrared lamp, microwave device).)
How many sessions of thermotherapy were performed?	__________________________
How often was thermotherapy performed?	◯ Every week<br>◯ Every 2 weeks<br>◯ Every 3 weeks<br>◯ Other
Specify how often was thermotherapy performed	__________________________

Combination therapy

Combination therapy?	◯ Yes<br>◯ No<br>◯ Unknown
Timing of combination therapy administration	◯ Concomitant administration (started at the same time)<br>◯ Consecutive administration without gap (second treatment started immediately after first ended)<br>◯ Consecutive administration with gap (second treatment started after a time gap)<br>◯ Unknown<br>(Consecutive administration example: first cryotherapy, then immediately or after a time gap amphotericin B)

Which combination treatment was used?

- ◯ Cryotherapy + Intralesional meglumine antimoniate (Glucantime)
- ◯ Liposomal Amphotericin B (Ambisome) + Intralesional meglumine antimoniate (Glucantime)
- ◯ Liposomal Amphotericin B (Ambisome) + Cryotherapy
- ◯ Liposomal Amphotericin B (Ambisome) + Miltefosine
- ◯ Intravenous meglumine antimoniate + pentoxifylline
- ◯ Other (specify)

Specify treatment combination

____

Please indicate the order in which the treatments were administered. If treatments were given at the same time, check the same column for each.

	First	Second	Third
Liposomal Amphotericin B (Ambisome)	☐	☐	☐
Intralesional meglumine antimoniate (Glucantime)	☐	☐	☐
IV/IM Meglumine antimoniate (Glucantime)	☐	☐	☐
Miltefosine	☐	☐	☐
Pentamidine	☐	☐	☐
Intralesional sodium stibogluconate (SSG)	☐	☐	☐
IV/IM sodium stibogluconate (SSG)	☐	☐	☐
Ketoconazole	☐	☐	☐
Fluconazole	☐	☐	☐
Ointment paromomycin (15%) and methylbenzethonium (12%)	☐	☐	☐
Cryotherapy	☐	☐	☐
Thermotherapy	☐	☐	☐
Other	☐	☐	☐

Was this treatment course completed as prescribed?

- ◯ Yes - Completed full treatment course as prescribed
- ◯ No - Did not complete treatment
- ◯ Partial - Completed most, but not full prescribed course
- ◯ Unknown

If not completed, what was the main reason?

- ◯ Adverse event
- ◯ Poor compliance (non-adherence)
- ◯ Loss to follow-up
- ◯ Physician decision (e.g., early stop due to response)
- ◯ Other (specify)

Specify other reason why treatment was not completed as prescribed

____

Bacterial superinfection treatment

Was a bacterial culture performed on a sample taken from the lesion?	◯ No<br>◯ Yes, negative result<br>◯ Yes, positive result (specify identified microorganism)<br>◯ Unknown
Which microorganism(s) was identified?	◯ Staphylococcus aureus<br>◯ Streptococcus pyogenes (Group A Streptococcus)<br>◯ Pseudomonas aeruginosa<br>◯ Escherichia coli<br>◯ Klebsiella pneumoniae<br>◯ Proteus spp.<br>◯ Polymicrobial (specify)<br>◯ Other (specify)
Please specify bacteria in case of polymicrobial or other	__________________________
Which treatment was administered for bacterial superinfection?	☐ Systemic antibiotics<br>☐ Topical antibiotics<br>☐ None<br>☐ Unknown
Start date of antibiotic administration	__________________________
Which systemic antibiotic was administered?	◯ Anti-staphylococcal penicillin (e.g., cloxacillin, nafcillin)<br>◯ Broad-spectrum penicillin (e.g., amoxicillin-clavulanate, piperacillin-tazobactam)<br>◯ First/second generation cephalosporin (e.g., cefazolin, cephalexin, cefuroxime)<br>◯ Other beta-lactam antibiotic<br>◯ Macrolide (e.g., azithromycin, clarithromycin)<br>◯ Quinolone (e.g., moxifloxacin, levofloxacin)<br>◯ Other (specify)<br>◯ None
Specify other systemic antibiotic	__________________________
Duration of systemic antibiotic treatment (days)	__________________________
Which topical antibiotic was administered?	◯ Fusidic acid<br>◯ Mupirocin<br>◯ Gentamicin<br>◯ Neomycin<br>◯ Clindamycin<br>◯ Erythromycin<br>◯ Metronidazole<br>◯ Other (specify)
Specify other topical antibiotic	__________________________
Duration of topical antibiotic treatment (days)	__________________________

Other treatments

Did you prescribe other concomitant treatment?

- ◯ Yes
- ◯ No
- ◯ Unknown

Which other treatments were prescribed?

- ☐ Paracetamol
- ☐ NSAIDs (e.g. ibuprofen, naproxen, celecoxib)
- ☐ Antihistamines (e.g., loratadine, diphenhydramine)
- ☐ Topical corticosteroids (e.g., hydrocortisone, betamethasone)
- ☐ Systemic corticosteroids (e.g., prednisone, dexamethasone)
- ☐ Opioids (e.g. tramadol, morphine, oxycodone, fentanyl)
- ☐ Pentoxifylline
- ☐ Other

Specify other concomitant treatment

____

Comments

____

FU treatment-related adverse events

⚠

Treatment-Related Adverse Events - Important Instructions

This form is repeatable. Use it to record each individual adverse event (AE) observed during or after treatment.

 How to repeat the form:

You can create a new instance of this form in either of the following ways:

- From the Record Home Page, click the button located next to the status icon of the Treatment-related adverse events form.
- Within the form itself, click the "+ Add new" button found at the top of the page under the "Current Instance" menu. For each adverse event, record:

- Suspected treatment(s) involved
- Type of side event
- Date of onset and resolution
- Severity and clinical outcome
- Any action taken (e.g., dose modification, treatment stopped)

☑

This structure allows accurate tracking and linkage of AEs to specific treatments.
⚠

If no treatment has been registered for the patient, the adverse event instrument will remain empty. In that case, no fields need to be completed and the form should be left blank.

Side effects of Leishmania treatment

Was a treatment-related adverse event registered?	◯ Yes<br>◯ No<br>◯ Unknown
Treatment most likely associated with this adverse event	☐ Liposomal Amphotericin B (Ambisome)<br>☐ Intralesional meglumine antimoniate (Glucantime)<br>☐ Intramuscular/intravenous meglumine antimoniate (Glucantime)<br>☐ Miltefosine<br>☐ Pentamidine<br>☐ Intralesional sodium stibogluconate (SSG)<br>☐ Intramuscular/intravenous sodium stibogluconate (SSG)<br>☐ Ketoconazole<br>☐ Fluconazole<br>☐ Ointment paromomycin (15%) and methylbenzethonium (12%)<br>☐ Cryotherapy<br>☐ Thermotherapy<br>☐ Systemic antibiotics<br>☐ Topical antibiotics<br>☐ Other<br>☐ Unknown

Which type of side effect?	◯ Local side effects<br>◯ Systemic side effects<br>◯ Unknown
Date of side effects onset	__________________________________
Describe local side effects	☐ Pain at lesion or injection site<br>☐ Redness at lesion or injection site<br>☐ Swelling at lesion or injection site<br>☐ Itching at lesion or injection site<br>☐ Burning sensation at lesion or injection site<br>☐ Ulceration or worsening of local wound<br>☐ Localized infection (clinical suspicion or culture-confirmed)<br>☐ Other (specify)
Describe other local side effects	__________________________________
Type of systemic side effects	◯ Dermatologic toxicity<br>◯ Hematologic toxicity<br>◯ Metabolic or electrolyte disturbance<br>◯ Hypersensitivity or anaphylaxis<br>◯ Cardiotoxicity<br>◯ Hepatotoxicity<br>◯ Renal toxicity<br>◯ Gastrointestinal symptoms<br>◯ Neurologic or neuropsychiatric toxicity<br>◯ Pancreatitis<br>◯ Infusion-related reaction<br>◯ Other (specify)<br>◯ Unknown
Describe other systemic side effects	__________________________________
Specify dermatologic toxicity	☐ Rash (maculopapular, erythematous, etc.)<br>☐ Skin ulceration<br>☐ Pruritus<br>☐ Dry skin<br>☐ Hyperpigmentation or discoloration<br>☐ Alopecia<br>☐ Other (specify)
Specify other dermatologic toxicity	__________________________________
Specify hematological toxicity	☐ Anemia<br>☐ Leukopenia<br>☐ Neutropenia<br>☐ Lymphopenia<br>☐ Thrombocytopenia<br>☐ Pancytopenia<br>☐ Other (specify)
Specify other hematological toxicity	__________________________________

Specify metabolic or electrolyte abnormality	☐ Hypokalemia<br>☐ Hyponatremia<br>☐ Hyperkalemia<br>☐ Hyperglycemia<br>☐ Hypoglycemia<br>☐ Acidosis<br>☐ Alkalosis<br>☐ Other (specify)
Specify other metabolic or electrolyte abnormality	__________________________________
Specify hypersensitivity reaction	☐ Anaphylaxis<br>☐ Urticaria<br>☐ Facial or airway edema<br>☐ Bronchospasm<br>☐ Flushing<br>☐ Hypotension<br>☐ Other (specify)
Specify other hypersensitivity reaction	__________________________________
Specify cardiac event	☐ QT interval prolongation<br>☐ Bradycardia<br>☐ Sinus or supraventricular tachycardia<br>☐ Atrial fibrillation or flutter<br>☐ AV block or conduction abnormality<br>☐ Myocarditis or pericarditis<br>☐ Heart failure<br>☐ Chest pain (suspected cardiac origin)<br>☐ Other (specify)
Specify other cardiac toxicity	__________________________________
Specify type of liver test abnormality or clinical finding	☐ Elevated ALT and/or AST<br>☐ Elevated ALP (FAL)<br>☐ Elevated GGT<br>☐ Elevated bilirubin<br>☐ Jaundice<br>☐ Clinical hepatitis symptoms (e.g., fatigue, anorexia)<br>☐ Other (specify)
Specify other hepatotoxicity	__________________________________
Specify renal toxicity	☐ Elevated creatinine (lab increase not meeting AKI criteria)<br>☐ Proteinuria<br>☐ Hematuria<br>☐ Acute kidney injury (AKI) (creatinine ↑ ≥1.5× baseline and/or oliguria)<br>☐ Other (specify)
Specify other renal toxicity	__________________________________

Specify gastrointestinal toxicity	☐ Nausea<br>☐ Vomiting<br>☐ Diarrhea<br>☐ Abdominal pain<br>☐ Mucositis or oral ulcers<br>☐ Constipation<br>☐ Anorexia<br>☐ Other (specify)
Specify other gastrointestinal toxicity	__________________________________
Specify neurological or psychiatric event	☐ Headache<br>☐ Dizziness or vertigo<br>☐ Paresthesia or numbness<br>☐ Seizure<br>☐ Confusion or disorientation<br>☐ Tremor or ataxia<br>☐ Visual disturbances<br>☐ Mood or behavioral changes<br>☐ Other (specify)
Specify other neurological or psychiatric event	__________________________________
Specify infusion-related symptoms	☐ Fever<br>☐ Chills<br>☐ Hypotension<br>☐ Back pain<br>☐ Flushing<br>☐ Dyspnea<br>☐ Other (specify)
Specify other infusion-related symptoms	__________________________________
Severity of the side effect	○ Mild - no interference with activity<br>○ Moderate - some interference, no hospitalization<br>○ Severe - prevents daily activity, may require hospitalization<br>○ Life-threatening - urgent intervention required<br>○ Death<br>○ Unknown
Date of side effect resolution	__________________________________
Date of death	__________________________________
How was the adverse event managed?	☐ No action needed<br>☐ Symptomatic treatment (e.g., antihistamines, NSAIDs)<br>☐ Treatment temporarily interrupted<br>☐ Treatment permanently discontinued<br>☐ Switched to alternative treatment<br>☐ Referred for specialist evaluation or hospitalization<br>☐ Unknown

Regarding suspected treatment-related bacterial superinfection

Was a bacterial culture performed on a sample taken from the lesion?	◯ No<br>◯ Yes, negative result<br>◯ Yes, positive result (specify identified microorganism)<br>◯ Unknown
Which microorganism(s) was identified?	◯ Staphylococcus aureus<br>◯ Streptococcus pyogenes (Group A Streptococcus)<br>◯ Pseudomonas aeruginosa<br>◯ Escherichia coli<br>◯ Klebsiella pneumoniae<br>◯ Proteus spp.<br>◯ Polymicrobial (specify)<br>◯ Other (specify)
Please specify bacteria in case of polymicrobial or other	__________________________
Which treatment was administered for bacterial superinfection?	☐ Systemic antibiotics<br>☐ Topical antibiotics<br>☐ None<br>☐ Unknown
Start date of antibiotic administration	__________________________
Which systemic antibiotic was administered?	◯ Anti-staphylococcal penicillin (e.g., cloxacillin, nafcillin)<br>◯ Broad-spectrum penicillin (e.g., amoxicillin-clavulanate, piperacillin-tazobactam)<br>◯ First/second generation cephalosporin (e.g., cefazolin, cephalexin, cefuroxime)<br>◯ Other beta-lactam antibiotic<br>◯ Macrolide (e.g., azithromycin, clarithromycin)<br>◯ Quinolone (e.g., moxifloxacin, levofloxacin)<br>◯ Other (specify)<br>◯ None
Specify other systemic antibiotic	__________________________
Duration of systemic antibiotic treatment (days)	__________________________
Which topical antibiotic was administered?	◯ Fusidic acid<br>◯ Mupirocin<br>◯ Gentamicin<br>◯ Neomycin<br>◯ Clindamycin<br>◯ Erythromycin<br>◯ Metronidazole<br>◯ Other (specify)
Specify other topical antibiotic	__________________________
Duration of topical antibiotic treatment (days)	__________________________

Final outcome

FInal outcome registration - Important Instructions

The final outcome should be assessed at least 6 months ±4 weeks after treatment initiation.

If the last follow-up visit occurred before this time point, please indicate the reason by selecting the appropriate option:

No follow-up visits performed after initial cure assessment, per clinician decision (favorable clinical response; further follow-up not scheduled unless deterioration occurred)

Lost to follow-up

Date of final outcome assessment

____
(Final assessment should be performed (preferably) 6-12 months after treatment initiation)

Final outcome assessment

Final outcome

- ☐ Cure: complete re-epithelization/flattening of all lesions
- ☐ Treatment failure: absence of total re-epithelization/flattening of all lesions by end of follow-up
- ☐ Relapse: recurrence at the same site or new lesions elsewhere caused by the same parasite after prior documented cure
- ☐ Worsening after initial improvement (without reaching complete cure)
- ☐ Death
- ☐ No follow-up visits performed after initial cure assessment, per clinician decision (favorable clinical response; further follow-up not scheduled unless deterioration occurred)
- ☐ Lost to follow-up
- ☐ Unknown

(Select ALL that apply. For example, select both "Cure" and "Relapse" if the patient experienced a relapse after documented complete cure but was ultimately cured following subsequent therapy.)

Date of complete cure

____

Features of treatment failure

- ☐ Persistence of the largest lesion despite overall size reduction
- ☐ Lesion(s) persisted without reduction
- ☐ Lesion(s) increased in size
- ☐ New lesions appeared
- ☐ Worsening of inflammation
- ☐ Mucosal involvement developed
- ☐ Only some lesions cured

Number of lesions cured

____

Number of relapses during the follow-up	________________________________
Date of first relapse	________________________________
Date of second relapse	________________________________
Date of third relapse	________________________________
Date of 4th relapse	________________________________
Date of 5th relapse	________________________________
Date of death	________________________________
Cause of death	◯ Complications secondary to CL/MCL<br>◯ Other medical condition(s)<br>◯ Severe side effects associated with CL/MCL treatment<br>◯ Other reason not related to medical conditions<br>◯ Unknown
Please specify the cause of death	________________________________
Clinical sequelae (scarring and pigment changes)	☐ No visible scar or pigment change<br>☐ Hyperpigmentation<br>☐ Hypopigmentation<br>☐ Atrophic scar<br>☐ Hypertrophic/keloid scar<br>☐ Unknown<br>(Select ALL that apply. Each lesion may have a different scarring/pigment change pattern.)
Grading of hyperpigmentation	

Source: Ranasinghe S et al. (2024) Development of a Core Outcome Measure Instrument; "LeishCOM_LCL", for Localised Cutaneous Leishmaniasis. PLOS Neglected Tropical Diseases 18(8): e0012393. https://doi.org/10.1371/journal.pntd.0012393

◯ A: no hyperpigmentation
◯ B: mild hyperpigmentation
◯ C: moderate hyperpigmentation
◯ D: severe hyperpigmentation
◯ Unknown
(If multiple areas of hyperpigmentation are present with different severities, select the category that corresponds to the most severe area.)

Grading of hypopigmentation

Source: Ranasinghe S et al. (2024) Development of a Core Outcome Measure Instrument; "LeishCOM_LCL", for Localised Cutaneous Leishmaniasis. PLOS Neglected Tropical Diseases 18(8): e0012393. https://doi.org/10.1371/journal.pntd.0012393

◯ A: no hypopigmentation
◯ B: mild hypopigmentation
◯ C: moderate hypopigmentation
◯ D: severe hypopigmentation
◯ Unknown
(If hypopigmentation varies between lesions, choose the grade that corresponds to the most pronounced (lightest) area.)

Grading of atrophic scarring

Source: Ranasinghe S et al. (2024) Development of a Core Outcome Measure Instrument; "LeishCOM_LCL", for Localised Cutaneous Leishmaniasis. PLOS Neglected Tropical Diseases 18(8): e0012393. https://doi.org/10.1371/journal.pntd.0012393

◯ A: no atrophic scarring
◯ B: mild atrophic scarring
◯ C: moderate atrophic scarring
◯ D: severe atrophic scarring
◯ Unknown
(If multiple scars show varying degrees of atrophy, grade based on the area with the deepest or most extensive atrophic change.)

Grading of hypertrophic/keloid scarring

Source: Ranasinghe S et al. (2024) Development of a Core Outcome Measure Instrument; "LeishCOM_LCL", for Localised Cutaneous Leishmaniasis. PLOS Neglected Tropical Diseases 18(8): e0012393. https://doi.org/10.1371/journal.pntd.0012393

	◯ A: no atrophic scarring<br>◯ B: mild atrophic scarring<br>◯ C: moderate atrophic scarring<br>◯ D: severe atrophic scarring<br>◯ Unknown<br>(If multiple scars show varying degrees of atrophy, grade based on the area with the deepest or most extensive atrophic change.)
Longest diameter of the largest scar/pigment change area (mm)	__________________________
Can you provide a photograph of the site of the initial largest lesion (or its scar) at final outcome assessment?	
Functional sequelae attributable to mucosal leishmaniasis	☐ Nasal obstruction or deformity (e.g., septal perforation, saddle nose)<br>☐ Dysphonia or voice changes (e.g., hoarseness)<br>☐ Dysphagia<br>☐ Dyspnea<br>☐ Other documented functional impairment (specify)<br>☐ No functional sequelae observed<br>☐ Not assessed / information not available
Specify other functional impairment	__________________________

Hospitalization

Did the patient require hospitalization during follow-up?	◯ Yes<br>◯ No<br>◯ Unknown<br>(Hospitalization ONLY for treatment administration should not be considered)
Date of hospitalization	__________________________
Reason(s) for hospitalization during follow-up	☐ Severe adverse event related to treatment<br>☐ Treatment-related toxicity (e.g., nephrotoxicity, hepatotoxicity)<br>☐ Severe worsening of lesions<br>☐ Severe secondary bacterial infection of lesions<br>☐ New serious comorbidity unrelated to leishmaniasis (e.g., myocardial infarction, stroke)<br>☐ Other (specify)
Specify other reason for hospitalization	__________________________
How long was the patient hospitalized (days)?	__________________________

Other complications

Any other complication during the course of the disease?	◯ Yes<br>◯ No
Please specify	__________________________

Other infectious diseases

Was the patient diagnosed with any other infectious disease?	◯ Yes<br>◯ No
Which one(s)?	☐ HIV<br>☐ TB<br>☐ Strongyloidiasis<br>☐ Schistosomiasis<br>☐ Myasis<br>☐ Other
Specify other infectious disease	__________________________

Other relevant additional investigations

Any other relevant additional investigation during the course of the disease?	◯ Yes<br>◯ No<br>(Other relevant diagnosis or complementary test results)
Please specify	__________________________
Comment(s)	________________________________

Variables not included

Previously included variables ______________________

Clinical Form Definition Key Features
Localized Cutaneous Leishmaniasis (LCL) Single or few (< 10) skin lesions located near the sandfly bite site.
Typically painless ulcers with raised borders or nodules.
Regional lymphadenopathy possible.
Diffuse Cutaneous Leishmaniasis (DCL) Numerous, widespread, non-ulcerative nodules and plaques due to impaired cellular immunity. Non-ulcerated, infiltrative lesions.
No mucosal involvement.
Heavy parasite load.
Disseminated Cutaneous Leishmaniasis More than 10 lesions on multiple non-contiguous areas, often with mucosal risk. Multiple body areas affected.
Mucosal involvement possible.
Leishmaniasis Recidivans (Recurring CL) Chronic recurrent lesions appearing at the margins of old scars.
Reactivation months/years after healing.
Persistent papules/nodules.
Often facial involvement.

Clinical form of Cutaneous Leishmaniasis

- ◯ Localized cutaneous leishmaniasis (LCL)
- ◯ Diffuse cutaneous leishmaniasis (DCL)
- ◯ Disseminated cutaneous leishmaniasis
- ◯ Leishmaniasis recidivans (Recurring cutaneous leishmaniasis)
- ◯ Unknown

Skin scraping PCR test

- ◯ Negative
- ◯ Positive
- ◯ Inconclusive
- ◯ Not done
- ◯ Unknown

Skin scraping culture

- ◯ Negative
- ◯ Positive
- ◯ Inconclusive
- ◯ Not done
- ◯ Unknown

Skin scraping microscopy

- ◯ Negative
- ◯ Positive
- ◯ Inconclusive
- ◯ Not done
- ◯ Unknown

Skin biopsy PCR test

- ◯ Negative
- ◯ Positive
- ◯ Inconclusive
- ◯ Not done
- ◯ Unknown

Skin biopsy culture

- ◯ Negative
- ◯ Positive
- ◯ Inconclusive
- ◯ Not done
- ◯ Unknown

Skin biopsy microscopy	◯ Negative<br>◯ Positive<br>◯ Inconclusive<br>◯ Not done<br>◯ Unknown
Skin aspirate PCR test	◯ Negative<br>◯ Positive<br>◯ Inconclusive<br>◯ Not done<br>◯ Unknown
Skin aspirate culture	◯ Negative<br>◯ Positive<br>◯ Inconclusive<br>◯ Not done<br>◯ Unknown
Skin aspirate microscopy	◯ Negative<br>◯ Positive<br>◯ Inconclusive<br>◯ Not done<br>◯ Unknown
Skin aspirate PCR test	◯ Negative<br>◯ Positive<br>◯ Inconclusive<br>◯ Not done<br>◯ Unknown
Route of Leishmania treatment administration	☐ Topical<br>☐ Intralesional<br>☐ Oral<br>☐ Intramuscular<br>☐ Intravenous<br>☐ Other<br>☐ Unknown
Other route of administration	__________________________________
Route of Leishmania treatment administration	☐ Topical<br>☐ Intralesional<br>☐ Oral<br>☐ Intramuscular<br>☐ Intravenous<br>☐ Other<br>☐ Unknown
Other route of Leishmania treatment administration	__________________________________
